# Genetic conversion of a split-drive into a full-drive element

**DOI:** 10.1038/s41467-022-35044-4

**Published:** 2023-01-12

**Authors:** Gerard Terradas, Jared B. Bennett, Zhiqian Li, John M. Marshall, Ethan Bier

**Affiliations:** 1 Department of Cell and Developmental Biology, University of California, San Diego, La Jolla, CA 92093 USA; 2 Tata Institute for Genetics and Society, University of California, San Diego, La Jolla, CA 92093 USA; 3 Biophysics Graduate Group, Division of Biological Sciences, College of Letters and Science, University of California, Berkeley, CA 94720 USA; 4 Divisions of Epidemiology and Biostatistics, School of Public Health, University of California, Berkeley, CA 94720 USA; 5 Innovative Genomics Institute, Berkeley, CA 94720 USA; 6 Present Address: Department of Entomology, The Center for Infectious Disease Dynamics, and the Huck Institutes for the Life Sciences, The Pennsylvania State University, University Park, PA 16802 USA

**Keywords:** CRISPR-Cas9 genome editing, Synthetic biology

## Abstract

The core components of CRISPR-based gene drives, Cas9 and guide RNA (gRNA), either can be linked within a self-contained single cassette (full gene-drive, fGD) or be provided in two separate elements (split gene-drive, sGD), the latter offering greater control options. We previously engineered split systems that could be converted genetically into autonomous full drives. Here, we examine such dual systems inserted at the *spo11* locus that are recoded to restore gene function and thus organismic fertility. Despite minimal differences in transmission efficiency of the sGD or fGD drive elements in single generation crosses, the reconstituted *spo11* fGD cassette surprisingly exhibits slower initial drive kinetics than the unlinked sGD element in multigenerational cage studies, but then eventually catches up to achieve a similar level of final introduction. These unexpected kinetic behaviors most likely reflect differing transient fitness costs associated with individuals co-inheriting Cas9 and gRNA transgenes during the drive process.

## Abstract (web summary)

CRISPR-based gene-drives can carry the Cas9 and guide RNA (gRNA) components in a single-linked cassette or in separate elements inserted into different genomic loci. Here the authors genetically transform and compare full versus split drives, with the former performing less efficiently than predicted.

## Introduction

CRISPR-based gene drives offer novel approaches for vector control by altering the genetic structures of wild insect populations^1–5^. Linked gene drives (or full gene drives, fGD) carry gene cassettes encoding the bacterial Cas9 endonuclease and a guide RNA (gRNA) sequence that directs the Cas9/gRNA ribonucleoprotein complex to cleave the genome at the site of cassette insertion^3–5^. Upon cleavage of the target site on a homologous chromosome, the gene-drive element is copied into the double stranded break (DSB) by homology-directed repair (HDR) using the drive-bearing homologous chromosome as a repair template. If this directional gene conversion process is efficient, the drive allele will be inherited in a super-Mendelian fashion (>50%). Genes encoding beneficial factors can be linked to fGD cassettes to spread these traits into susceptible populations within a few generations^3^, following a trajectory defined by logistical (initially exponential) growth kinetics^4^.

Gene-drive technologies can be used either to modify or suppress insect disease vector populations, depending on their design. Modification drives^3,6,7^ carry beneficial cargo to reduce the vector capacity of the insect, while suppression drives^5,8,9^ endeavor to bias the inheritance of deleterious traits that will ultimately either kill or sterilize the insect. In addition to autonomously acting fGDs in which Cas9 and gRNA are encoded within a unitary cassette, so-called split gene drives (sGD) have been developed^10–12^ in which the copying cassette carries only the gRNA component, with Cas9 supplied from a second genomic site. In such bipartite arrangements, the gRNA-carrying cassette is copied in the presence of the Cas9 source while the Cas9-encoding element remains static (i.e., transmitted in a standard Mendelian fashion). In the absence of Cas9, however, the gRNA-bearing split-drive element also is inherited at Mendelian frequencies. When at low frequencies, sGDs are predicted to spread additively through populations rather than exponentially, as do fGDs^4^.

Our group recently analyzed a set of sGDs inserted into essential loci carrying recoded cDNA sequences that restore functionality of the endogenous genes upon allelic cleavage and conversion^12^. Such recoded sGDs minimize the production of nonfunctional NHEJ alleles that are resistant to Cas9 cleavage by dominantly eliminating such alleles transmitted by females through a phenomenon referred to as sterile/lethal mosaicism^6,13^. This latter process arises as a consequence of maternal inheritance of Cas9/gRNA complexes that act on the paternal allele to mutate it in a sufficient fraction of cells in progeny to either kill (lethal mosaicism) or sterilize (sterile mosaicism) such offspring.

Although unexploited in the previous study^12^, the sGD constructs included another design feature that permits facile genetic transfiguration of the sGD into an fGD. This conversion system relies on a set of Cas9 sources that carry sGD homology arms flanking the Cas9 cassette and a gRNA (gRNAHack) that can cleave a synthetic target site placed within the sGD element. When such Mendelian Cas9 sources (inserted into AttP sites in different genomic locations) are crossed to the sGD element, the gRNAHack mediates cleavage of the sGD resulting in the insertion of the Cas9 cargo into the recipient element. Here, we provide proof-of-concept for such conversion of a recoded split drive into a full drive inserted into the *spo11* locus, which is required for fertility in both sexes. This strategy for converting a sGD into a fGD follows the logic of the homology-assisted CRISPR knock-in system (or HACK)^14^, which efficiently replaces target loci in *Drosophila*. We evaluate the performance of a converted (or hacked) fGD in both single-generation crosses and cage competition experiments and compare its performance to that of the sGD from the previous experiments^12^. Surprisingly, instead of spreading rapidly as predicted, we find that the fGD displays slower initial drive kinetics than the sGD, although it eventually catches up and achieves a similar level of introduction into the population. The delayed drive of the fGD element may result from elevated transient fitness costs associated with co-inheritance of Cas9 and gRNA transgenes in the presence of cleavage-sensitive target alleles that exceed those produced by the sGD configuration^12^.

## Results

### Design of convertible sGD elements and corresponding Cas9 lines

**Fig. 1:**
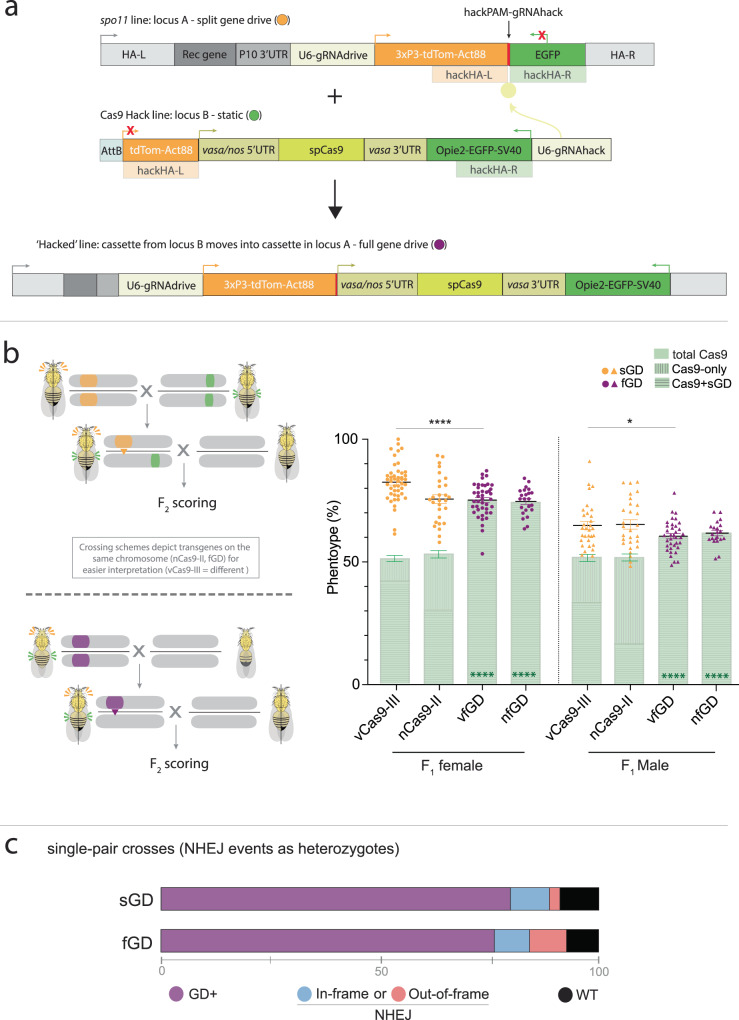
Super-Mendelian performance is comparable between split and full gene-drive elements in single-generation crosses. **a** Schematic of the genetic constructs engineered and tested in the study for sGD-to-fGD conversion. A tdTom-expressing split gene-drive cassette (sGD) that contains a hackPAM in between its markers is genetically paired with a specific EGFP-expressing Cas9, which contains a gRNA (that does not home) targeting the sequence next to the hackPAM in sGD, that drives itself into the sGD locus and forms an autonomous full gene-drive cassette (fGD) using marker sequences as homology arms. The resulting cassette expresses both tdTom and EGFP markers. **b** Upon successful hacking of the *spo11* sGD, *vasa* and *nos-*driven fGDs (vfGD and nfGD, respectively) were assessed for F_1_ germline conversion capacities. The panel follows the layout described in (**a**), except for the sGD marker being tdTomato and represented in orange and purple for sGD and fGD configurations, respectively. Sex of the parental (F_1_) trans-heterozygote is indicated in the *X* axis under the Cas9 line used, as well as by circles (female) or triangles (male), used to show the data of each individual cross. Error bars represent mean values ± SEM. Stars represent statistical significance (****P < 0.0001) on differences in conversion efficiency between sGD and fGD (black, two-sided *t* test) and Cas9 deriving from Mendelian frequencies in fGD (green, *χ*^2^). For vfGD, *n* = 2854 (*N* = 42) for female crosses and *n* = 1827 (*N* = 36) for male crosses. For nfGD, *n* = 1015 (*N* = 23) and *n* = 990 (*N* = 21), respectively. Source Data are provided as a Source Data file. sGD data, which are reproduced from a previous study^12^,  serve as a comparison for the fGD. **c** Regions surrounding the *spo11* gRNA target site were amplified from a small subset (32 for sGD, 18 for fGD) of single non-fluorescent F_2_ individuals generated in (**b**), sequenced by Sanger and analyzed. A bar depicts the % of GD^+^ (purple) and % of non-fluorescent (GD^−^, gray) flies. The prevalence of NHEJ genotypes is shown in blue or red, describing the kind of NHEJ allele that is formed and % among the total tested GD^−^ (NHEJ/WT) heterozygotes. We note that, even though they are likely to very rare, large deletions affecting primer binding (>100 bp deletions) would be classified as WT due to the detection of only the parental WT allele.

Previously, we engineered a recoded *spo11* sGD line^12^ that copied efficiently to the homologous chromosome in females (76–83%) but to a lesser degree in males (65–69%) in single-generation crosses. This *spo11* sGD drove to a high level of stable introduction in multigenerational cage experiments (~85%). Because the *spo11* sGD drive performed well but not perfectly, we selected it to test for conversion to an fGD by hacking (Fig. 1a and Supplementary Fig. S1a) since it would permit a fine-scale comparison of sGD versus fGD performance parameters. As a first step, we generated Cas9-donor lines in which *vasa* or *nanos* (*nos*)-driven Cas9 transgenes were placed between a 3′ partial nonfunctional fragment of a 3xP3-tdTomato (expressed in the eye) marker and a fully active Opie2-EGFP transgene (expressed in the abdomen). These flanking sequences act as homology arms (HA) adjacent to a gRNA cleavage site located in the sGD element (Fig. 1a, *spo11* line). The sGD carries a synthetic PAM site (hackPAM) between the two marker sequences that is unique to those constructs. One of the marker transgenes encodes an intact fluorescent protein (tdTomato) and is active in the sGD line, whereas the other marker gene (EGFP) consists only of partial 3′ sequences and is inactive. The hackPAM provides a synthetic recognition site for the hacking gRNA carried outside of the homology arms on the Cas9-donor (Cas9Hack) element (Fig. 1a, hacked line). Cas9Hack lines contain a gRNA (gRNAHack) that targets the hackPAM site in the target sGD line and mediates insertion of Cas9 into the locus only when the independent tdTom^+^ sGD and EGFP^+^ Cas9Hack elements are combined (Fig. 1a and Supplementary Fig. S1a). Since gRNAHack sequences are located outside of the HA-flanked Cas9 element, they do not mobilize into the sGD recipient construct (Fig. 1a). Successful CRISPR-mediated gene conversion leads to the insertion of the Cas9 element and remaining EGFP sequences into the sGD, which displays linked dual markers (tdTom^+^, EGFP^+^) and autonomous self-mobilizing capacity as a composite fGD element. Donor Cas9Hack transgenes carrying the gRNAHack components were inserted into available fly strains containing AttP sites located either on chromosome II or chromosome III via phiC31 recombination^15^ (Supplementary Fig. S1b).

### Cas9Hack and control Cas9 lines sustain comparable copying frequencies of a reference sGD

We used two previously validated autosomal static Cas9 sources (nCas9-II and vCas9-III), inserted into chromosomes II and III, respectively, as reference controls for assessing the activity of Cas9Hack sources controlled by the same promoters as the Hack lines (vHack and nHack). These lines carry the same regulatory sequences to Cas9 as the static sources but can mediate hacking events by gRNAHack when coupled with sGD lines (Fig. 1a). We first tested the newly generated Hack-A lines (located at the “A” locus, 55C4; Supplementary Fig. S1b) for their ability to support super-Mendelian inheritance of a reference unhackable mCerulean-marked split-drive element inserted in the *vasa* locus on chromosome II (CC*vasa*; Supplementary Figs. S1b and S2). For these initial experiments, we chose not to use the hackPAM-bearing *spo11* sGD to avoid complications arising from gRNAHack cutting the target element simultaneously with allelic conversion and copying of that element (via its own gRNA). We observed comparable allelic conversion efficiencies for CC*vasa* using either the Mendelian sources (vCas9-III and nCas9-II) or Cas9Hack transgenes when expressed using the corresponding promoters (vHack-A and nHack-A; Supplementary Fig. S2). F_2_ progeny derived from heterozygous CC*vasa*/Cas9 males displayed lower conversion frequencies than females, as previously observed for several split-drive systems in *Drosophila*, which may be related to the anomalous absence of male recombination in this species^11^. We note that the presence of an additional U6-gRNA element (U6-gRNAHack) carried by the Cas9Hack sources had no target in these specific crosses and did not affect CC*vasa* copying efficiencies. As expected based on the static nature of the tested Cas9 sources, inheritance of the Cas9 transgenes in all cases approximated Mendelian frequencies (average among conditions: 51 ± 8%; Supplementary Fig. S2). We conclude that there are no significant differences in activity of between the Cas9Hack sources and our reference Cas9 control elements using the same promoters (*vasa* or *nos*) to express Cas9.

### Generation of *spo11* fGD lines

Having confirmed the expected performance of the Cas9Hack lines, we next used these lines to generate fGD versions of the* spo11* sGD. We crossed the hackable *spo11* sGD with both vHack-A and nHack-A to obtain *spo11* fGDs (Fig. 1a and Supplementary Fig. S1a). Of the six crosses, we performed for each hacking element, we recovered double-marked/CyO individuals in 75% of cases (5/6 for vHack-A, 4/6 for nHack-A). The predicted genomic structures of *spo11* hacked cassettes were verified for fGD elements (hacked either with *vasa-*Cas9: vfGD or *nos-*Cas9: nfGD) using PCR amplification followed by Sanger sequencing. In contrast to recovery of these multiple independent hacking events when employing a Cas9Hack insertion near the insertion site of the *spo11* sGD, an identical vHack construct inserted at a different III chromosome AttP site (vHack-B; located at the “B” locus, 68A4; Supplementary Fig. S1b) failed to produce any successful recombination events, underscoring the previously described role of genomic position effects on heterologous gene conversion efficiencies^16^ (Supplementary Fig. S1b). Since the *spo11* sGD is closely linked to the vHack-A insertion site while the vHack-B locus is unlinked, the more efficient performance of vHack-A relative to vHack*-*B is consistent with prior observations of more efficient conversion mediated by proximal loci and Hi-C sequence ligation in which neighboring segments of the genome tend to lie closer together^16^. Consistent with this genomic proximity hypothesis, the vHack-A (2R), which efficiently sustained hacking of the closely linked *spo11* sGD, did not support conversion of the more distantly residing *rab5* sGD element in preliminary experiments (2L).

### Allelic conversion rates are similar for sGD and unitary *spo11* fGD elements

After successfully obtained hacked *spo11* fGDs carrying either *vasa*-Cas9 or *nos*-Cas9, we next compared their allelic conversion rates to those obtained with the bipartite *spo11* sGD system. We crossed G_0_ homozygote *spo11* fGD males to homozygous WT virgin females followed by mating heterozygote F_1_ siblings (male or female) to their WT counterparts and then scored the fraction of fluorescently labeled F_2_ offspring (Fig. 1b). In both sets of crosses, Cas9 was carried through males to avoid potentially confounding maternal transmission of the endonuclease, although we note that attenuated carryover of this type could occur systemically in cells of F_1_ fGD males due to multigenerational inheritance from their grandmothers^6,17^. Paralleling our observations with the *spo11* sGD, where transmission through females (vCas9-III sGD = 83 ± 8%; nCas9-II sGD = 76 ± 10%) was greater than through males (vCas9-III sGD=65 ± 9%; nCas9-II sGD = 65 ± 10%) (Fig. 1b, and Fig. 2b in ref. 12), we detected gender-specific differences in both vfGD and nfGD inheritance. Female-driven fGD transmission rates of ~75% (vfGD = 75 ± 7%, nfGD = 74 ± 6%; Fig. 1b) were slightly lower than those detected for the split sGD form for vCas9, but were comparable between the fGD and sGD for the nCas9 source. As mentioned above, transgene inheritance was substantially lower when passed through males, with only ~60% of the progeny being fGD^+^ (vfGD = 60 ± 6%, nfGD = 62 ± 4%; Fig. 1b), a decrease of ~5% relative to both the vCas9-III and nCas9-II sGD counterparts. As expected, static Cas9 sources were inherited at Mendelian frequencies in all *spo11* sGD crosses (vCas9-III = 52 ± 8% (both genders); nCas9-II = 53 ± 9% (F), 52 ± 6% (M)), whereas Cas9 remained integrally linked to the *spo11* element in the fGD (see super-Mendelian inheritance frequencies above; Fig. 1b).

**Fig. 2:**
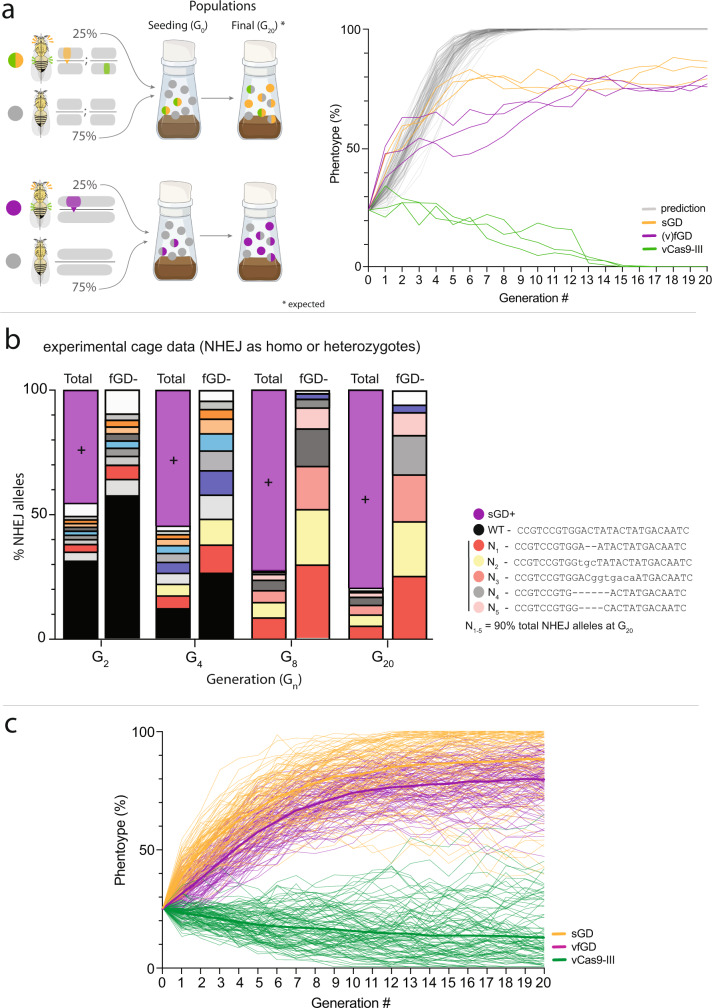
*spo11* fGD multigenerational cage trials and NHEJ profile assessment. **a** Setup of fGD cage trials followed previous experiments with the sGD-Cas9 configuration. Virgin heterozygote fGD/+ and WT (+/+) flies were seeded at 1:3 ratio in the initial generation and allowed to mate at random at each generation (G_n_). Flies in a cage were counted and scored for presence or absence of the phenotypic markers and randomly passed on to the following generation (G_n+1_). Gray traces depict the predicted fGD performance. Orange and green traces depict sGD cage experiments where transgene and Cas9 are unlinked and sort independently of one another. sGD data reproduced from a previous study^12^ serves as a comparison for the fGD. Purple traces depict fGD cage trials, where transgene and Cas9 are linked as one genomic unit. Source Data for fGD are provided as a Source Data file. **b** NHEJ cage trial data were obtained by deep-sequencing the target site region of pooled non-fluorescent individuals at specific generations. At each generation, NHEJ alleles are shown to represent their distribution among the total population (fGD^+^ and fGD^−^, left) or only for non-fluorescent fGD^−^ flies (right). Purple bars show the fGD^+^ population percentage. At G_2_ and G_4_, there are many transient alleles that disappear by G_8_, when the large array of NHEJs is simplified to a subset of, most likely, non-detrimental alleles that is maintained by G_20_. Sequences of the most prevalent NHEJ alleles at G_20_ are shown next to the graph. **c** Mathematical models on fGD cage trials were run using fitted parameter values and 100 stochastic simulations were plotted for the *spo11* fGD (purple). Thicker lines depict the mean of the 100 simulations.

As a complementary approach to assess fGD performance, we examined the in vivo cleavage efficiency of the *spo11* fGD transgene by profiling the range of different NHEJ alleles generated in single-pair crosses (Fig. 1c). We extracted DNA from non-fluorescent (fGD^−^) F_2_ offspring derived from independent crosses of an F_1_ trans-heterozygote female with a WT male. Following amplification and sequencing of the target site in single fGD^−^ F_2_ individuals, we estimated the frequencies of NHEJ-induced alleles occurring in F_1_ trans-heterozygous mothers by subtracting the paternal WT allele from the sequencing reads. The analysis of fGD^−^ flies revealed that 28% of the individuals inherited WT sequences, while 33% carried *in-frame* indels, and 39% had *out-of-frame* mutations (Fig. 1c). Based on the sequencing data and super-Mendelian conversion frequencies observed in single crosses, we estimate that the overall cleavage frequency for the *spo11* fGD was ~93%. This is very similar to the 92.3% cleavage frequency obtained for *spo11* as a sGD^12^, confirming that cleavage is target locus-specific and not dependent on the size or nature of the transgenic cargo carried by the element. However, we did observe a notable increase in the production of non-functional frameshift mutations for the fGD relative to the sGD (Fig. 1c; fGD = 39% versus sGD = 12%). We conclude that the sGD and fGD drives produce comparable levels of drive and similar profiles of NHEJ mutations.

### *spo11* sGD and fGD trajectories diverge in initial cage drive dynamics but not in final outcome

In our prior study^12^, *spo11* sGD frequencies were observed to increase steadily from 25 to 70–85% over 5–6 generations when driven through a naïve WT population by an unlinked vCas9-III source. In subsequent generations, at which point all available WT alleles had either been converted in drive or NHEJ alleles, the sGD element then remained fixed at a stable level of introduction (Fig. 2a, orange traces). This final plateau level of sGD introduction is most likely determined by the accumulation of non-deleterious drive-resistant NHEJ alleles. We also noted that Mendelian sources of Cas9 were eliminated from the population when paired with specific sGDs, which in the case of the *spo11* sGD occurred over 15 generations (Fig. 2a, green traces). We inferred that the loss of Cas9 resulted from modest fitness costs associated with carrying both Cas9 and gRNA bearing transgenes under conditions where uncleaved target alleles remained abundant^12^.

We selected the split combination of vCas9-III + sGD for further comparison in multigenerational cage trials. We reasoned that these experiments should provide insights regarding whether forced linkage of vCas9 to the gRNA within the same fGD cassette would accelerate its drive dynamics or increase its final degree of introduction as predicted based on the different inherent drive mechanisms acting in the two systems^4^. In our prior study^12^, we modeled key drive parameters, including fertility fitness costs, for the *spo11* sGD drive. In order to predict the behavior of vfGD in multigenerational cages, we modified our previous sGD model to account for Cas9 acting as a unitary element together with the sGD gRNA cassette, as well as incorporating the modest differences in conversion rates observed in single-generation crosses between the *spo11* split and full drives (Fig. 2a, gray traces). In all simulations, the vfGD reached completion within a few passages due to the higher percentage of Cas9^+^, gRNA^+^ individuals that can copy the genetic element to WT alleles during early generations (Fig. 2a, gray traces). We then tested these vfGD simulations experimentally by conducting multigenerational cage experiments paralleling those used for the sGD studies^12^. We seeded three replicate cages with 25% heterozygous (fGD/+) to 75% WT (+/+) individuals (equally mature G_0_ males and G_0_ virgin females) and randomly-selected individuals were transferred at each generation for 20 generations (Fig. 2a). Surprisingly, following an initial rise in fGD frequency, we observed a stalling period (G_4_ to G_10_ generations) during which the fGD increased only gradually in frequency without achieving the higher predicted levels of final introduction. Based on the modeling summarized above, we had expected that forcing linkage between Cas9 and gRNA would lead to a heightened performance by the fGD relative to sGD in cage trials, since any fGD^+^ individual should be able to mediate cleavage and allelic conversion of WT alleles while the sGD^+^ drive would require being paired with a separate vCas9 transgene to produce drive. Moreover, the sGD when paired with Cas9 could transmit NHEJ-inducing maternal Cas9/gRNA complexes to offspring that did not inherit both the Cas9 and gRNA transgenes (75% of progeny), as compared to only 50% for a linked fGD that failed to copy. This differential maternal mutagenic effect is predicted to be further amplified due to the slower intrinsic drive kinetics of the sGD relative to the fGD. Despite the initial dip in fGD frequencies, fGD^+^ alleles increased to a stable plateau level in all cage replicates (G_12_–G_10_: Fig. 2a, purple traces) achieving final introduction frequencies approximating those of the sGD^12^, and the means of their performance curves were highly correlated (*r* = 0.871) despite the divergence in intermediate generations. We sequenced non-converted fGD^−^ individuals that were present at four different timepoints: G_2,_ G_4,_ G_8_, and G_20_ (Fig. 2b) to capture evolution of NHEJ alleles in the population. Sequence data revealed an array of different mutations at G_2_ and G_4_ and also large proportions of unaltered WT alleles (Fig. 2b, black bars). One notable difference between the mutations detected in the fGD versus sGD cages (Supplementary Fig. S3b) was a greater progressive simplification of an initial complex array of NHEJs generated early (G_2_–G_4_) by the fGD compared to the sGD. As mentioned above, the variety of NHEJ alleles decreased over time leveling out by G_8_ and remaining stable through G_20_ (except for an allele represented in gray shading for which an extra 1 bp deletion appeared between G_8_ and that of G_20_). Consistent with the disappearance of convertible WT alleles by G_8_, the fGD^+^ exhibited only a minimal increase in frequency between G_8_ and G_20_.

### Mathematical models extract major drive parameters from population cage dynamics

Building on the aforementioned mathematical models, we extended our analysis to the fGD by assessing two potential fitness cost implementations: (1) an active cost associated with ascending phase of the drive trajectory, where fitness costs due to Cas9/gRNA were only apparent during active cleavage events, and (2) a co-occurrence cost, where the fitness costs manifest whenever an individual has Cas9 and gRNA, regardless of active cleavage. Drive performance was estimated from respective cage trial data using a Naive–Bayes Multi-Objective Hidden Markov Model optimized with an evolutionary algorithm (Supplementary Fig. S4a). Initial parameter estimates for transmission were taken from single-pair mating data (Fig. 1b) and cleavage rates approached 95% (Supplementary Tables S11 and S12), with significantly higher HDR in females (49%) than males (20%). Cleavage and conversion rates correspond to transmission estimates of 73% in females and 60% in males, consistent with single-pair mating observations (Fig. 1b). In addition, we used phenotypical and sequence data from fGD^−^ flies (Fig. 2b) to estimate the fGD^–^ allele frequencies (Supplementary Information File, section Mathematical Data Preparation).

Based on the derived parameters described above, we performed stochastic simulations of our model (see Supplementary Information File, Supplementary Tables S8 and S10, and Supplementary Fig. S5 for sGD and Supplementary Tables S9 and S12, and Supplementary Fig. S7 for fGD) and compared these predicted drive profiles to those obtained experimentally (Fig. 2c and Supplementary Fig. S4b). Stochastic model trajectories were largely consistent with results from the cage trials (Fig. 2c).

### Modeling the relationship between sGD and fGD drive dynamics

Lastly, we assessed the capacity of predicting fGD trends based on sGD performance. To do so, we applied both our models to a range of possibilities in the two main components of the model: conversion rate (CR) and fitness costs (FC) (Supplementary Fig. S5). While 25% increases were assessed for FC, only CRs of 75 and 100% were tested, as gene drives with lesser efficiencies would not be selected as potential candidates. Based on these constraints, a perfect drive would not incur any FC (0%), while the opposite extreme (100% FC) should result in a population that crashes within one generation. Realistically, however, gene-drive costs would range between those two numbers. Our model shows that, while sGD performance does not typically predict fGD well (Supplementary Fig. S5), there is a success threshold (circa 50%) based on the fitness costs that the drive induces on the carrier. We also gathered that simulations for sGDs result in a much wider range of outcomes compared to those on fGDs, mostly due to the randomness of gRNA-Cas9 pairings that occur at every generation and drive the sGD element, as well as the capacity of Cas9 to be selected out of the population (Supplementary Fig. S5, purple vs yellow traces). We conclude that the *spo11* fGD is likely to incur significantly greater fitness costs than the sGD to account for its partial and more delayed drive kinetics relative to predictions based solely on drive parameters derived from the sGD. Moreover, since the frequency of the fGD remained steady over generations, we can infer that this elevated fitness cost is of the first type (active drive: WT allele-dependent) and not a second class (co-occurrence: Cas9+gRNA in the absence of WT alleles).

## Discussion

In this study, we genetically converted a split gene-drive (*spo11* sGD) element targeting a locus (*spo11*) essential for fertility in both sexes into a full gene-drive (*spo11* fGD) and compared these well-matched systems in both single-generation crosses and multigenerational cage experiments. We observed, and mathematically modeled an unexpected delay in drive of the fGD based on simple predictions derived from analysis of sGD systems and a reduced level of final introduction for the fGD that was nearly equal to that of the sGD instead of being higher as predicted by the modeling.

We found that sGD-to-fGD conversion efficiency depends greatly on the proximity between Cas9Hack elements containing Cas9 and target sGD loci. These observations parallel those described previously^14,18,19^, as chromosomal repair or HACK efficiency varied depending on genomic distance and orientation of the target-donor pair. Hi-C distances are particularly relevant in this context, as they estimate the actual physical DNA proximity between two loci within meiotic nuclei. In contrast to this locus specificity for hacking efficiency, chromosomal position of the tested Cas9 sources did not have a major impact on drive performance, although such differences have been noted in other sGD-Cas9 combinations^12,20^. Thus, in future efforts to convert additional sGDs to fGDs, Cas9Hack lines inserted near the genomic integration site of the sGD element in question may prove most efficient. Upon conversion, the size of the copying cargo, with fGD being ~8 kb larger than sGD, may also influence overall conversion efficiencies since modestly reduced copying has been reported for larger drives inserted into the same locus^11,21^.

Although the sGD and fGD performed similarly in single-generation crosses, we observed notable unexpected differences in their performance in multigenerational cage trials. The vfGD spread through a naïve population following initial introduction at low levels to ~80% of the population in 15 generations, but did so with delayed drive kinetics compared to the *spo11* sGD using a static *vasa*-Cas9. Frequencies at G_20_ were comparable for the two systems since they both plateaued after reaching a phenotypic frequency of 75–85% due to the accumulation of mostly non-deleterious resistant NHEJ alleles. Previously, we observed that there was a moderate fitness cost associated with carrying certain sGDs and a Cas9 transgene in the same individual^12^. In contrast to the sGD configuration, where copying of the gRNA-bearing cassette and static Cas9 transgene can separate during transmission (due to independent assortment of the Cas9 transgene), Cas9 is forcibly linked to the gRNA component in the fGD and thus a higher percentage of individuals incur costs associated with co-inheritance of these CRISPR components. This effect is particularly pronounced in early generations when the great majority of non-transgenic individuals will be WT homozygous and thus have fitness advantage over the transgenic line early during the drive process. In the ascending phase of the drive, along with allelic conversion events, WT alleles can be mutated to generate transiently acting dominant mosaic phenotypes^12^. Fitness costs associated with co-inheritance of gRNA and Cas9 transgenes are thus confined to the active drive phase.

Consistent with the above hypothesis, simplification of the NHEJ pattern was observed for fGD compared to sGD, which mostly occurred when the fGD drive stalled between G_3_ and G_7._ By G_8_, WT alleles were no longer detected in fGD cages in contrast to the sGD, where such unmodified alleles persisted for longer. Presumably, Cas9 that is obligately produced by the fGD allele either successfully converted all WT alleles by conversion to the fGD^+^ state or mutated them to cleavage-resistant and largely functional NHEJ alleles, thus resulting in the fGD attaining stable levels of introduction. Despite its initial rise, the fGD did not achieve the predicted levels of introduction most likely due to the accumulation of functional NHEJ alleles. While the fGD generated NHEJ alleles (functional plus nonfunctional) at comparable frequencies to its sGD counterpart (Fig. 1c), as summarized above (and discussed further below), our modeling predicted it would generate fewer residual NHEJ alleles in multigenerational cage trials (Supplementary Fig. S3). Our experimental results differed from this prediction, however, since we observed comparable final levels of introduction and NHEJ allele profiles for the sGD and fGD.

Importantly, as indicated in Supplementary Fig. S5, we note that a full drive is expected to achieve nearly full introduction very rapidly. Indeed, we have observed this predicted behavior for various recoded full drives such as the recoded *kh*-drive in *An. stephensi*^6^, which has a balance of gene conversion versus NHEJ mutagenesis similar to that of the recoded *spo11* fGD in the current study. Similarly, the recoded *prosalpha2* sGD reported previously^12^ drove very rapidly to near completion in the background of 100% Cas9 (a situation similar to that of a full drive in some key respects). Thus, it is a quite an unexpected result that the recoded *spo11* fGD in this study did not perform as modeled based on drive parameters extracted from the sGD in Supplementary Fig. S5. Indeed, we reiterate that full drives differ fundamentally from split drives in that the trajectories of full drives obey second-order recursion equations and thus follow a logistic growth curve (approximated during early phases of the drive by an exponential curve), while split-drive dynamics are described by first-order recursion equations leading only to the additive (or linear) accumulation of the gRNA-bearing element^4^. These fundamental mathematical differences in drive kinetics are well captured by our current model and predict rapid increases in the frequency of the fGD but only gradual asymptotic increases in the sGD. Moreover, as mentioned above, both can produce transgenerational lethal mosaicism even in the absence of inheritance of the CRISPR components. Therefore, the sGD is expected to generate NHEJ alleles more frequently than the fGD when only one or neither of the CRISPR components are inherited by progeny. Moreover, this greater frequency of non-productive maternal transmission of mutagenic Cas9/gRNA complexes for the sGD versus the fGD is further compounded by the inherently slower drive trajectory of the sGD relative to the fGD, which provides yet more opportunities for creating such NHEJ alleles. Thus, an sGD is predicted to generate considerably higher frequencies of NHEJ alleles than the corresponding fGD, which differs from what we observed experimentally (i.e., ~ equal frequencies of NHEJs for both drive systems).

Mathematical modeling confirmed that many of the inferred drive parameters are similar but not identical for the sGD and fGD population cage experiments. Modest deviations between the data and model regarding the degree of final sGD versus fGD introduction may reflect assumptions that the NHEJ allelic profile and the dynamics of their elimination were the same in both fGD and sGD scenarios. However, as mentioned above, in early generations of fGD cages and corresponding to the period of drive delay, nonfunctional NHEJ alleles were simplified and cleared more quickly than for the sGD. Also, WT alleles persisted longer in the sGD cages, perhaps providing less time to winnow deleterious alleles from the population (Fig. 2b). Thus, a notable feature of these experiments and accompanying modeling is that the performance of the *spo11* fGD cannot be simply predicted from drive parameters extracted from the sGD. In particular, modeling suggests that the fGD incurs a substantially greater fitness cost during the drive phase when many wildtype alleles still remain in the population. These costs may be related to accumulating levels of maternal Cas9/gRNA complexes associated with maintaining fGD stocks for multiple generations or may reflect some other feature of the full versus split-drive configurations that pertains to the active drive phase. Note that the fGD remains stable in the population once attaining full introduction and thus does not appear to generate any additional generic co-occurrence fitness costs (i.e., following conversion of all WT alleles into either fGD or NHEJ alleles). Since the fGD increases only gradually over five generations (G_4–9_), one can estimate that the drive-dependent fitness cost must nearly balance the exponential drive of the element during this period. One potential contribution to this fGD lag phase could be yet greater fitness costs incurred by WT individuals inheriting Cas9/gRNA complexes from mothers homozygous for the drive element. Consistent with fGDs providing elevated levels of maternal Cas9/gRNA complexes, fGD produced a higher fraction of nonfunctional frameshift mutations than the sGD, which may reflect differing levels of Cas9 activity. While the theoretical mechanisms behind full and split drives are now fairly well understood, and the dominant interactions properly reflected in the models, higher-order impacts are not captured in these simulations. Detailed as these models are, the discrepancies suggest that yet more fine-scale models may be required to capture biochemical differences in Cas9/gRNA complexes: different categories of fitness costs (active drive versus co-occurrence) in the progeny of Cas9/gRNA-bearing males versus females or parents versus grandparents (or even great-grandparents), DNA accessibility during different developmental stages, and repair mechanisms available when different promoters are active. Modeling and analysis performed in this study, of similar or even greater complexity, is rapidly becoming requisite in the gene-drive toolbox, both for the analysis of experimental data^21,22^ and simulation of potential results to inform conservation^23^ and disease-elimination campaigns^7,24^. In particular, we demonstrate the application of a Naive–Bayes multi-observation HMM that optimizes the regular HMM in a parallel framework by incorporating phenotypic and genotypic data.

In summary, this study provides a direct comparison between split and full-drive configurations in a defined chromosomal locus and validation of a general method for efficiently converting one to the other by genetic crossing. We also provide proof-of-principle for a flexible system to seamlessly convert sGDs into linked fGDs inserted into exactly the same chromosomal location using the same molecular components. Only modest differences in performance were observed between sGD and fGD in single-generation crosses. Given that the fGD is always linked to Cas9, we had expected based on modeling that it should drive more rapidly and completely than the sGD, since the element does not drive when separated from Cas9. Surprisingly, however, we observed the opposite result in multigenerational crosses where fGD drive initially exhibited a transient delay relative to the sGD before achieving comparable levels of final introduction. Our analysis suggests that this unexpected early stalling of the fGD reflects a transiently acting dominant process of sterile mosaicism in which individuals inheriting the drive element and a WT allele or strong sterile phenotypes incur a fitness cost while WT alleles are still present in the population. This temporary penalty resulting from obligate co-inheritance of Cas9 and gRNA transgenes does not prevent the fGD from reaching comparable stable levels of final introgression and vanishes once the drive has attained final levels of introduction and no WT or nonfunctional *spo11* alleles remain.

Both split and full-drive systems offer potential advantages for field applications. The split system provides potential benefits for more localized and controllable spread of the CRISPR components, while full gene drives could be implemented in contexts where long-lasting protection conferred by associated effector genes and conversion of potential incoming WT alleles may be desired. The efficient and versatile hacking strategy we describe here can be employed to optimize the performance of key components in split configurations and then convert such systems into full drives. An important lesson of this study, however, is that the final performance of the fGD needs to be empirically determined, as biochemical differences limit the predictive value of sGD models applied to an fGD linked configuration. The seamless mobilization of Cas9 into the sGD provides a range of experimental advantages, in particular for species where transgenesis is extremely laborious and time intensive.

## Methods

### Ethical conduct of research

We have complied with all relevant ethical regulations for animal testing and research and conformed to the UCSD Biosafety committee-approved biological use authorization.

### Plasmid construction

All plasmids were cloned using standard recombinant DNA techniques. Plasmid and genomic DNA sequences were amplified using Q5 Hotstart Master Mix (New England Biolabs, Cat. #M0494S) and Gibson assembled with NEBuilder HiFi DNA Assembly Master Mix (New England Biolabs, Cat. # E2621). The resulting plasmids were transformed into NEB DH5-alpha chemically competent *E. coli* (New England Biolabs, Cat. # C2987), isolated and sequenced. Primer sequences used for the creation of the different Cas9Hack plasmids can be found in the Supplementary File. *spo11* and *rab5* sGD plasmid construction has been described previously^12^. Recoded cDNA fragments were designed by using non-sub-optimal alternative codons from CRISPR cut site to gene’s stop codon and synthesized as gBlocks™ (Integrated DNA Technologies). Codon usage frequencies were kept as similar as possible to that of the endogenous sequence.

### Microinjection of constructs

Plasmids were purified using the PureLink Fast Low-endotoxin Maxi Plasmid Purification kit (ThermoFisher Scientific, Cat. #A35895). All plasmids were sequenced prior to injection. Embryo injections were carried out at Rainbow Transgenic Flies, Inc. (http://www.rainbowgene.com). Each Cas9Hack construct was injected into AttP-harboring lines expressing integrase in the X chromosome (Bloomington #R8621 and #R8622). Injected embryos were received as G_0_ larvae, and allowed to emerge and 3–4 females were intercrossed to 3–4 males. G_1_ progeny were screened for positive transgene marker (green body). All transgenic flies that displayed the marker were then balanced using Sco/CyO (for Hack-A, located on the II chromosome) or TM3/TM6 (Hack-B, III chromosome) and kept on a *w*^1118^ background. Homozygous stocks were maintained in absence of any balancer alleles or markers associated to the initial inserted line. Correct and complete transgene insertions in homozygous stocks were validated through PCR amplification and Sanger sequencing.

### Fly genetics and crosses

Fly stocks were maintained on regular cornmeal medium under standard conditions at 20–22 °C with a 12-h day–night cycle. sGD and Cas9Hack stocks were kept separate in glass vials in an ACL-1 fly room, freezing the flies for 48 h prior to their discard. We assessed Cas9Hack lines ability to drive by genetically crossing the CC*vasa* source to each Cas9Hack line. To do so, individual trans-heterozygote F_1_ males or virgin females were collected for each G_0_ cross and crossed to a WT fly of the opposite gender. Single-generation crosses were grown at 25 °C. Inheritance of both gRNA (>50%) and Cas9 (~50%) were calculated using the resulting F_2_ progeny by scoring the phenotypic markers associated to each transgenic cassette. Hacking efficiency experiments were performed by crossing sGD/Cas9 heterozygotes to the opposite gender carrying a balancer and detecting their progeny displaying double-marked phenotype over that balancer (Supplementary Fig. S1a). For sGD-Cas9 combinations that were present in the same chromosome, heterozygote males were preferable due to their lack of genetic recombination. All experiments performed after successful Cas9 introduction into the *spo11* sGD locus, *spo11* fGD lines were maintained in plastic vials in a contained ACL-2 insectary dedicated to *Drosophila* gene-drive research. Used vials or samples were frozen for at least 48 h prior to their removal from the facility to be discarded or worked with, respectively.

### Multigenerational cage trials

All population cage experiments were conducted at 25 °C with a 12-h day–night cycle using 250 ml bottles containing standard cornmeal medium. Crosses between homozygous fGD^+^ and wildtype (fGD^−^) flies were carried out to obtain F_1_ fGD^+^/fGD^−^ heterozygotes, which were used to seed the initial generation. Wildtype or heterozygous males and virgin females were collected and separately matured for 3–5 days. Cages were seeded at a phenotypic frequency of 25% fGD^+^ heterozygotes (15 males, 15 females) to 75% fGD^−^ (45 males, 45 females). Upon transfer into fresh bottles for each generation, flies were allowed to mate and lay eggs for 3 days, then were removed from the cage (G_n_) and bottles were kept for 10 days to allow for the subsequent generation to develop to adulthood. Adult progeny (G_n+1_) was randomly separated into two pools and scored; one pool collected for sequencing analyses while the other was used to seed the following generation. If the two pools differed much phenotypically, frequencies were averaged in order to reduce variability and stochastic extremes. Continuous sampling and passage were carried out for 20 generations.

### Molecular analysis of resistant alleles

To extract fly genomic DNA for single fly resistant allele sequence analysis, single flies were squashed in lysis solution (10 mM Tris-Cl pH 8.2, 1 mM EDTA, 25 mM NaCl and 0.2 mg/ml proteinase K), incubated at 37 °C for 30 min and deactivated at 95 °C for 2 min. After extraction, each sample was diluted in water and stored at −20 °C if needed. 1–2 µl of each diluted DNA extraction was used as a template for a 25 µl PCR reaction that covered the flanking regions of the gRNA cut site, which were used to sequence the alleles. Sanger sequencing in individual non-fluorescent flies was performed at Genewiz, Inc. in San Diego, CA to obtain the single-cross NHEJ data. NHEJ allele sequences were obtained from Sanger chromatographs by isolating the WT sequence first and then annotating the remaining allelic sequence. For cage trials, 20–25 non-fluorescent flies were pooled and their DNA extracted at each sampling generation (G_2_, G_4_, G_8_, and G_20_). Target sequences were amplified using specific gene primers that also contained adapter sequences. Non-fragmented amplicons were sequenced using Illumina-based technology (2x250bp reads, Amplicon-EZ, Genewiz), with final data being delivered as FASTQ reads and aligned to a reference sequence for each gene of interest to detect indel formation. Primer sequences used for either single fly or cage trial deep-sequencing analyses can be found in the Supplementary File.

### Mathematical modeling

Model fitting was performed using a discrete-generation adaptation of the Mosquito Gene Drive Explorer (MGDrivE)^25^ software. A multi-objective HMM, using the log-likelihood as a score function, was optimized by the evolutionary algorithm and used to generate parameter estimates with corresponding 95% quantiles. Mendelian inheritance was assumed except under co-occurrence of the Cas9 and gRNA constructs when the split drive (and HACK) design allowed active cleavage of the target chromosome and the possibility of super-Mendelian inheritance. When cleavage occurred, a fraction of the cut alleles could be properly repaired via HDR, and the remaining cut alleles underwent NHEJ repair, generating *in* or *out-of-frame* resistant alleles. The effects of shadow-drive^6,17^, in which Cas9 protein is deposited in the embryo of a female individual who does not carry the Cas9 allele, but whose own mother does, were also accommodated. Fitness costs were implemented as fractional reductions in male and female fecundity, consistent with *spo11* activity, and tested under active cleavage conditions as well as co-occurrence of Cas9 and gRNAs. Additional details on the model implementation and likelihood function used in sGD and fGD model fitting can be found in the Supplementary Information. Phenotype and genotype mappings are provided in Supplementary Tables S2–7. Fitness cost implementations, active vs co-occurrence, are shown in Supplementary Tables S8 and S9. Parameter descriptions, estimates, and 95% quantiles are provided in Supplementary Tables S10–13. The resulting fits are visualized, with cage trial data, in Supplementary Figs. S5–8. Supplementary Figs. S9 and S10 analyze parameter estimate correlations, while Supplementary Figs. S11 and S12 are partial rank correlation coefficient (PRCC) analyses.

Simulated model trajectories for Fig. 2c were generated using a stochastic implementation of the discrete-generation model. At each generation, adult females mate with males, thereby obtaining a composite mated genotype (their own, and that of their mate) with mate choice following a multinomial distribution determined by adult male genotype frequencies, modified by mating efficacy. Egg production by mated adult females then follows a Poisson distribution, proportional to the genotype-specific lifetime fecundity of the adult female. The offspring genotype follows a multinomial distribution informed by the composite mated female genotype and the inheritance pattern of the gene-drive system. Sex distribution of offspring follows a binomial distribution, assuming equal probability for each sex. Female and male adults from each generation are then sampled equally to seed the next generation, with sample size proportional to the average size of the cage trials at that generation, following a multivariate hypergeometric distribution. All simulations were performed and analyzed in R^26^.

### Figure generation and statistical analysis

Initial graphs were generated using Prism 9 (v9.2, GraphPad Software Inc., San Diego, CA) and modified using Adobe Illustrator (v25.4.1, Adobe Inc., San Jose, CA) to visually fit the rest of the non-data figures featured in the paper. Supplementary Figs. 2 and S4 contain parts that were generated using BioRender.

### Safety measures

All research involving non-hackable gRNA cassettes/split gene drives was performed in glass vials in an ACL-1 facility, whereas research involving full gene drives (*spo11* fGD lines) was conducted in plastic disposable vials in an ACL-2 facility, in accordance with the Institutional Biosafety Committee-approved protocol from the University of California San Diego. All vials were frozen for 48 h prior to autoclaving and discarding the flies. ACL-2 samples were also frozen for at least 48 h before their removal from the contained facility.

### Reporting summary

Further information on research design is available in the Nature Portfolio Reporting Summary linked to this article.

## Supplementary information

Supplementary Information File

Reporting Summary

## Data Availability

Primers used for plasmid construction and sequencing (single and deep-sequencing experiments) can be found in Supplementary Table S1. Full plasmid sequences can be found at the end of the manuscript’s Supplementary Information File. All data generated in this study, and processed data, are provided in the Supplementary Information/Source Data file. Modeling information can be found in the Supplementary Information file. Source data are provided with this paper.

## Source data

Source Data

## Code availability

A version of MGDrivE was used for simulation modeling and is freely available from the MGDrive GitHub repository (https://marshalllab.github.io/MGDrivE/) as well as uploaded in Zenodo (10.5281/zenodo.7312471). Specific codes can be obtained from the authors upon request.
