# Supplementary material for: Genetic conversion of a split-drive into a full-drive element
Source: Nat Commun. 2023 Jan 12;14:191. doi: 10.1038/s41467-022-35044-4 (PMC9837192; doi:10.1038/s41467-022-35044-4)
Supplement: Supplementary file 1 — Supplementary Information File [file 41467_2022_35044_MOESM1_ESM.pdf]

Genetic conversion of a split-drive into a full-drive element

Gerard Terradas[1,2,6], Jared B Bennett[3], Zhiqian Li[1,2], John M Marshall[4,5], Ethan Bier[1,2*]

[1] Department of Cell and Developmental Biology, University of California, San Diego, La Jolla, CA 92093, USA

[2] Tata Institute for Genetics and Society, University of California, San Diego, La Jolla, CA 92093, USA

[3] Biophysics Graduate Group, Division of Biological Sciences, College of Letters and Science, University of California, Berkeley, CA 94720, USA

[4] Divisions of Epidemiology and Biostatistics, School of Public Health, University of California, Berkeley, CA 94720, USA

[5] Innovative Genomics Institute, Berkeley, CA 94720, USA

[6] Current address: Department of Entomology, The Center for Infectious Disease Dynamics, and the Huck Institutes for the Life Sciences, The Pennsylvania State University, University Park, PA 16801, USA

* Corresponding author

e-mail: ebier@ucsd.edu

Key words: split drive, full drive, *Drosophila*, Cas9, CRISPR, hacking, gene drive

This Supplementary Information file contains:

1. **Main Supplementary Figures** (Figures S1-S4)
2. **Main Supplementary Table** (Table S1)
3. **Mathematical Supplementary** (Tables S2-S13 and Figures S5-S12)
4. **Full plasmid sequences**

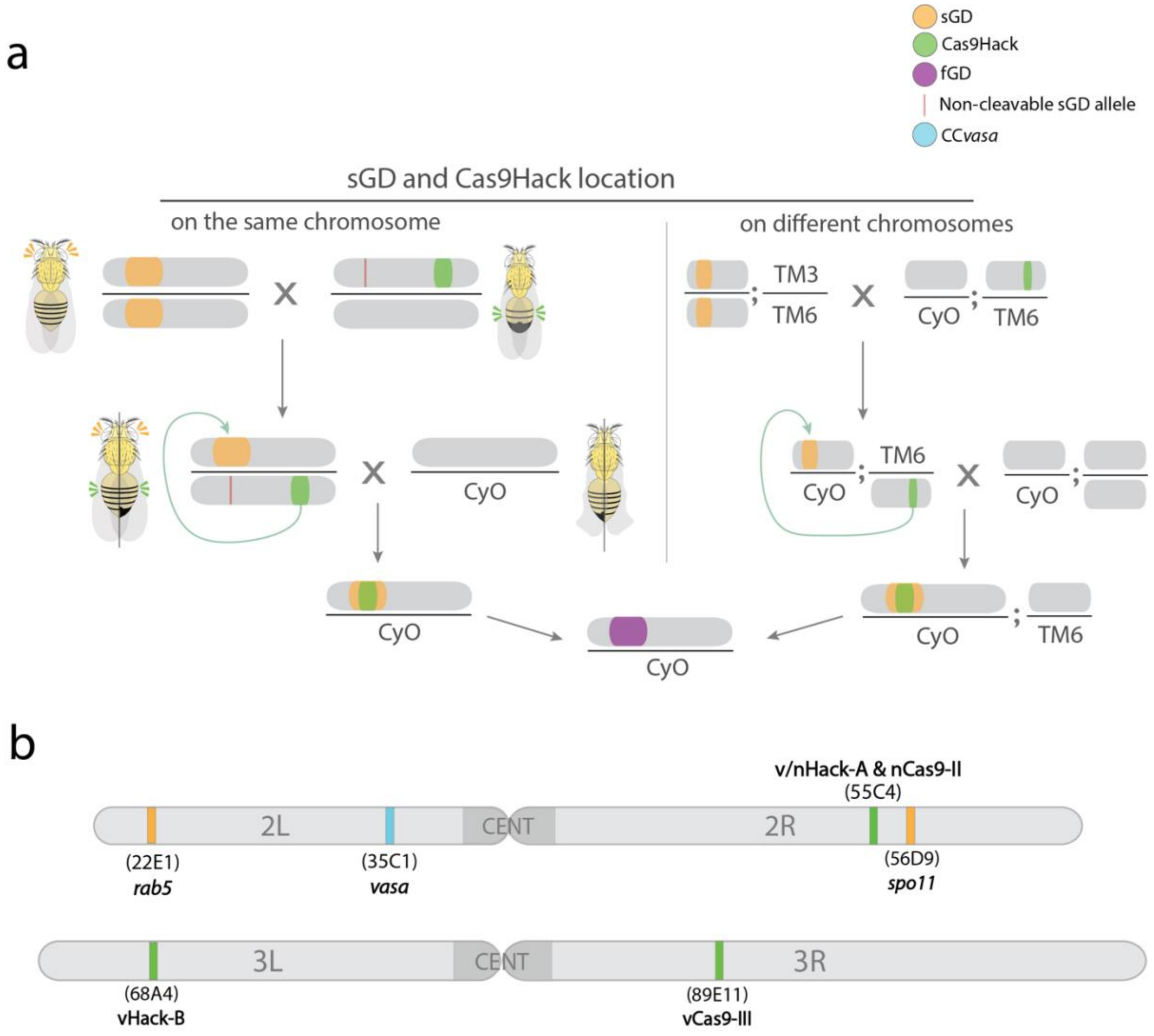

Figure S1 – Experimental design of the split to full gene drive conversion.
a) Outline of the genetic cross schemes used to convert a split system into a full gene drive. **b)** Chromosomal location of the sGDs tested for conversion and hacking-Cas9 transgenes in the *Drosophila melanogaster* genome.

Figure S2: introduction

Homozygous G_0 virgin CC*vasa* females were mated to Cas9Hack males to obtain flies trans-heterozygous for both elements. Single F_1 heterozygote males or virgin females were then crossed to wildtype flies (WT) of the opposite gender and their F_2 progeny were scored for presence of the blue and green fluorescent markers carried by the CC*vasa* and Cas9Hack elements, respectively (Figure 2a). When allelic conversion occurred in females, both traditional Cas9 lines performed comparably (vCas9-III=78±11%; nCas9-II=77±5%). Similar transmission values were also observed for Hack-A lines (vHack-A=78±9%; nHack-A =82±10%), which did not differ significantly from the control rates of vCas9-III and nCas9-II-mediated copying (two-tailed Mann-Whitney test for *vasa*: U=494.5, p=0.99; *nos*: U=170.5, p=0.07). In males, conversion frequencies were lower but again no significant differences were observed between Cas9 sources (vCas9-III=68±8%, nCas9-II=69±8%, vHack-A=67±10%, nHack-A=68±10%) (two-tailed Mann-Whitney test for *vasa*: U=218, p=0.45; *nos*: U=159.5, p=0.30).

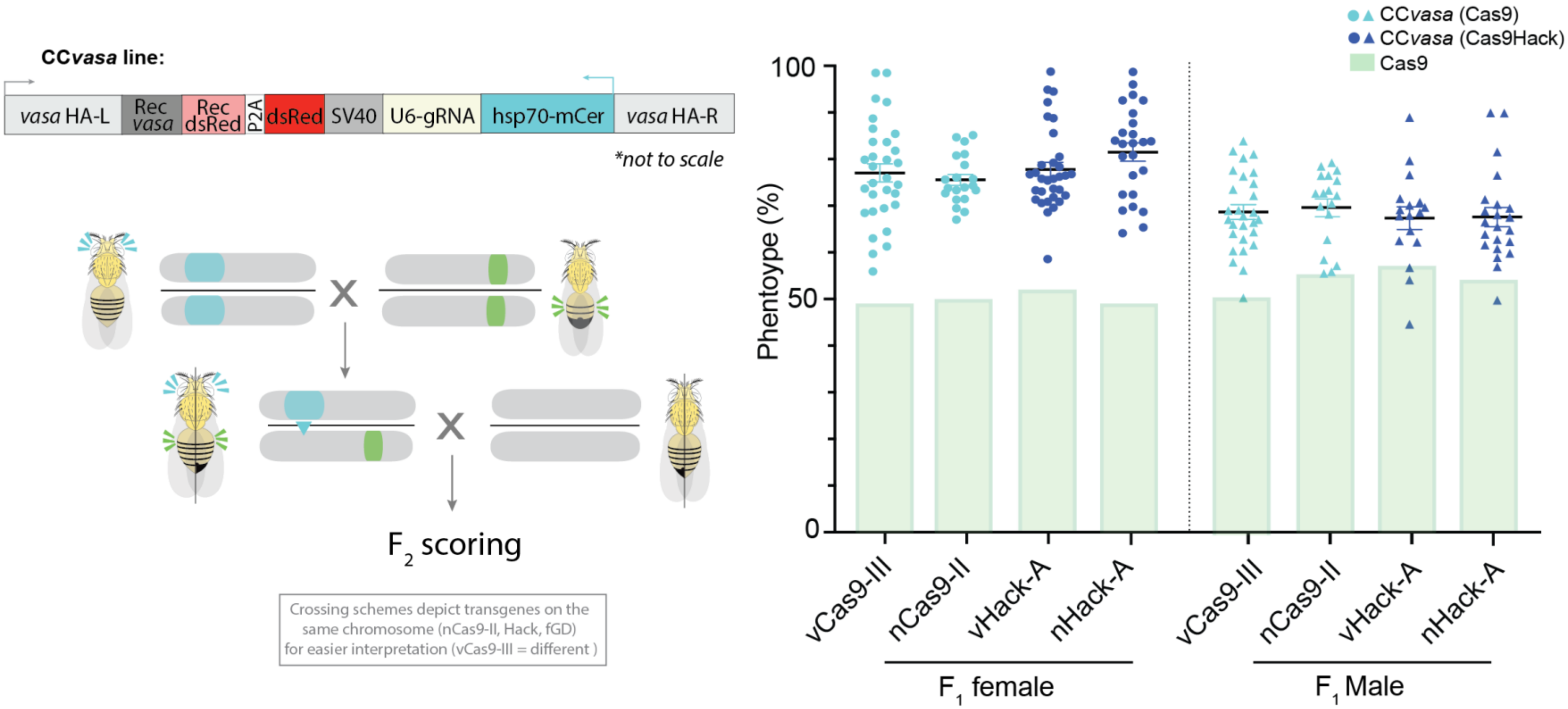

Figure S2 – Super-Mendelian performance is comparable between split and full gene-drive elements in single-generation crosses. Performance of hacking *vasa* and *nos*-driven Cas9 was tested and compared to previously used static Cas9 lines (all marked with EGFP) by crossing them to an unhackable split drive element (CC*vasa*) inserted in the endogenous *vasa* locus, marked with mCerulean. Single F_1 germline conversion was assessed by scoring the markers for both transgenes in the F_2 progeny. Independent inheritance of Cas9 and CC*vasa* is depicted using green bars and blue dots, respectively. Sex of the parental (F_1) trans-heterozygote is indicated in the X-axis under the Cas9 line used, as well as by circles (female) or triangles (male), used to show the data of each individual cross. Error bars represent mean values ± SEM. Raw phenotypical data is provided in the Supplementary Data File.

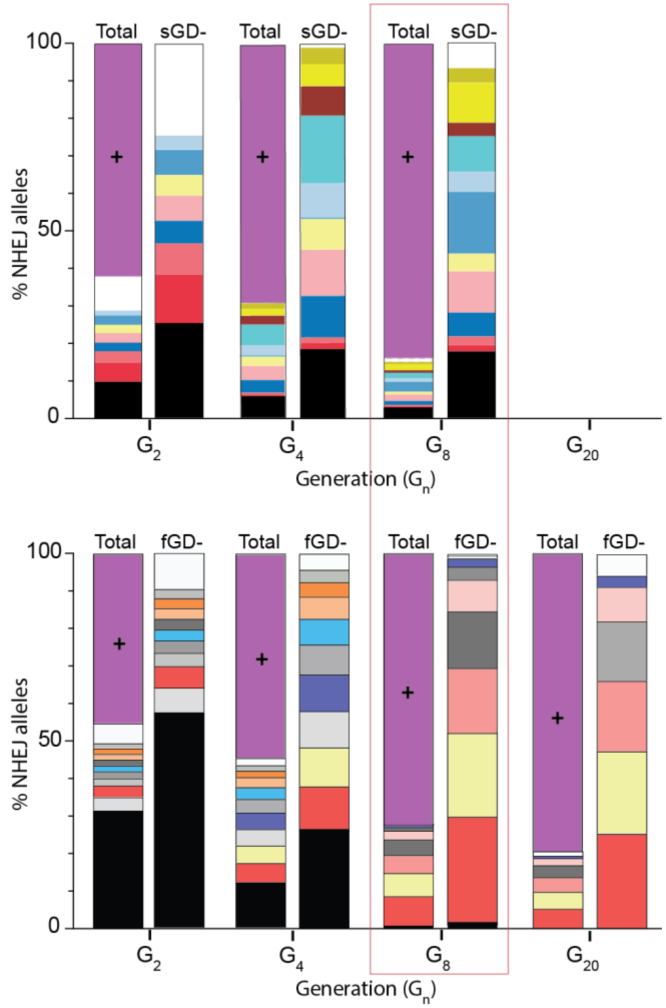

Figure S3 – **Comparison of cage trial sGD (top) and fGD (bottom) NHEJ generation patterns.** NHEJ cage trial data was obtained by deep-sequencing the target site region of pooled non-fluorescent individuals at specific generations. At each generation, NHEJ alleles are shown to represent their distribution among the total population (left) or only for non-fluorescent flies (right). Purple bars show the sGD^+ or fGD^+ population percentage. To note that, following a similar pattern in G_4, some mutations get selected for at G_8 on fGD cages compared to sGD's, which present a higher variation of NHEJ events.

Figure S4: introduction

We used fitness costs from the previous study [1], but with wider ranges to account for possible changes in fitness (see Mathematical Supplementary for a complete description of inheritance implementation, HMM, likelihood function, and estimated parameters). Cleavage rates approached 95% (95% quantiles: 90-95%, Tables S11 and S12), with significantly higher HDR in females (49%, 95% quantiles: 40-58%) than males (20%, 95% quantiles: 20-37%). Cleavage and conversion rates correspond to transmission estimates of 73% in females (95% quantiles: 68-78%) and 60% in males (95% quantiles: 59-68%), consistent with single-pair mating observations (Figure 1b). Rates of *in-frame* vs *out-of-frame* NHEJ events varied widely, but preferentially towards higher *in-frame* rates, consistent with our previous work and the importance of *spo11* for fertility. However, the models lean towards no fitness cost from Cas9 and gRNAs, under either the active or co-occurrence models, which does not correlate with what was observed for the *spo11* transgene acting as sGD. This limitation of the modeling can be explained by virtue of the hacked design; the initially separated fluorescent markers from the sGD and Cas9 constructs are now linked in a single cassette (fGD), effectively presenting a single fluorescent marker than can only distinguish GD^+ versus GD^- phenotypes. Thus, we applied the estimated fitness costs of Cas9 and gRNAs for the *spo11* transgene as sGD and obtained similar parameter estimates. When considering the modest amount of sequencing data relative to the phenotypic assessments (~80 flies sequenced, compared to 19,386 flies counted for phenotype data), our models are heuristically equivalent to the measured allele frequencies with differences only minimally impacting our parameter estimates. The stochastic model captured the potential role of chance events such as mate choice (multinomial-distributed), egg production (Poisson), progeny genotype (multinomial), and the finite sampling of the next generation (multivariate hypergeometric).

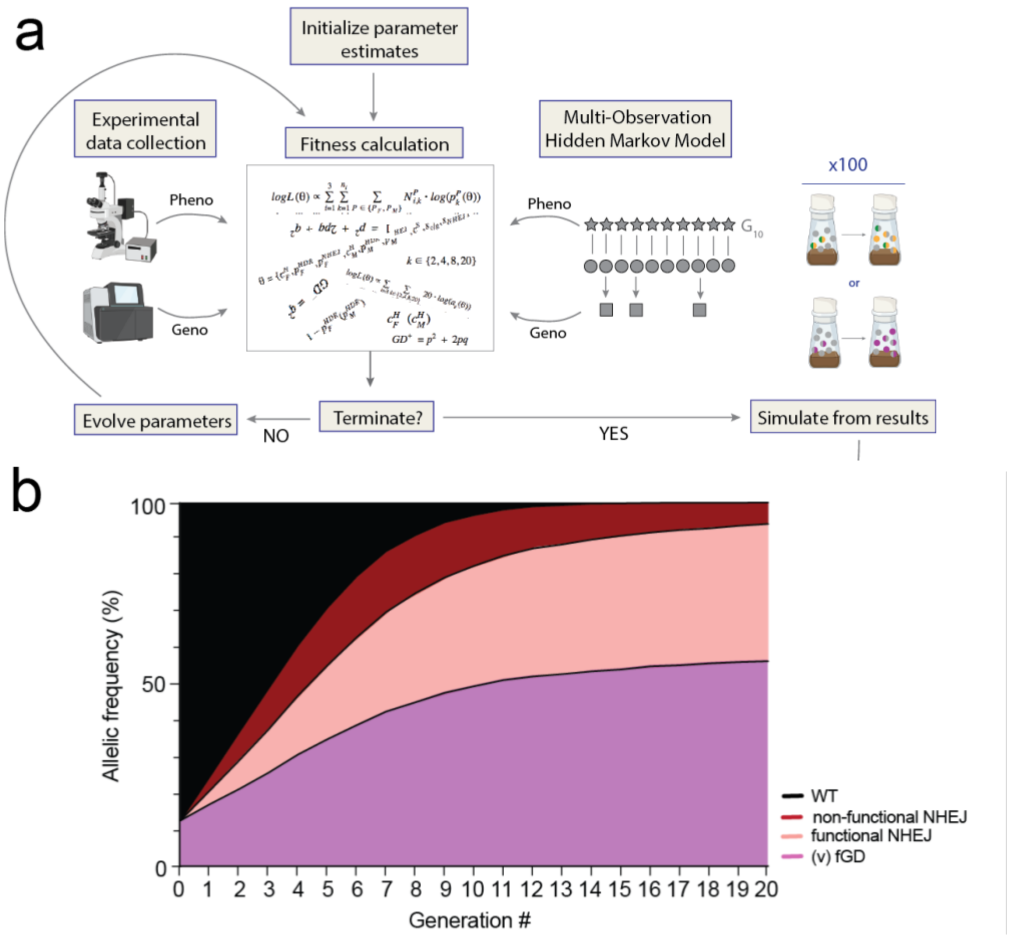

Figure S4 – Mathematical model simulations provide insights in dynamics.
a) Schematic of the workflow to obtain accurate mathematical models for *spo11*'s sGD and fGD configurations and their associated fitness parameters. **b)** Predictions of alleles present in the fGD cage simulations at each generation. Purple, red shades and black curves depict fGD^{+}, different types of NHEJ and WT allele, respectively.

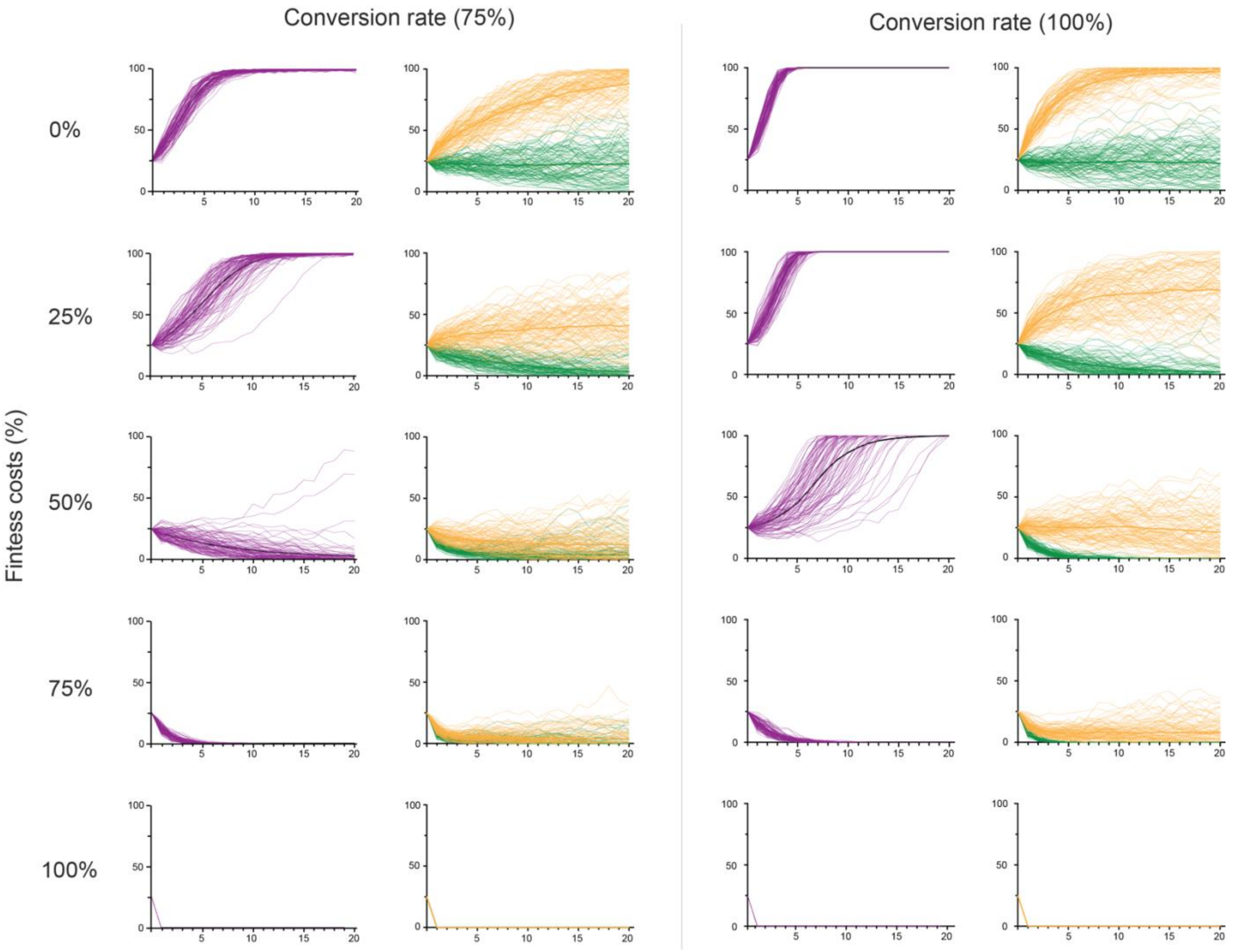

Figure S5 – Conversion rate and fitness cost dependency of fGD and sGD mathematical models.

Variations in conversion rate and fitness costs greatly determine fGD (purple) performance in cage simulations, as well as sGD (yellow) and Cas9 (green). Synthetic analysis highlights the performance differences due to construct design. These should be expected behavioral deviations in cage trials, as they are due to the design, with biological variation possible in addition. The MGDriveE modeling software [25] we employ here captures the key features of ideal fGD versus sGD drives, for which closed-form mathematical solutions

exist. Thus, an fGD drive trajectory is predicted to follow a logistic growth curve defined by the second-order recursion formula: $f_{n+1} = f_n + f_n(1 - f_n) = 2f_n - f_n^2$, where f_n is the frequency of the gene-drive in the population at generation n. This formula has the closed form solution $f(n) = 1 - (1 - c_0)^{(2^n)}$, where c_0 is the seeding frequency of the gene drive [4]. For low values of c_0, f(n) can be approximated by the exponential equation $f(n) = c_0 2^n$. In contrast to the predicted exponential performance of the fGD, the sGD drive can be modeled by the first order recursion formula: $f_n = f_{n-1} + c_0(1-f_{n-1})$ where $c_0 = g_0$ (initial fractions of cas9 and gRNAs in the population). The closed form solution for this equation is $f(n) = 1- (1- c_0)^n$, which for low values of $c_0 = g_0$ can be approximated by the linear equation $f(n) = c_0(n)$ [4].

Table S1 – **Primer table.** Table containing the primer sequences used for the creation of the Cas9Hack plasmids (yellow shade), as well as primer sequences used in Sanger sequencing to detect *de novo* NHEJ alleles and for deep-sequencing of the *spo11* locus in multigenerational cage trials (orange shade). Bold nucleotides of each primer sequence depict perfect homology to the original vector, with non-bolded nucleotides showing the overhangs for Gibson assembly. For sequencing primers, Illumina adapters are shown in non-bolded italicized nucleotides.

	Primer	SEQUENCE
**Cas9Hack**	HA1_outBB	GAACCGGCTTCTTG**GTCGACATGCCCGCCGTG**
	HA1_Fw	CATGTCGAC**CAAGAAGCCGGTTCAGCTGC**
	HA1_Rv	**GACCGGCTGCCAGATTTTCAA**
	vasa_in	TTGAAAATCTGGCAGCCGGTC**CTGCAGCTGGTTGTAGGTGC**
	vasa 3'_R	CAAACTCATCAATGTATCTTAAAGCTT**AACACGAAGAGCAGCAGTGTG**
	nos_in	TTGAAAATCTGGCAGCCGGTCT**GCCGCGCCGATTTCAGGG**
	nos 3'_R	CAAACTCATCAATGTATCTTAA**AGCTTCGATGGCATCTTCCTGGCCC**
	SV40_Fw	**AAGCTTTAAGATACATTGATGAGTTTGGACA**
	gRNAHack_Rv	GTCACTGCACGCCGTAGGTCGAC**GTTAAATTGAAAATAGGTCTATATATACG**
	gRNAHack_FwtoBB	GTCGACCTACGGCGTGCAGTGAC**GTTTTAGAGCTAGAAATAGC**
	**Experiment**	**SEQUENCE**
***spo11* sequencing**	Single crosses	**CCTCGAGTTGCTGAGCAACTTG**
	Cage trials	*ACACTCTTTCCCTACACGACGCTCTTCCGATCT***GCGTGCTGATTTACATGCTCTC**
		*GACTGGAGTTCAGACGTGTGCTCTTCCGATCT***GGCAATGTCAGAGGTCCACC**

Mathematical Supplementary

Inheritance Design:

Split drive (sGD)

We model two drives in this experiment: a split drive and a HACK drive, which behaves as a full-drive system, same as the basic MCR design. The split-drive system is implemented on two autosomes; Cas9 on chromosome III, labeled with EGFP, and the gRNAs on chromosome II, labeled with tdTom. To handle the effects of shadow drive (in which Cas9 is deposited into the egg of a mother who doesn't carry the Cas9 allele, but whose own mother does), we implement three alleles at the Cas9 locus: W, the wild-type allele, C, the Cas9 (marked with EGFP) allele, and S, the shadow drive allele (for females who have the W allele, but whose mothers have the C allele). At the gRNA locus, as homing occurs here, we implement four alleles: W, the wild-type allele, G, the gRNA (marked with tdTom) allele, and R/B, which are functional/non-functional resistance alleles. These alleles, and their corresponding marker phenotypes, are summarized in Table S2. As flies are diploid, this allele scheme implies five unique and viable genotypes at locus 1 and ten genotypes at locus 2. Since the two loci assort independently, this means we have 50 possible genotypes, which based on the fluorescent labels, fall into four phenotypic categories. The resulting genotype-phenotype mappings are summarized in Table S4.

Given the large number of possible mating pairs, it is not feasible to show the complete equations for the next generation genotype frequencies, so we instead define a set of rules that describe offspring genotype frequencies for all mating classes, and explain their application using examples for each mating class. While all crosses accept sex-specific parameters, the general equations are agnostic to sex.

The simplest inheritance scenarios are those where there is no gene drive involved. As this is a split-drive system, both the Cas9 and gRNA alleles must be present for homing to occur. Individuals without at least one copy of each of the Cas9 and gRNA alleles therefore abide by Mendelian inheritance rules. The only exception to this is when shadow drive occurs, a phenomenon whereby female offspring of mothers with Cas9 receive a certain amount of Cas9

protein deposited during oogenesis, but no Cas9 allele. In the presence of this deposition, along with a copy of the gRNAs, a limited amount of homing is possible. Due to the rareness of this event and the size of the cage trials, it was not possible to estimate the ratio of accurate homology-directed repair (HDR) versus resistance allele formation as a result of this phenomenon. Additionally, single-pair crosses indicate a high frequency of uncleaved wild-type alleles under these conditions, indicating very high HDR rates but potentially minimal cutting rates. Therefore, we estimated a shadow drive-mediated cleavage rate, c^S, such that the remainder of alleles, $1 - c^S$, remain wild-type. We assume that all alleles cleaved through the shadow drive mechanism are repaired through accurate HDR. This parameter applies to females only, as experiments indicate no shadow drive effect in males, potentially due to the reduced protein load within sperm.

When at least one of each of the Cas9 and gRNA alleles are present in an individual, the pieces combine to promote Cas9-mediated cleavage and repair. We do not fit copy-number dependent parameters in this analysis, therefore having one Cas9 allele provides the same cleavage rate as two copies. In the presence of both Cas9 and gRNA alleles, a fraction, c_F^H (c_M^H), of wild-type alleles are cleaved in females (males), while the remaining $1 - c_F^H$ $(1 - c_M^H)$ remain wild-type in females (males). Given cleavage, accurate HDR occurs at a rate p_F^{HDR} (p_M^{HDR}), while the remaining $1 - p_F^{HDR}(p_M^{HDR})$ alleles undergoing some form of non-homologous end-joining (NHEJ) event. For alleles that are cleaved, but do not undergo HDR, a fraction p_F^{NHEJ} (p_M^{NHEJ}) are repaired in-frame, creating functional alleles, while the remaining $1 - p_F^{NHEJ}$ $(1 - p_M^{NHEJ})$ are repaired out-of-frame, generating nonsense mutations and creating non-functional alleles. The parameters for this design are listed in Tables S10 and S11, including a short explanation of each parameter and maximum likelihood parameter estimates from model fitting.

HACK: full drive (fGD)

HACK combines the Cas9 cassette with the gRNA cassette on chromosome II. The combined construct is still labeled with EGFP for the Cas9 and tdTom for the gRNAs. As the Cas9 and gRNAs are one construct now, we designate it as the homing allele, H. The wild-type allele is again marked with W, while functional/non-functional NHEJ alleles are designated R and B, respectively. These four alleles create 10 unique diploid genotypes, summarized in Table S3. However, since the fluorescent markers are linked, there are only two unique phenotypes, summarized in Table S5.

While there are significantly fewer mating pairs in the HACK design than the split-drive design above (10 unique diploid genotypes), we describe mating and allele transmission in a similar manner. While Cas9 and gRNA always occur together, when absent or across from an NHEJ allele, inheritance follows the Mendelian pattern, with an exception for maternal deposition. Maternal deposition occurs when the mother has Cas9/gRNA, and loads the eggs with pre-formed complexes, that then target and cleave the male allele during zygogenesis. Due to the rareness of this event, the small size of the cage trials, and the paucity of phenotypic reporters, we were unable to estimate HDR or NHEJ rates from this event. We assume that all cleavage events are repaired using HDR with the allele present, and fit a cleavage rate, c^D, such that the remainder of alleles, $1 - c^D$, remain wild-type. We only interrogate this parameter for females, as there is no indication of paternal deposition.

Cas9-mediated cleavage and repair are possible when Cas9/gRNA occur heterozygous with a wild-type allele (denoted W above). In this situation, a fraction, c_F^H (c_M^H), of wild-type alleles are cleaved in females (males), while the remaining $1 - c_F^H$ $(1 - c_M^H)$remain wild-type in females (males). Given cleavage, accurate HDR occurs at a rate p_F^{HDR} (p_M^{HDR}), while the remaining $1 - p_F^{HDR}(p_M^{HDR})$ alleles undergoing some form of non-homologous end-joining (NHEJ). For alleles that are cleaved, but do not undergo HDR, a fraction p_F^{NHEJ} (p_M^{NHEJ}) are repaired in-frame, creating functional alleles, while the remaining $1 - p_F^{NHEJ}$ $(1 - p_M^{NHEJ})$ are repaired out-of-frame, generating nonsense mutations and creating non-functional alleles. The parameters for

this design are listed in Tables S12 and S13, including a short explanation of each parameter and maximum likelihood parameter estimates from model fitting.

Fitness Costs Implementation:

In addition to parameters determining inheritance bias, the gene-drive system is associated with fitness costs through multiple mechanisms: i) possession of a functional Cas9 element in conjunction with a viable gRNA construct, and ii) NHEJ events at the gRNA locus. We disregard assortative mating issues here because all experiments were performed in a *white*$^-$ background. It was hypothesized that costs from Cas9 or gRNA alone were insignificant compared to costs from Cas9 and gRNA co-occurring. However, early model fits did not support this hypothesis in split-drive, so an additional cost for expression of Cas9 without gRNAs was implemented for split-drive. Possession of an active Cas9/gRNA complex was modeled as leading to reduced fecundity (in females) and reduced mating competitiveness (in males), denoted in both cases by the fitness cost, $s_{c/g}$. The cost was implemented in a copy-number independent fashion, so an individual possessing two copies of Cas9 experienced the same cost as an individual possessing one copy of Cas9. However, previous model fits did not explore whether the fitness cost applied whenever Cas9/gRNA co-occurred or only when active cleavage/homing occurred. Therefore, we implemented both scenarios, and looked for a difference between those scenarios. Additionally, the Cas9-expression cost was applied regardless of gRNA status in a split drive, denoted s_{cas9}, in the same fashion as $s_{c/g}$- impacting female fecundity, male mating competitiveness, and independent of copy-number. Genotypes and the costs applied to them are shown in Tables S8 and S9.

Fitness reductions due to loss-of-function (LOF) mutations (B alleles, non-functional NHEJ events) manifest differently from Cas9/gRNA costs. *Spo11* impacts female fecundity, and so females homozygous for LOF alleles lay no eggs, while males are unaffected. Therefore, we explored a fitness cost, s_{NHEJ}, associated with "heterozygous" LOF allele carriers. We quote "heterozygous" here because heterozygotes are viable and fit (the LOF mutations are recessive in fitness impact),

so what this parameter really estimates is the fitness consequence of a degree of somatic mosaicism. The extent and bodily location of somatic mosaicism greatly impacts the fecundity reduction due to the LOF mutations, and is itself dependent on the genomic location of each construct.

Additionally, it was found that females homozygous for *spo11* LOF are mostly infertile but not 100% infertile. In light of this, we implemented another fitness reduction due to being a compound homozygote for LOF NHEJ alleles, s_{NHEJ^2}.

Fitness is defined relative to wild-type organisms. As we apply several fitness costs additively, we have to ensure that this results in a cumulative fitness cost between 0 and 1. We achieved this by truncating the total cost for an individual at 100%. Thus, if an individual has one Cas9 allele (in addition to at least one gRNA allele) and one LOF allele, their fitness cost is $min((s_{c/g} + s_{NHEJ}),1)$. This is intuitive biologically, as having two costly alleles implies that the organism experiences the fitness impact of both; however, it ignores any synergistic or nonlinear effects resulting from combining several costs in one organism. Implementation of the fitness costs is described in Table S8 (split drive) and Table S9 (HACK). Short descriptions of each parameter, along with maximum likelihood estimates, can be found in Tables S10-S13.

Data Preparation:

The phenotype data was prepared as described in the main methods and directly used.

Bulk sequencing data for the HACK cages required further estimation. Only phenotypically negative flies without the drive system (therefore non-EGFP/non-tdTom) were sequenced. This means that we only had partial allele frequencies. However, if we assume Hardy-Weinberg equilibrium, then we can relate allele frequencies using equation 1 and genotype frequencies using equation 2.

(1) $1 = p + q$

(2) $1 = p^2 + 2pq + q^2$

Additionally, as the fluorescent markers are dominant, we know which genotypes are scored as GD+ or GD-

(3) $GD^{+} = p^2 + 2pq$

(4) $GD^{-} = q^2$

Since we only sequenced the phenotypically negative flies, GD^{-}*, we can combine WT and NHEJ frequencies to calculate the minor allele frequency using equation 4. From there, we can use equation 1 or 2 to solve for the major allele frequency. This provides an estimated but complete allele frequency spectra.*

The caveat with this procedure is the reliance on Hardy-Weinberg assumptions, namely random mating, no selection, and a large population size. We know that the population sizes are not large, and we expect fitness costs impacting mating and reproduction. Thus, this data is not as accurate as we would like, but still provides a complementary lens to view the available data.

The above description is left as a record of what we tried, as it was a neat method for estimating allele frequencies, and as a reminder, as the caveats in the preceding paragraph could not be ignored.

Instead, the bulk sequencing data was integrated as provided: flies phenotypically negative for the gRNA constructs (i.e., $tdTom^{-}$) were sequenced and alleles categorized as wild-type or NHEJ, not specifying in-frame or out-of-frame. Three generations were sequenced for split drive, generations 2, 4, and 8, while four generations were sequenced for HACK, generations 2, 4, 8, and 20. Genotype combinations used for calculating simulated allele frequencies, for matching against the sequencing data in the likelihood, are provided in Table S6 for split drive, using Locus 2, and Table S7 for HACK.

Model Fitting:

A deterministic and stochastic simulation framework was developed by reducing the full MGDrivE into a discrete-generation model to reflect cage trial designs. The stochastic version implements distributions where the expectation matches the rates in the deterministic simulation. Mating follows a multinomial distribution over male genotypes, accounting for the male fitness costs described above. Egg-laying follows a multinomial distribution over offspring genotype, determined by maternal and paternal genotypes and the specific inheritance pattern being simulated. Number of offspring is Poisson-distributed, dependent on genotype-specific female fecundity, and sex distribution of offspring follows a binomial distribution, assuming equal probability. Adults in each generation are sampled equally for each sex, with an expected population size equal to the average size of each experimental generation, following a multivariate hypergeometric distribution.

The simulation framework implements the inheritance biases and fitness costs described above, and generates expected genotype frequencies in each generation, denoted p_k^x for genotype x in generation k and a_k^x for allele x in generation k. Genotype and allele frequencies are normalized post-simulation such that each generation sums to 1.

The likelihood of the population phenotype data was calculated by assuming a multinomial distribution of individuals having each sex and marker phenotype, and by using the model predictions to generate expected proportions for each set of parameter values. I.e., by calculating the log likelihood given in equation 5.

(5) $logL(\theta) \propto \sum_{i=1}^{3} \quad \sum_{k=1}^{n_i} \quad \sum_{P \in \{P_F, P_M\}} \quad N_{i,k}^P \cdot log(p_k^P(\theta))$

The log-likelihood is a summation over the three replicates for each system, *i*, over each generation, *k*, and over each phenotype for females, P_F, and males, P_M, denoted collectively by P. $N_{i,k}^P$ denotes the number of individuals at generation *k* in experiment *i* having phenotype P. Possible phenotypes with corresponding genotype-phenotype mappings are given in Table S4 (split-drive) and Table S5 (HACK). The ith experiment is run for n_i generations, and expected phenotype frequencies, $p_k^P(\theta)$, at generation *k* are dependent on the model parameters,

$\theta_{split-drive} = \{c_F^H, p_F^{HDR}, p_F^{NHEJ}, c_M^H, p_M^{HDR}, p_M^{NHEJ}, c^S, s_{c/g}, s_{NHEJ}, s_{NHEJ^2}, s_{cas9}\}$ or $\theta_{HACK} = \{c_F^H, p_F^{HDR}, p_F^{NHEJ}, c_M^H, p_M^{HDR}, p_M^{NHEJ}, c^S, s_{c/g}, s_{NHEJ}, s_{NHEJ^2}\}$ (see Tables S9-S112 for parameter definitions and estimates for each construct).

For the bulk-sequencing data, we used the phenotype mappings (Table S4 for split drive and Table S5 for HACK) to filter for gRNA$^-$ (tdTom$^-$) flies. From that filtered set of counts, we calculated allele frequencies for wild-type or NHEJ (combined in-frame and out-of-frame) alleles as described in Table S6, Locus 2, for split drive and Table S7 for HACK. This allowed us to avoid making assumptions about the real data, by filtering the synthetic data for agreement with the bulk-sequencing results. The likelihood for the sequencing data is provided in equation 6.

(6) $logL(\theta) \propto \sum_{i=3} \quad \sum_{k\in\{generations\}} \quad 20 \cdot log(a_k\ (\theta))$

The log-likelihood is similar to above, but with notable differences. The sequencing was done on flies from cage 3, thus $i = 3$, and only from three generations, $k \in \{2,4,8\}$, for split-drive or four generations, $k \in \{2,4,8,20\}$, for HACK. Additionally, male and females flies were mixed, and only 20 flies were sequenced from each of those generations, $N_{i,k}^P = 20$. Therefore, $a_k\ (\theta)$ was calculated by combining male and female counts, filtered by phenotype as described above, and calculated for wild-type or NHEJ allele frequency only. The full likelihood is equation 5 plus equation 6.

Experiments were seeded with a mixture of wild-type and heterozygous gene drive flies.
Each cage began with 120 flies, equally split between sexes, with 75% (90 count) wild-type and 25% (30 count) heterozygous for the Cas9 and gRNA elements. For the split drive, this corresponds to 90 “WWWW” and 30 “CWGW” flies, using genotypes from Table S4. For HACK, this is 90 “WW” and 30 “WH” flies, using genotypes from Table S5.

Models were fitted using a likelihood-based stochastic, gradient-free optimization method, an evolutionary algorithm from the R package DEoptim. A population of 250 was run for 500 generations using the default updating algorithm. For stochastic simulations, a trajectory-averaging method was used, based on 50 stochastic realizations of the model. Population M.A.P.

values were provided by the software. Covariance was calculated using the built-in cor() function in the stats library, using the pearson method, and quantiles were calculated using the quantile() function from the stats library, using method 8 for distribution-free, empirical quantiles. Correlations were visualized using corrplot() from the corrplot package. The estimated parameters, along with some statistics about each parameter, are included in Tables S10-S13. Deterministic fits are visualized with the corresponding cage trial and sequencing data in Figures S5-S8. Correlations are visualized in Figure S9 (split drive) and Figure S10 (HACK).

Stochastic realizations for Figure 4 (main manuscript) were generated using the deterministic fits of active Cas9/gRNA fitness costs. All simulations were performed, analyzed, and plotted in R with code/data available upon request.

Sensitivity Analysis:

As a test for future projects and as part of the responsible application of synthetic models, a partial-rank correlation coefficient (PRCC) analysis was performed, following the examples of Marino *et al*. This is a global sensitivity analysis with the additional metric of providing an importance measure (*p*-value), using equation 7 from their paper. Parameter sampling was performed using the Latin Hypercube technique (LHS) with the same parameter ranges as the fitting scripts, using the randomLHS() function from lhs. 500 samples were used and tornado plots with the results are provided in Figures S11 and S12.

Interpretation by the Modeler:

There are many things to consider in this supplement: new fitness cost comparisons, the second instance of mixed-data likelihoods, a new optimization scheme, stochastic objective functions, and a global sensitivity analysis.

The *spo11* autosomal split-drive data is not new, but was previously explored thoroughly in Terradas *et al.* ([12] in the main manuscript). However, the manuscript fits an active Cas9 cleavage/homing cost only, using an MCMC methodology. MCMC is slow, due to the linear nature

of the chains, but there are other MC methods that are trivially parallelizable for significant performance improvements. We chose an evolutionary algorithm, though any particle filter methods perform similarly, and again fit active cleavage/homing costs to recapitulate the previous results. Using the same parameter bounds as before, our new method matched the results shown in Terradas *et al.* (data not shown). We then updated the parameter bounds to more accurately reflect the single-pair crossings (an overlooked detail previously) and updated potential fitness costs, adding the possibility for compound LOF homozygotes to be fertile (s_{NHEJ^2}) and a Cas9 expression cost for split drive (s_{cas9}). Drive parameters are in better agreement with estimates obtained from single-pair crossings, and provide excellent phenotypic agreement between the data and simulations.

Since our new optimization method is consistent with our previous approach, we chose to explore new avenues for model fitting. Until now, we have only ever fit deterministic models. Particle swarms and genetic algorithms have a precedent for using stochastic objective functions, performing well on individual based models. Therefore, we tested stochastic model fits, implementing a simple trajectory-averaging method over parameter estimates. This is one of two naive averaging methods, the other using a larger population to obtain averages without directly averaging each parameter estimate. The proper method would use a variance-based approach to estimate and track the Pareto front, however, that approach is complicated and time consuming, while our models are simple enough that the computational efficiency afforded by that technique is not worth the effort. In theory, the same trajectory-averaging technique we use here should be applicable to MCMC approaches, however, we have not heard of this in practice.

It is an open question whether fitness costs associated with Cas9/gRNA are due to active cleavage and damage incurred from un-repaired chromosomes, or generic expression of Cas9/gRNA and minor off-target effects. Since we have previous work to build from, and a significantly faster optimization methodology, it seemed logical to test for differences between the two fitness cost implementations. For the split construct, HDR rates increase for co-occurrence costs compared to active-homing costs. However, both are within estimation of single-pair data, so we cannot

say that one is more appropriate than the other. Initially, when testing HACK with unconstrained parameters, active costs were clearly more appropriate than co-occurrence costs. But, after constraining estimates to experimentally appropriate parameter ranges, that difference disappeared. Even worse, the parameter estimates for HACK are exactly the same for both fitness cost implementations.

The identical parameter estimates for HACK bring up an interesting issue - an extremely flat likelihood landscape. The best-estimate parameters, with unbounded ranges, are less than 0.07% better than the estimates in Table S12. This is likely due to the paucity of phenotypic information: 10 genotypes collapsing into 2 phenotypes. To alleviate this, we incorporated bulk-sequencing information, our second time since Kaduskar *et al.* (nature.com/articles/s41467-021-27654-1). However, the sequencing data is trivial compared to the phenotypic data, using 20 flies per generation, 80 total flies, compared to 19386 flies in the HACK phenotypical data. It had a marginal effect on the fitting results.

Though we have similar likelihood landscape issues with split drive as we do for HACK, the projected allele frequency behavior is significantly different (Figures S5 and S6). Again, there is not enough data to distinguish between active homing vs co-occurrence as a proper cost model, but neither condition tracks the allele frequencies, implying a significant deviation from the expected inheritance pattern of a split-drive. This discrepancy highlights the issue of just using phenotype data, which the model matches very well, without a more direct measure of genotype transmission. This is a common failing of hidden Markov models, and one solution is using disjoint data as another part of the objective function, providing contrasting measures of the model. Our next step is to remove the assumption of Mendelian Inheritance between the two loci and test for linkage disequilibrium in the split-drive.

We regularly measure correlation between parameters during model fitting but rarely publish the results. In highly-parameterized models estimated from small amounts of data, e.g. our current situation, it is common to find compensatory changes in parameter estimates. For

example, in the top-row of Figure S9, we see the fitness costs of LOF carriers (“B” and “BB” in the panel) are negatively correlated with the fitness costs of gene drive individuals. This implies that some parameters are acting in concert, and we may be measuring the same effect twice, or that we have too many parameters, and there is some non-determination occurring. We know that the Cas9 expression cost acts together with the cleave or co-occurrence costs, so finding those correlated is not surprising. The two distinct blocks in split-drive are intriguing, and we are still trying to understand why parameters correlated that way. These correlations are also sensitive to the optimum obtained, as the population stays near the optimum for several generations, and a different optimum can have a different local parameter landscape.

Finally, we implemented a global sensitivity analysis, the Partial Rank Correlation. While not necessary for this analysis, the results are interesting. All models indicate that fitness costs are negatively associated with the likelihood, with Cas9/gRNA and (if relevant) Cas9 expression as the leading correlates. That is interesting because these are such small trials, being discrete generation and without density-dependent issues. If there were ever conditions where a gene drive would experience little or no fitness costs, this would be it. While this is interesting, it is also necessary to acknowledge the shortcomings of this analysis. PRCC is robust to non-linearities and weakly-correlated parameters. Population models like this, especially using a likelihood as the objective function, are not entirely linear. Additionally, several parameters are highly correlated (Figures S11 and S12). Cleavage, HDR, and resistance generation rates are mathematically written to depend on one another (see Inheritance Design, either sub-section). It may be possible, checking each parameter individually, to find subsets of parameter space where they are independent and linearly related to the objective function for appropriate application of the PRCC.

Future work could go in several directions. The use of a stochastic objective function was explored, but its value compared to the deterministic approach is still uncertain. Multiple data sources were successfully incorporated, but the value of the sequencing data was negligible compared to the phenotype data, so the value added is potentially low. Alternative, more high-

value data sources to create greater contrast in the likelihood landscape would be valuable. Our exploration of different fitness-cost implementations was ad hoc and could be improved through a proper power analysis. However, we feel that the dearth of data, which also caused parameter identification issues, negates the utility of a full power analysis in this situation. Finally, the sensitivity analysis is not entirely appropriate for the simulations, due to significant non-linearities and strong correlations, and it would be prudent to explore variance-based methods such as Sobol's method or eFAST.

Table S2 - Autosomal split-drive allele descriptions and fluorescent labels

Locus 1, located on chromosome III, carries the Cas9 (denoted C, labeled with EGFP) allele. When there is no Cas9 present, that locus can take either the wild-type (W) designation, or in special circumstances, the shadow drive (S) designation, neither of which have a fluorescent label. The S "allele" denotes the case where females are wild-type at the Cas9 locus, but their mothers have the C allele, which they inherit through protein deposition from their mother. As the Cas9 locus is static, it is always inherited in a Mendelian fashion, so resistance alleles are not relevant. Locus 2, located on chromosome II, contains the gRNA (G, labeled with tdTom) locus. When not present, the wild-type (W) allele and NHEJ alleles all appear without fluorescent labels. NHEJ alleles fall into one of two categories: functional (denoted R for "resistant") alleles, and non-functional (denoted B for "broken") alleles.

Locus 1 (Chromosome III)			Locus 2 (Chromosome II)		
**Allele**	**Description**	**Fluorescent Label**	**Allele**	**Description**	**Fluorescent Label**
W	Wild-type	Null	W	Wild-type	Null
C	Cas9	EGFP	G	gRNAs	tdTom
S	Shadow drive	Null	R	Functional NHEJ	Null
			B	Non-functional NHEJ	Null

Table S3 - HACK allele descriptions and fluorescent labels

The HACK construct is created when the Cas9 cassette combines with the gRNA cassette at the gRNA locus on Chromosome II. Cas9 is labeled with EGFP and gRNAs are labeled with tdTom. When present, the wild-type (W) allele and NHEJ alleles all appear without fluorescent labels. NHEJ alleles fall into one of two categories: functional (denoted R for "resistant") alleles, and non-functional (denoted B for "broken") alleles.

HACK (Chromosome 2)		
**Allele**	**Description**	**Fluorescent Label**
W	Wild-type	Null
H	Cas9/gRNAs	EGFP/tdTom
R	Functional NHEJ	Null
B	Non-functional NHEJ	Null

Table S4 - Autosomal split-drive phenotype-to-genotype mappings

As there are four fluorescent labels (EGFP, tdTom, and none, see Table S1), there are four possible phenotypes. These phenotypes provide some indication of the genotype of an individual, as each genotype generates a specific phenotype. From that, we can classify all genotypes as one of four phenotypes observed in the experiments. For each genotype, the first two letters denote locus 1 (W, C, S), and the latter two letters denote locus 2 (W, G, R, B). As the loci are on different chromosomes, they segregate independently.

Observed Phenotype	Possible Genotype
White	WWWW, WWRW, WWBW, WWRR, WWBR, WWBB, SWWW, SWRW, SWBW, SWRR, SWBR, SWBB
Green (EGFP)	CWWW, CWRW, CWBW, CWRR, CWBR, CWBB, CCWW, CCRW, CCBW, CCRR, CCBR, CCBB, CSWW, CSRW, CSBW, CSRR, CSBR, CSBB
Red (tdTom)	WWGW, WWGG, WWGR, WWBG, SWGW, SWGG, SWGR, SWBG
Both (EGFP/tdTom)	CWGW, CWGG, CWGR, CWBG, CCGW, CCGG, CCGR, CCBG, CSGW, CSGG, CSGR, CSBG

Table S5 - HACK phenotype-to-genotype mappings

As there are three fluorescent labels (EGFP, tdTom, and none, see Table S2), but as the EGFP and tdTom are linked, there's only two possible phenotypes. These phenotypes provide some indication of the genotype of an individual, as each genotype generates a specific phenotype. From that, we can classify the genotypes into one of the two phenotypes observed.

Observed Phenotype	Possible Genotype
White	WW, WR, WB, RR, RB, BB
Both (EGFP/tdTom)	WH, HH, HR, HB

Table S6 - Autosomal split-drive allele-to-genotype mappings

From the bulk sequencing, we were able to estimate allele frequencies. As we modeled diploid organisms, we have to aggregate the frequencies of several genotypes to match the allele frequencies. The genotypes combined to calculate each allele frequency are provided in the right column. As each locus is independent, we aggregate separately for the first and second loci. Genotypes that provide two copies of an allele, such as homozygous wild-type individuals (WWWW), are listed twice because they must be counted twice.

Locus 1 (Chromosome III)	
**Allele**	**Genotype Contributions**
W	WWWW, WWGW, WWRW, WWBW, WWGG, WWGR, WWBG, WWRR, WWBR, WWBB, WWWW, WWGW, WWRW, WWBW, WWGG, WWGR, WWBG, WWRR, WWBR, WWBB, CWWW, CWGW, CWRW, CWBW, CWGG, CWGR, CWBG, CWRR, CWBR, CWBB, SWWW, SWGW, SWRW, SWBW, SWGG, SWGR, SWBG, SWRR, SWBR, SWBB
C	CWWW, CWGW, CWRW, CWBW, CWGG, CWGR, CWBG, CWRR, CWBR, CWBB, CCWW, CCGW, CCRW, CCBW, CCGG, CCGR, CCBG, CCRR, CCBR, CCBB, CCWW, CCGW, CCRW, CCBW, CCGG, CCGR, CCBG, CCRR, CCBR, CCBB, CSWW, CSGW, CSRW, CSBW, CSGG, CSGR, CSBG, CSRR, CSBR, CSBB
S	SWWW, SWGW, SWRW, SWBW, SWGG, SWGR, SWBG, SWRR, SWBR, SWBB, CSWW, CSGW, CSRW, CSBW, CSGG, CSGR, CSBG, CSRR, CSBR, CSBB
**Locus 2 (Chromosome II)**	
**Allele**	**Genotype Contributions**
W	WWWW, CWWW, SWWW, CCWW, CSWW, WWWW, CWWW, SWWW, CCWW, CSWW, WWGW, CWGW, SWGW, CCGW, CSGW, WWRW, CWRW, SWRW, CCRW, CSRW, WWBW, CWBW, SWBW, CCBW, CSBW
G	WWGW, CWGW, SWGW, CCGW, CSGW, WWGG, CWGG, SWGG, CCGG, CSGG, WWGG, CWGG, SWGG, CCGG, CSGG, WWGR, CWGR, SWGR, CCGR, CSGR, WWBG, CWBG, SWBG, CCBG, CSBG
R	WWRW, CWRW, SWRW, CCRW, CSRW, WWGR, CWGR, SWGR, CCGR, CSGR, WWRR, CWRR, SWRR, CCRR, CSRR, WWRR, CWRR, SWRR, CCRR, CSRR, WWBR, CWBR, SWBR, CCBR, CSBR
B	WWBW, CWBW, SWBW, CCBW, CSBW, WWBG, CWBG, SWBG, CCBG, CSBG, WWBR, CWBR, SWBR, CCBR, CSBR, WWBB, CWBB, SWBB, CCBB, CSBB, WWBB, CWBB, SWBB, CCBB, CSBB

Table S7 - HACK allele-to-genotype mappings

From the bulk sequencing, we were able to estimate allele frequencies. As we modeled diploid organisms, we have to aggregate the frequencies of several genotypes to match the allele frequencies. The genotypes combined to calculate each allele frequency are provided in the right column. Genotypes that provide two copies of an allele, such as homozygous wild-type individuals (WW), are listed twice because they must be counted twice.

Allele	Genotype Contributions
W	WW, WW, WH, WR, WB
H	WH, HH, HH, HR, HB
R	WR, HR, RR, RR, RB
B	WB, HB, RB, BB, BB

Table S8 - Split drive fitness cost implementation

This table demonstrates how fitness costs were implemented under the two models - Cas9/gRNA cost under active cleavage/homing (top) or co-occurrence (bottom). Fitness costs were implemented the same, simply applied to the appropriate genotypes.

**Active Fitness Cost**	
**Genotype**	**Fitness Cost**
CWWW, CWRW, CWGG, CWGR, CWRR, CCWW, CCRW, CCGG, CCGR, CCRR, CSWW, CSRW, CSGG, CSGR, CSRR	s_{cas9}
SWGW	$s_{c/g}$
WWBW, WWBG, WWBR, SWBW, SWBG, SWBR	s_{NHEJ}
WWBB, SWBB	$s_{NHEJ}\ ^2$
CWGW, CCGW, CSGW	$s_{cas9} + s_{c/g}$
CWBW, CWBG, CWBR, CCBW, CCBG, CCBR, CSBW, CSBG, CSBR	$s_{cas9} + s_{NHEJ}$
CWBB, CCBB, CSBB	$s_{cas9} + s_{NHEJ^2}$
**Existence Fitness Cost**	
**Genotype**	**Fitness Cost**
CWWW, CWRW, CWRR, CCWW, CCRW, CCRR, CSWW, CSRW, CSRR	s_{cas9}
SWGW, SWGG, SWGR	$s_{c/g}$
WWBW, WWBG, WWBR, SWBW, SWBR	s_{NHEJ}
WWBB, SWBB	$s_{NHEJ}\ ^2$
CWGW, CWGG, CWGR, CCGW, CCGG, CCGR, CSGW, CSGG, CSGR	$s_{cas9} + s_{c/g}$
CWBW, CWBR, CCBW, CCBR, CSBW, CSBR	$s_{cas9} + s_{NHEJ}$
CWBB, CCBB, CSBB	$s_{cas9} + s_{NHEJ^2}$
SWBG	$s_{c/g} + s_{NHEJ}$
CWBG, CCBG, CSBG	$s_{cas9} + s_{c/g} + s_{NHEJ}$

Table S9 - HACK fitness cost implementation

This table demonstrates how fitness costs were implemented under the two models - Cas9/gRNA cost under active cleavage/homing (top) or co-occurrence (bottom). Fitness costs were implemented the same, simply applied to the appropriate genotypes.

**Active Fitness Cost**	
**Genotype**	**Fitness Cost**
WH	$s_{c/g}$
WB, HB, RB	s_{NHEJ}
BB	$s_{NHEJ}{}^{2}$
**Existence Fitness Cost**	
**Genotype**	**Fitness Cost**
WH, HH, HR	$s_{c/g}$
WB, RB	s_{NHEJ}
HB	$s_{c/g} + s_{NHEJ}$
BB	$s_{NHEJ}{}^{2}$

Table S10 - Autosomal split-drive parameter estimates, Active cost

Parameters were estimated by running the log-likelihood, calculated on the deterministic simulation, through an evolutionary Monte Carlo algorithm on fitness costs applied only when active cleavage/homing occurred. The maximum a posteriori estimate (M.A.P.) is the point that appears most often and is the best point estimate for our parameters. Additionally, the 2.5%, 50%, and 97.5% quantiles are provided.

Parameters		Evolutionary Algorithm MC Parameter Estimates			
**Label**	**Description**	**M.A.P.**	**2.5% Quantile**	**Median**	**97.5% Quantile**
c_F^H	Female cleavage Rate	0.95	0.89	0.95	0.95
p_F^{HDR}	Female HDR rate	0.75	0.66	0.75	0.75
p_F^{NHEJ}	Female functional NHEJ rate	0.00	0.00	0.00	0.33
c_M^H	Male cleavage rate	0.95	0.86	0.95	0.95
p_M^{HDR}	Male HDR rate	0.40	0.32	0.40	0.40
p_M^{NHEJ}	Male functional NHEJ rate	0.00	0.00	0.00	0.35
c^S	Shadow drive cleavage rate	0.15	0.04	0.15	0.15
$s_{c/g}$	Fitness cost per Cas9/gRNA allele	0.00	0.00	0.00	0.17
s_{NHEJ}	Fitness cost of one NHEJ allele	0.40	0.10	0.40	0.40
s_{NHEJ^2}	Fitness cost of two NHEJ alleles	1.00	0.79	0.99	1.00
s_{cas9}	Fitness cost of Cas9 expression	0.01	0.00	0.01	0.15

Table S11 - Autosomal split-drive parameter estimates, Existence cost

In contrast to Table S8, parameters here reflect a fitness cost applied whenever Cas9/gRNA co-occurred, even when homing was not possible. Again, the log-likelihood calculated on the deterministic simulation was used in an evolutionary Monte Carlo algorithm to estimate parameters. The maximum a posteriori estimate (M.A.P.) is the point that appears most often and is the best point estimate for our parameters. Additionally, the 2.5%, 50%, and 97.5% quantiles are provided.

Parameters		Evolutionary Algorithm MC Parameter Estimates			
**Label**	**Description**	**M.A.P.**	**2.5% Quantile**	**Median**	**97.5% Quantile**
c_F^H	Female cleavage Rate	0.95	0.89	0.95	0.95
p_F^{HDR}	Female HDR rate	0.75	0.66	0.75	0.75
p_F^{NHEJ}	Female functional NHEJ rate	0.00	0.00	0.00	0.34
c_M^H	Male cleavage rate	0.95	0.86	0.95	0.95
p_M^{HDR}	Male HDR rate	0.40	0.31	0.40	0.40
p_M^{NHEJ}	Male functional NHEJ rate	0.00	0.00	0.00	0.35
c^S	Shadow drive cleavage rate	0.15	0.04	0.15	0.15
$s_{c/g}$	Fitness cost per Cas9/gRNA allele	0.00	0.00	0.00	0.17
s_{NHEJ}	Fitness cost of one NHEJ allele	0.40	0.11	0.40	0.40
s_{NHEJ^2}	Fitness cost of two NHEJ alleles	1.00	0.79	0.99	1.00
s_{cas9}	Fitness cost of Cas9 expression	0.01	0.00	0.01	0.16

Table S12 - HACK parameter estimates, Active cost

Parameters were estimated by running the log-likelihood, calculated on the deterministic simulation, through an evolutionary Monte Carlo algorithm on fitness costs applied only when active cleavage/homing occurred. The maximum a posteriori estimate (M.A.P.) is the point that appears most often and is the best point estimate for our parameters. Additionally, the 2.5%, 50%, and 97.5% quantiles are provided.

Parameters		Evolutionary Algorithm MC Parameter Estimates			
Label	Description	M.A.P.	2.5% Quantile	Median	97.5% Quantile
c_F^H	Female cleavage rate	0.95	0.87	0.95	0.95
p_F^{HDR}	Female HDR rate	0.49	0.41	0.48	0.63
p_F^{NHEJ}	Female functional NHEJ rate	0.50	0.22	0.50	0.50
c_M^H	Male cleavage rate	0.95	0.86	0.95	0.95
p_M^{HDR}	Male HDR rate	0.20	0.20	0.26	0.43
p_M^{NHEJ}	Male functional NHEJ rate	0.50	0.24	0.50	0.50
c^D	Female deposition cleavage rate	0.00	0.00	0.00	0.04
$s_{c/g}$	Fitness cost per Cas9/gRNA allele	0.00	0.00	0.04	0.27
s_{NHEJ}	Fitness cost of one NHEJ allele	0.00	0.00	0.00	0.29
s_{NHEJ^2}	Fitness cost of two NHEJ alleles	0.90	0.90	0.90	0.98

Table S13 - HACK parameter estimates, Existence cost

In contrast to Table S10, parameters were estimated using the fitness cost applied to Cas9/gRNA co-occurrence. Other than the implementation of fitness costs, the same log-likelihood, deterministic simulation, and evolutionary Monte Carlo algorithm were used to estimate parameter values. The maximum a posteriori estimate (M.A.P.) is the point that appears most often and is the best point estimate for our parameters. Additionally, the 2.5%, 50%, and 97.5% quantiles are provided.

Parameters		Evolutionary Algorithm MC Parameter Estimates			
**Label**	**Description**	**M.A.P.**	**2.5% Quantile**	**Median**	**97.5% Quantile**
c_F^H	Female cleavage rate	0.95	0.87	0.94	0.95
p_F^{HDR}	Female HDR rate	0.54	0.42	0.54	0.64
p_F^{NHEJ}	Female functional NHEJ rate	0.49	0.08	0.42	0.50
c_M^H	Male cleavage rate	0.95	0.84	0.94	0.95
p_M^{HDR}	Male HDR rate	0.20	0.21	0.32	0.44
p_M^{NHEJ}	Male functional NHEJ rate	0.50	0.08	0.42	0.50
c^D	Female deposition cleavage rate	0.00	0.00	0.01	0.05
$s_{c/g}$	Fitness cost per Cas9/gRNA allele	0.02	0.01	0.08	0.23
s_{NHEJ}	Fitness cost of one NHEJ allele	0.00	0.00	0.01	0.31
s_{NHEJ^2}	Fitness cost of two NHEJ alleles	0.90	0.90	0.90	0.98

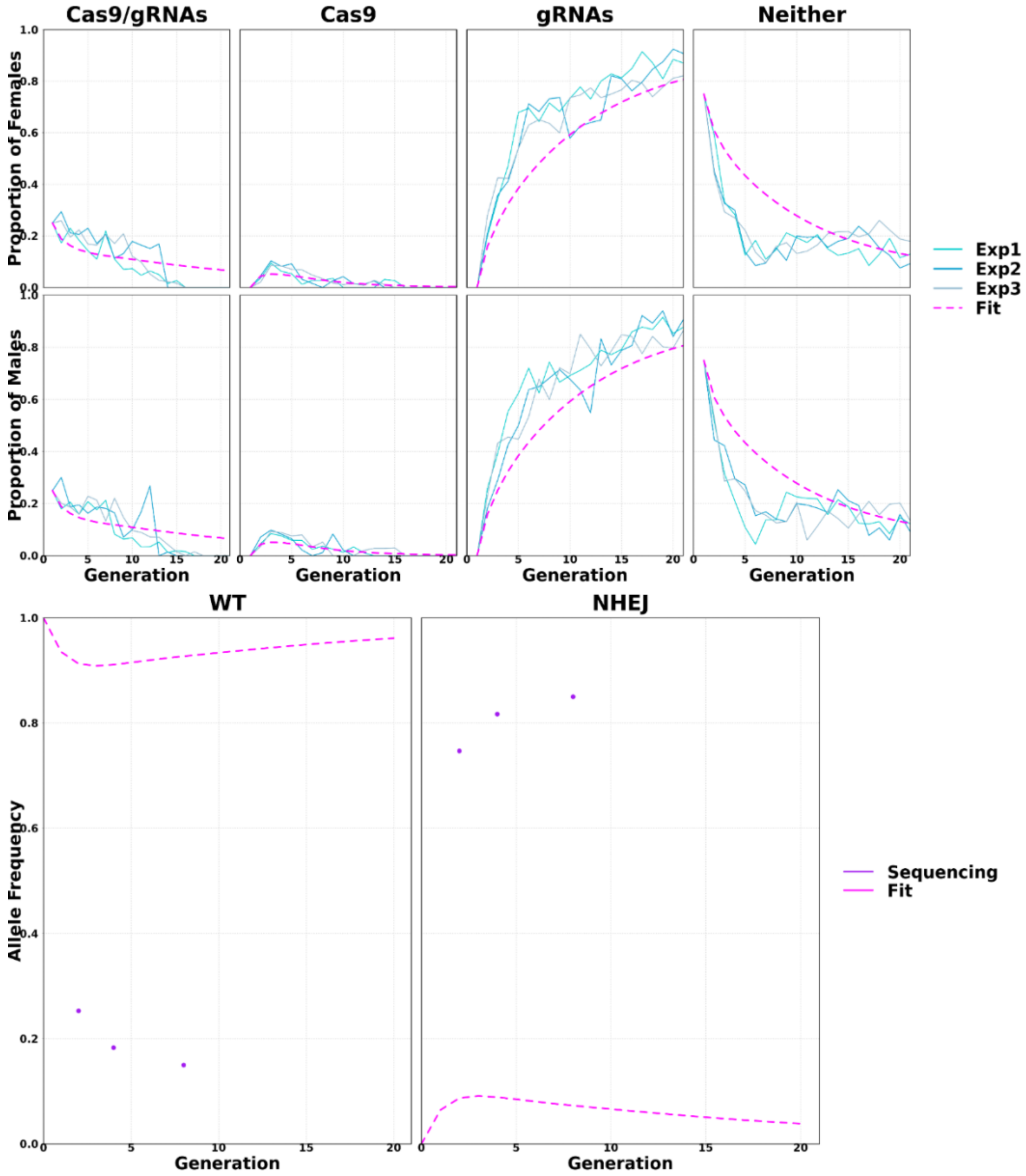

Figure S5 - Autosomal split-drive cage trials and Model fit, Active cost

Observed (solid blue lines on top and purple dots on bottom) and model-predicted (dashed, magenta lines) population dynamics for the autosomal *spo11* split drive. The top plot displays phenotype measurements and predictions, defined in Tables S1 and S3, and the bottom plot summarizes bulk-sequencing data with allele-frequencies calculated from the fits, defined in the lower-half (Locus 2) of Table S5. Sequencing data was only obtained at 3 generations (2, 4, and 8), while phenotypes were recorded at every generation. The deterministic model was used for these plots, though the stochastic implementation was also tested (and not plotted due to space). This fit implemented the active-homing costs defined in the top-half of Table S7 (Active Fitness Cost) and the results are provided in Table S9.

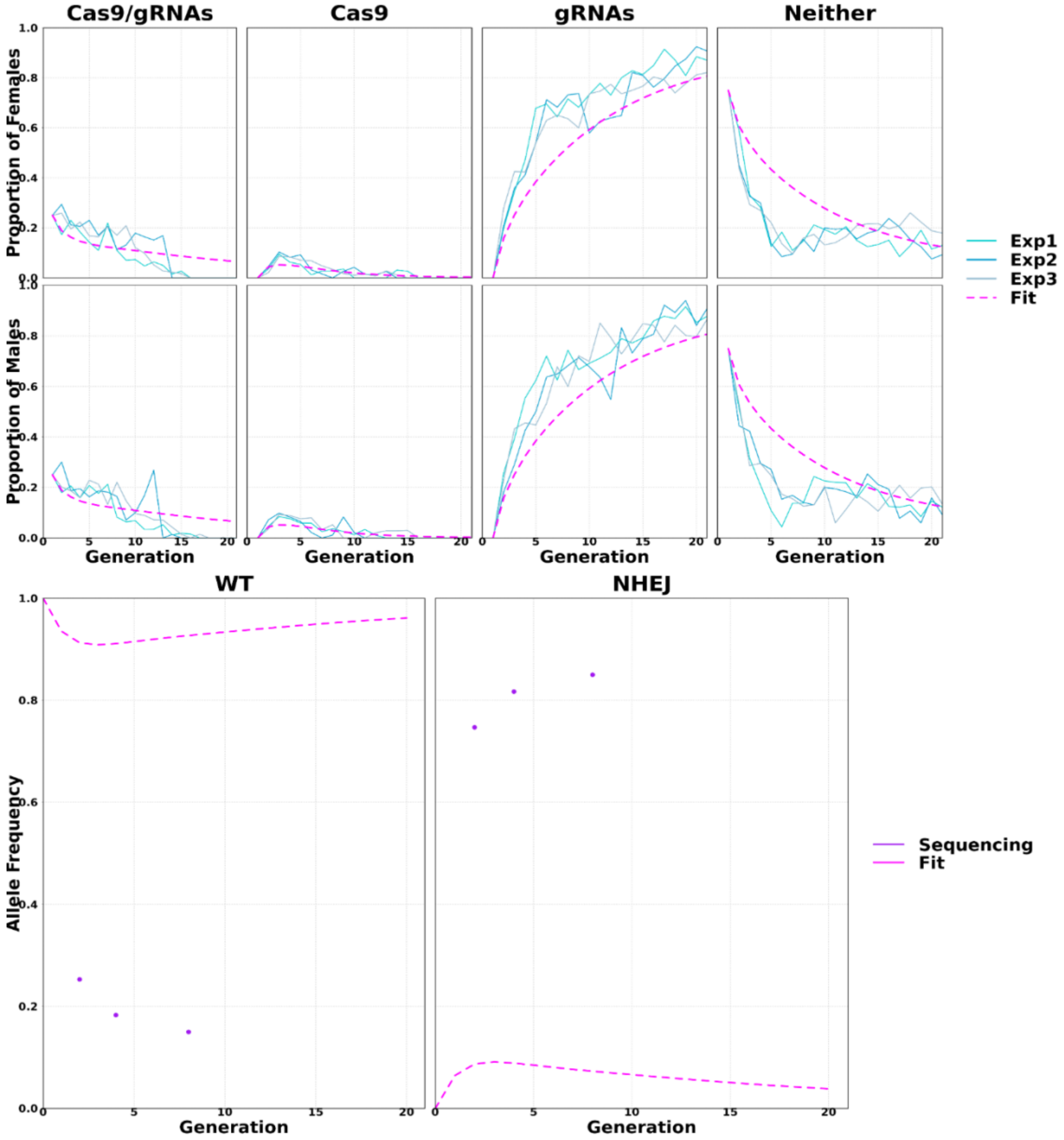

Figure S6 - Autosomal split-drive cage trials and Model fit, Existence Cost

Observed (solid blue lines on top and purple dots on bottom) and model-predicted (dashed, magenta lines) population dynamics for the autosomal *spo11* split-drive. The top plot displays phenotype measurements and predictions, defined in Tables S1 and S3, and the bottom plot summarizes bulk-sequencing data with allele-frequencies calculated from the fits, defined in the lower-half (Locus 2) of Table S5. Sequencing data was only obtained at 3 generations (2, 4, and 8), while phenotypes were recorded at every generation. The deterministic model was used for these plots, though the stochastic implementation was also tested (and not plotted due to space). This fit used fitness costs applied to any combination of Cas9 with gRNA, as defined in the bottom-half of Table S7 (Existence Fitness Cost), and the results are provided in Table S10.

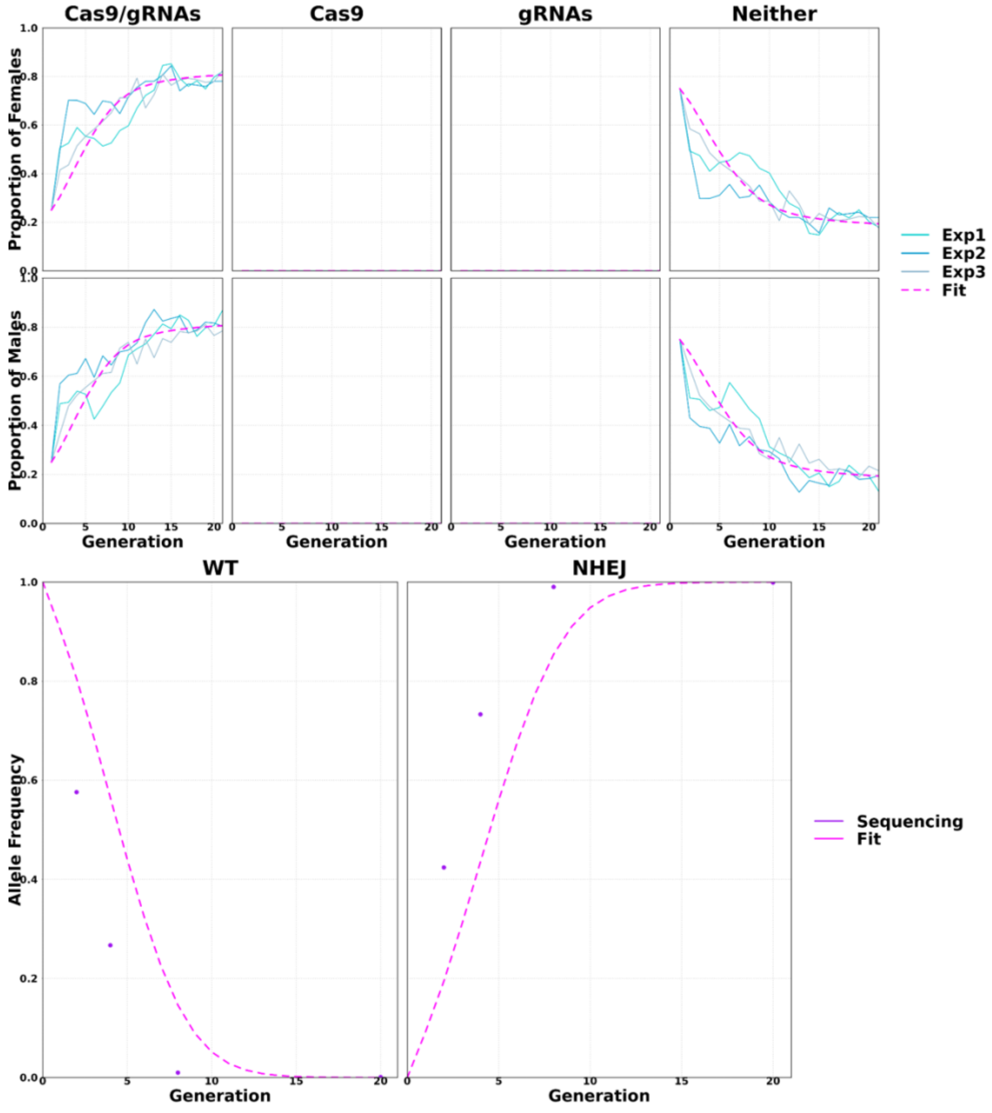

Figure S7 - HACK cage trials and Model fit, Active cost

Observed (solid blue lines on top and purple dots on bottom) and model-predicted (dashed, magenta lines) population dynamics for the *spo11* HACK construct. The top plot displays phenotypic data and fits, defined in Tables S2 and S4, while the bottom plot summarizes bulk-sequencing data with allele-frequencies calculated from the fits, defined in Table S6. Sequencing data was only obtained at four generations (2, 4, 8, and 20), while phenotypes were recorded at every generation. Phenotypic matching is excellent, while sequencing data matches in trend but not exactly in value. This could be due to the small number of flies sequenced (20 per generation) or the flies chosen for sequencing (fGD^- only). The deterministic model was used for these plots, though the stochastic implementation was also tested (and not plotted due to space). This fit implemented the active-homing costs defined in the top-half of Table S8 (Active Fitness Cost) and the results are provided in Table S11.

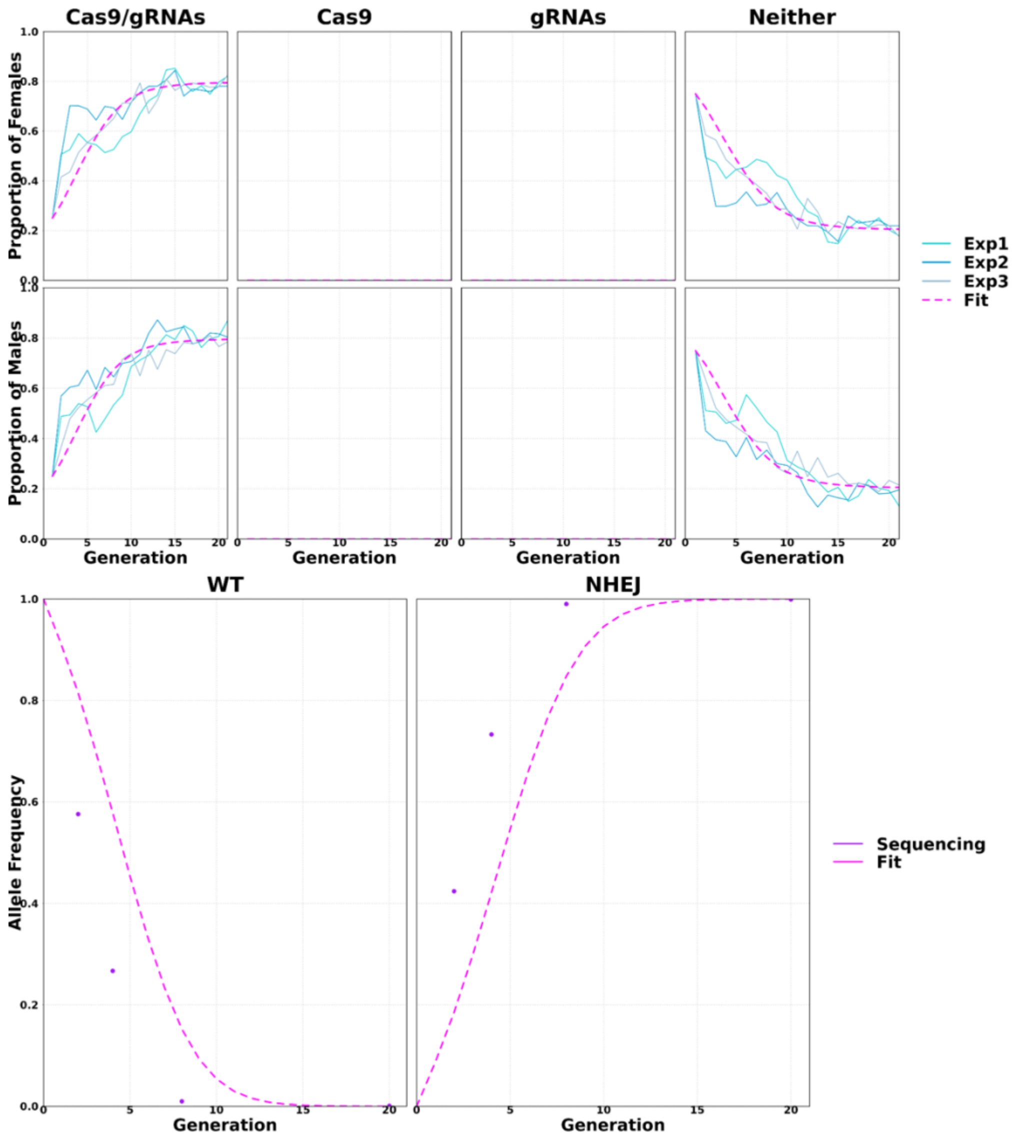

Figure S8 - HACK cage trials and Model fit, Existence Cost

Observed (solid blue lines on top and purple dots on bottom) and model-predicted (dashed, magenta lines) population dynamics for the *spo11* HACK construct. The top plot displays phenotypic data and fits, defined in Tables S2 and S4, while the bottom plot summarizes bulk-sequencing data with allele-frequencies calculated from the fits, defined in Table S6. Sequencing data was only obtained at four generations (2, 4, 8, and 20), while phenotypes were recorded at every generation. Phenotypic matching is excellent, while sequencing data matches in trend but not exactly in value. This could be due to the small number of flies sequenced (20 per generation) or the flies chosen for sequencing (fGD^{-} only). The deterministic model was used for these plots, though the stochastic implementation was also tested (and not plotted due to space). This fit used fitness costs applied to any combination of Cas9 with gRNA, as defined in the bottom-half of Table S8 (Existence Fitness Cost) and the results are provided in Table S12.

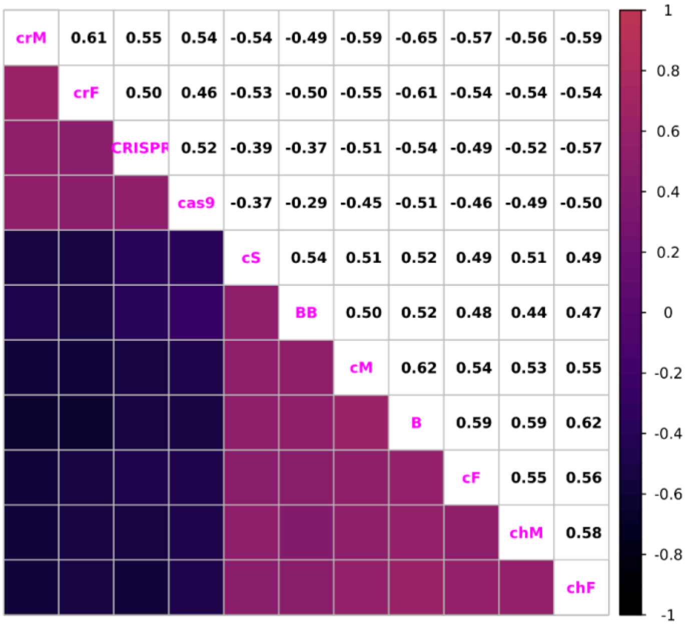
crM 0.61 0.55 0.54 -0.54 -0.49 -0.59 -0.65 -0.57 -0.56 -0.59
crF 0.50 0.46 -0.53 -0.50 -0.55 -0.61 -0.54 -0.54 -0.54
CRISPR 0.52 -0.39 -0.37 -0.51 -0.54 -0.49 -0.52 -0.57
cas9 -0.37 -0.29 -0.45 -0.51 -0.46 -0.49 -0.50
cS 0.54 0.51 0.52 0.49 0.51 0.49
BB 0.50 0.52 0.48 0.44 0.47
cM 0.62 0.54 0.53 0.55
B 0.59 0.59 0.62
cF 0.55 0.56
chM 0.58
chF
1
0.8
0.6
0.4
0.2
0
-0.2
-0.4
-0.6
-0.8
-1

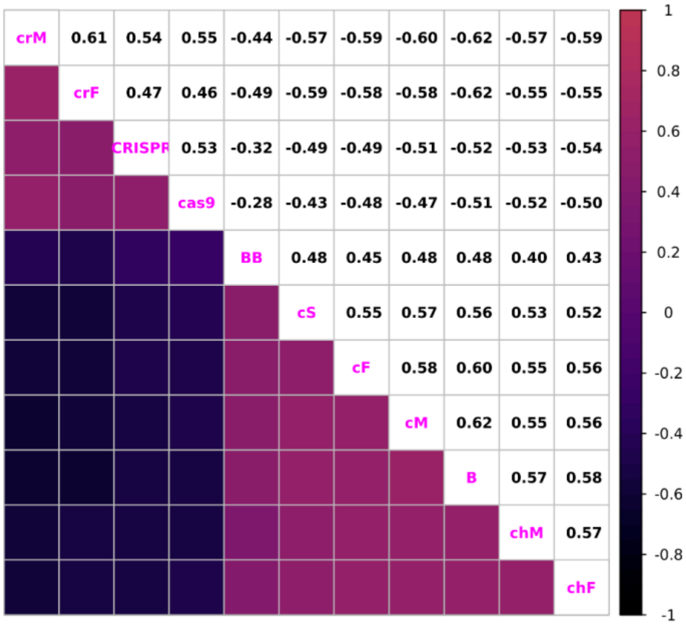
crM 0.61 0.54 0.55 -0.44 -0.57 -0.59 -0.60 -0.62 -0.57 -0.59
crF 0.47 0.46 -0.49 -0.59 -0.58 -0.58 -0.62 -0.55 -0.55
CRISPR 0.53 -0.32 -0.49 -0.49 -0.51 -0.52 -0.53 -0.54
cas9 -0.28 -0.43 -0.48 -0.47 -0.51 -0.52 -0.50
BB 0.48 0.45 0.48 0.48 0.40 0.43
cS 0.55 0.57 0.56 0.53 0.52
cF 0.58 0.60 0.55 0.56
cM 0.62 0.55 0.56
B 0.57 0.58
chM 0.57
chF
1
0.8
0.6
0.4
0.2
0
-0.2
-0.4
-0.6
-0.8
-1

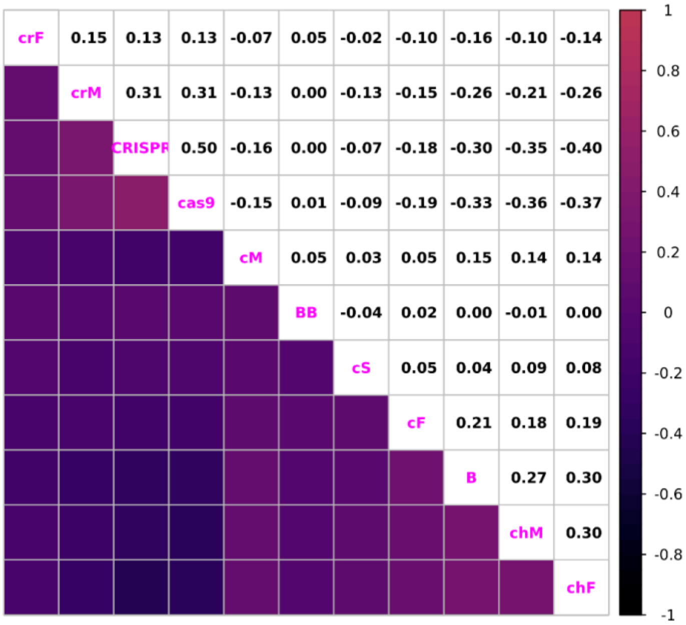
crF 0.15 0.13 0.13 -0.07 0.05 -0.02 -0.10 -0.16 -0.10 -0.14
crM 0.31 0.31 -0.13 0.00 -0.13 -0.15 -0.26 -0.21 -0.26
CRISPR 0.50 -0.16 0.00 -0.07 -0.18 -0.30 -0.35 -0.40
cas9 -0.15 0.01 -0.09 -0.19 -0.33 -0.36 -0.37
cM 0.05 0.03 0.05 0.15 0.14 0.14
BB -0.04 0.02 0.00 -0.01 0.00
cS 0.05 0.04 0.09 0.08
cF 0.21 0.18 0.19
B 0.27 0.30
chM 0.30
chF
1
0.8
0.6
0.4
0.2
0
-0.2
-0.4
-0.6
-0.8
-1

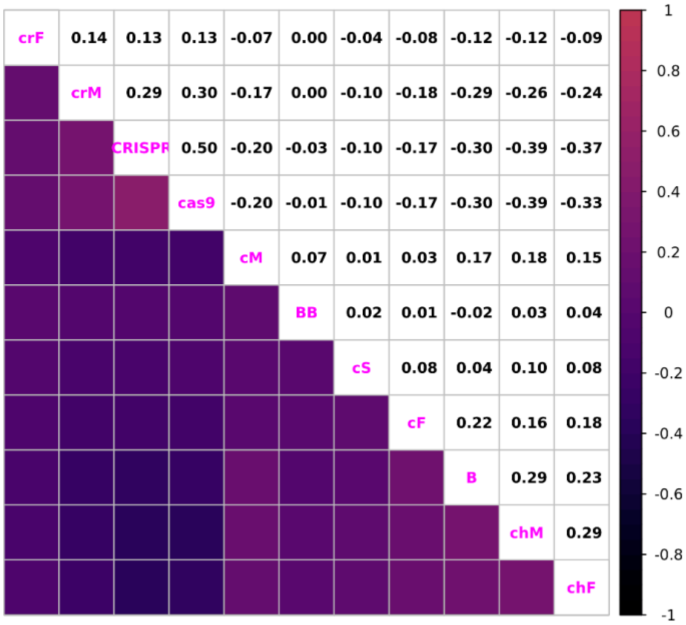
crF 0.14 0.13 0.13 -0.07 0.00 -0.04 -0.08 -0.12 -0.12 -0.09
crM 0.29 0.30 -0.17 0.00 -0.10 -0.18 -0.29 -0.26 -0.24
CRISPR 0.50 -0.20 -0.03 -0.10 -0.17 -0.30 -0.39 -0.37
cas9 -0.20 -0.01 -0.10 -0.17 -0.30 -0.39 -0.33
cM 0.07 0.01 0.03 0.17 0.18 0.15
BB 0.02 0.01 -0.02 0.03 0.04
cS 0.08 0.04 0.10 0.08
cF 0.22 0.16 0.18
B 0.29 0.23
chM 0.29
chF
1
0.8
0.6
0.4
0.2
0
-0.2
-0.4
-0.6
-0.8
-1

Figure S9 - Correlation analysis of autosomal split-drive Model fits

These plots measure the correlation between parameter estimates during model optimization, displayed as shades (lower-left triangle, scale on right) with their value (upper-right triangle). Correlation measures the direction (positive or negative number) and magnitude (absolute size of each number) of interactions between variable estimates. The top-row presents deterministic simulations and the bottom row presents stochastic simulations. The left column implements active-homing costs (Table S7, top) and the right column implements co-occurrence costs (Table S7, bottom). In the deterministic simulations, we see strong correlations between the resistance-generation rate (in males and females) and fitness costs from active homing and Cas9 expression, and then between cleavage, homology-directed repair, and NHEJ fitness costs. These correlations appear in active and co-occurrence models, and hold under stochastic simulation (though are weaker).

(c{M/F} - male/female cleavage, ch{M/F} - male/female HDR, cr{M/F} - male/female in-frame NHEJ, cS - rate of shadow drive, CRISPR - cost associated with Cas9/gRNA, B - mosaic cost of out-of-frame NHEJ allele, BB - reproductive cost of compound homozygous out-of-frame NHEJ alleles, cas9 - Cas9 expression cost, independent of cleavage.)

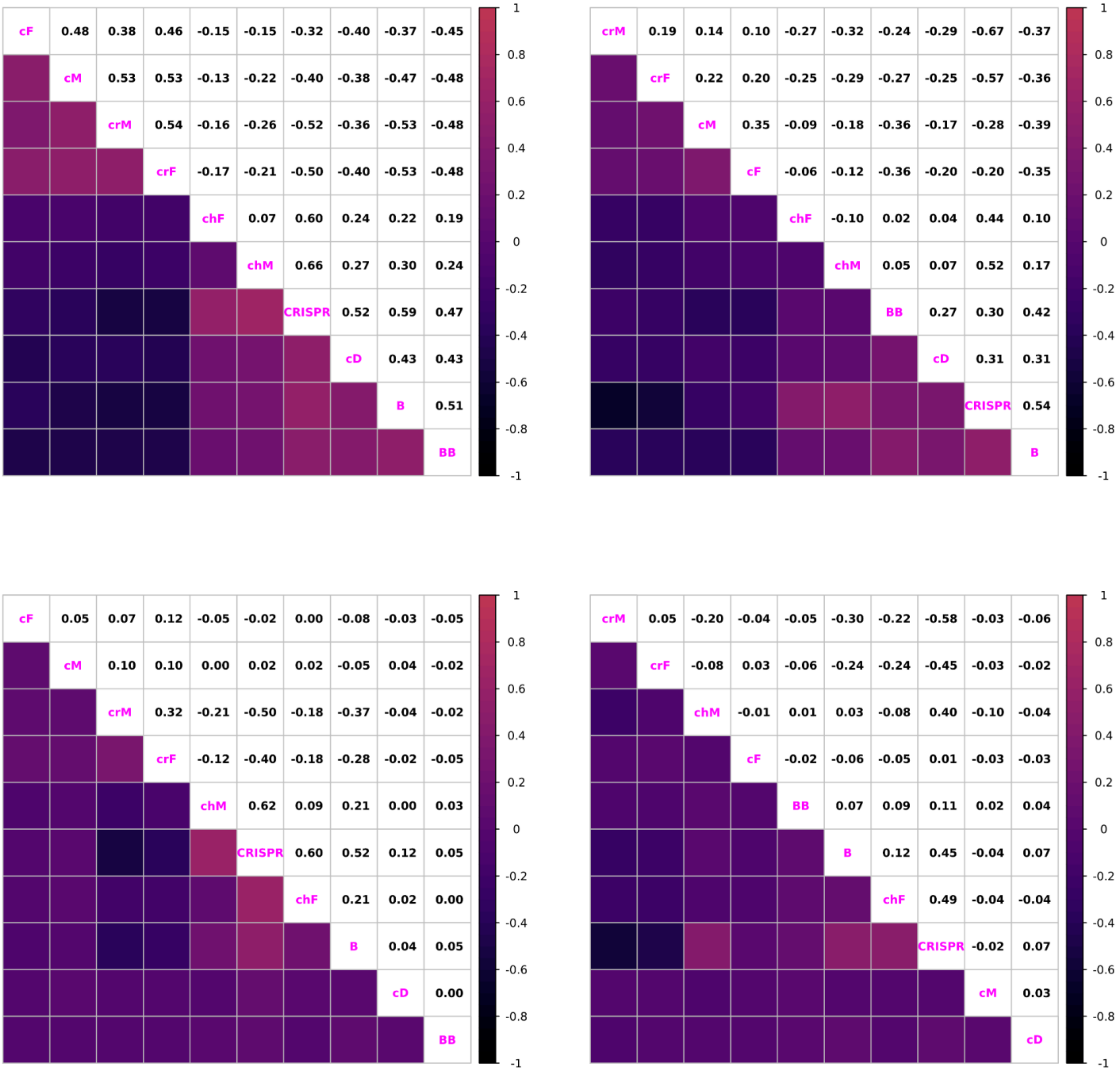
cF 0.48 0.38 0.46 -0.15 -0.15 -0.32 -0.40 -0.37 -0.45
cM 0.53 0.53 -0.13 -0.22 -0.40 -0.38 -0.47 -0.48
crM 0.54 -0.16 -0.26 -0.52 -0.36 -0.53 -0.48
crF -0.17 -0.21 -0.50 -0.40 -0.53 -0.48
chF 0.07 0.60 0.24 0.22 0.19
chM 0.66 0.27 0.30 0.24
CRISPR 0.52 0.59 0.47
cD 0.43 0.43
B 0.51
BB
1 0.8 0.6 0.4 0.2 0 -0.2 -0.4 -0.6 -0.8 -1
crM 0.19 0.14 0.10 -0.27 -0.32 -0.24 -0.29 -0.67 -0.37
crF 0.22 0.20 -0.25 -0.29 -0.27 -0.25 -0.57 -0.36
cM 0.35 -0.09 -0.18 -0.36 -0.17 -0.28 -0.39
cF -0.06 -0.12 -0.36 -0.20 -0.20 -0.35
chF -0.10 0.02 0.04 0.44 0.10
chM 0.05 0.07 0.52 0.17
BB 0.27 0.30 0.42
cD 0.31 0.31
CRISPR 0.54
B
1 0.8 0.6 0.4 0.2 0 -0.2 -0.4 -0.6 -0.8 -1
cF 0.05 0.07 0.12 -0.05 -0.02 0.00 -0.08 -0.03 -0.05
cM 0.10 0.10 0.00 0.02 0.02 -0.05 0.04 -0.02
crM 0.32 -0.21 -0.50 -0.18 -0.37 -0.04 -0.02
crF -0.12 -0.40 -0.18 -0.28 -0.02 -0.05
chM 0.62 0.09 0.21 0.00 0.03
CRISPR 0.60 0.52 0.12 0.05
chF 0.21 0.02 0.00
B 0.04 0.05
cD 0.00
BB
1 0.8 0.6 0.4 0.2 0 -0.2 -0.4 -0.6 -0.8 -1
crM 0.05 -0.20 -0.04 -0.05 -0.30 -0.22 -0.58 -0.03 -0.06
crF -0.08 0.03 -0.06 -0.24 -0.24 -0.45 -0.03 -0.02
chM -0.01 0.01 0.03 -0.08 0.40 -0.10 -0.04
cF -0.02 -0.06 -0.05 0.01 -0.03 -0.03
BB 0.07 0.09 0.11 0.02 0.04
B 0.12 0.45 -0.04 0.07
chF 0.49 -0.04 -0.04
CRISPR -0.02 0.07
cM 0.03
cD
1 0.8 0.6 0.4 0.2 0 -0.2 -0.4 -0.6 -0.8 -1

Figure S10 - Correlation analysis of HACK Model fits

These plots measure the correlation between parameter estimates during model optimization, displayed as shades (lower-left triangle, scale on right) with their value (upper-right triangle). Correlation measures the direction (positive or negative number) and magnitude (absolute size of each number) of interactions between variable estimates. The top-row presents deterministic simulations and the bottom row presents stochastic simulations. The left column implements active-homing costs (Table S8, top) and the right column implements co-occurrence costs (Table S8, bottom). In the deterministic simulations, we see strong correlations between cleavage and resistance-generation rates (in males and females), and a looser association between fitness costs and homing rates. These are present but weaker under the co-occurrence model (top, right). All interactions are weaker under stochastic conditions, but they cluster very similarly, indicating that some correlation is still present.

(c{M/F} - male/female cleavage, ch{M/F} - male/female HDR, cr{M/F} - male/female in-frame NHEJ, cD - rate of maternal deposition, CRISPR - cost associated with Cas9/gRNA, B - mosaic cost of out-of-frame NHEJ allele, BB - reproductive cost of compound homozygous out-of-frame NHEJ alleles.)

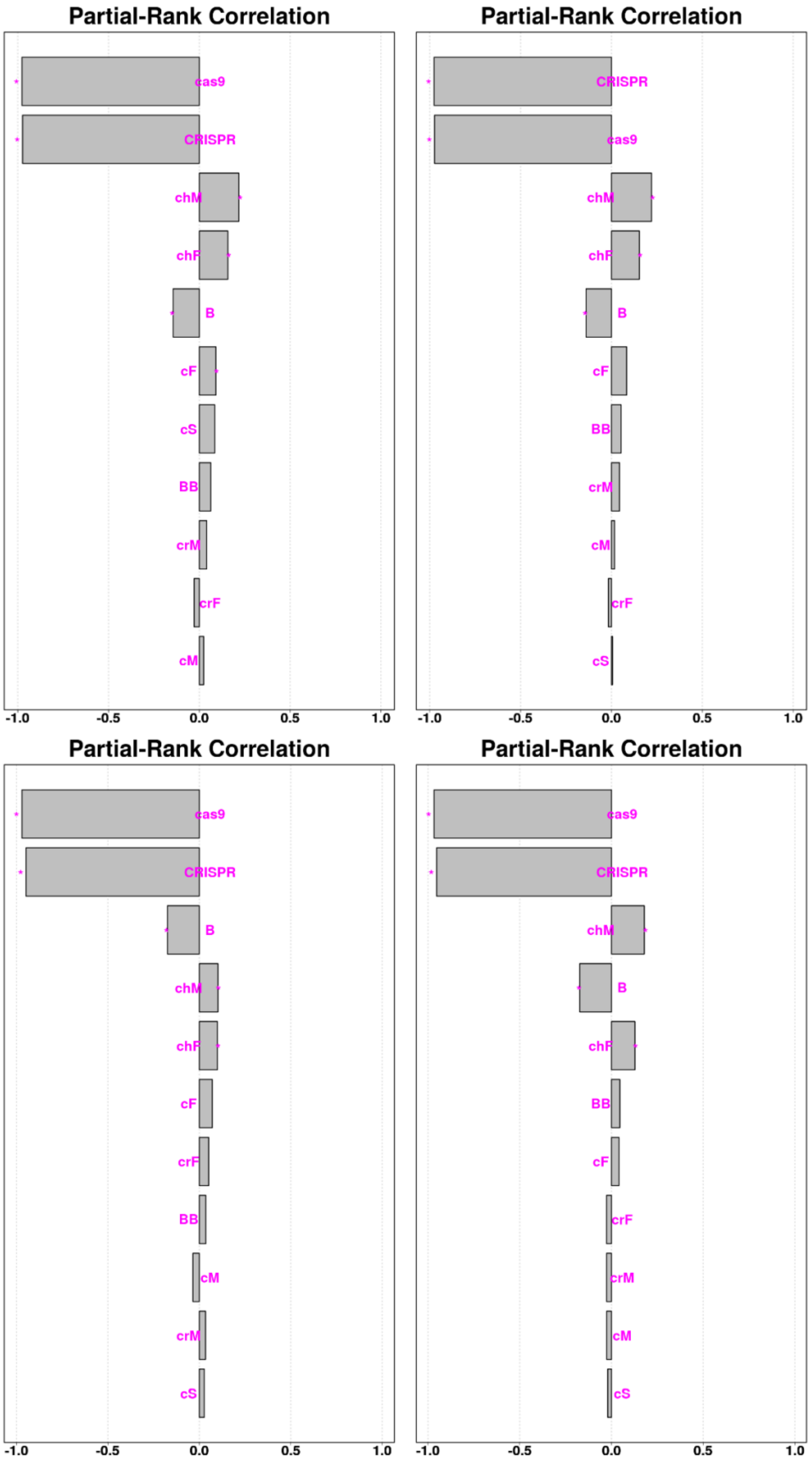
Partial-Rank Correlation
cas9
CRISPR
chM
chF
B
cF
cS
BB
crM
crF
cM
-1.0
-0.5
0.0
0.5
1.0
Partial-Rank Correlation
CRISPR
cas9
chM
chF
B
cF
BB
crM
cM
crF
cS
-1.0
-0.5
0.0
0.5
1.0
Partial-Rank Correlation
cas9
CRISPR
B
chM
chF
cF
crF
BB
cM
crM
cS
-1.0
-0.5
0.0
0.5
1.0
Partial-Rank Correlation
cas9
CRISPR
chM
B
chF
BB
cF
crF
crM
cM
cS
-1.0
-0.5
0.0
0.5
1.0

Figure S11 - Autosomal split-drive PRCC analysis

The PRCC analysis for split-drive with active-homing costs (left column) and co-occurrence costs (right column) was performed using 500 samples chosen using LHS. The top row implements the deterministic model while the bottom row uses the stochastic implementation, taking the average of 50 realizations to calculate the log-likelihood. Asterisks indicate importance at a 0.05 level, calculated using a two-tailed *t*-test. Results from both fitness-cost implementations are very similar. Both indicate a strong, negative correlation between Cas9 expression and Cas9/gRNA-associated fitness costs and log-likelihood, which is a stand-in for population introgression in the cage trial. Homing rates and the fitness cost associated with a single out-of-frame NHEJ allele are next and are of nearly equal importance, though significantly less important than Cas9-associated fitness costs. It is worth acknowledging that this test is limited by the correlation between parameters and the parameter ranges tested.

(c{M/F} - male/female cleavage, ch{M/F} - male/female HDR, cr{M/F} - male/female in-frame NHEJ, cS - rate of shadow drive, CRISPR - cost associated with Cas9/gRNA, B - mosaic cost of out-of-frame NHEJ allele, BB - reproductive cost of compound homozygous out-of-frame NHEJ alleles, cas9 - Cas9 expression cost, independent of cleavage.)

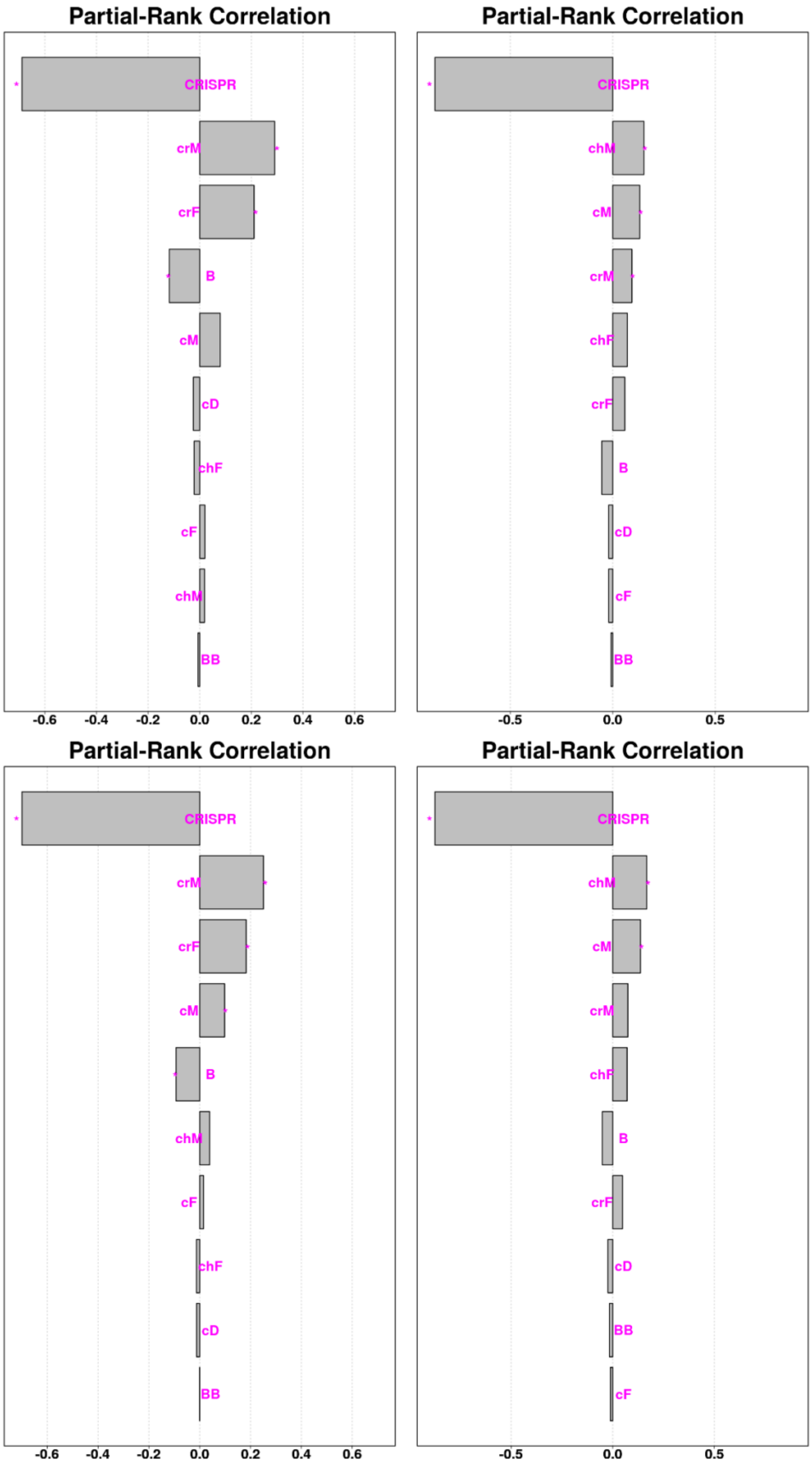

Partial-Rank Correlation
CRISPR
crM
crF
B
cM
cD
chF
cF
chM
BB
-0.6
-0.4
-0.2
0.0
0.2
0.4
0.6
Partial-Rank Correlation
CRISPR
chM
cM
crM
chF
crF
B
cD
cF
BB
-0.5
0.0
0.5
Partial-Rank Correlation
CRISPR
crM
crF
cM
B
chM
cF
chF
cD
BB
-0.6
-0.4
-0.2
0.0
0.2
0.4
0.6
Partial-Rank Correlation
CRISPR
chM
cM
crM
chF
B
crF
cD
BB
cF
-0.5
0.0
0.5

Figure S12 - HACK PRCC analysis

The PRCC analysis for HACK with active-homing costs (left column) and co-occurrence costs (right column) was performed using 500 samples chosen using LHS. The top row implements the deterministic model while the bottom row uses the stochastic implementation, taking the average of 50 realizations to calculate the log-likelihood. Asterisks indicate importance at a 0.05 level, calculated using a two-tailed *t*-test. Similar to the split-drive PRCC (Figure S7), there is a strong, negative correlation between Cas9/gRNA-associated costs and log-likelihood. Other parameters obtain different rankings and correlation measures compared to the split-drive PRCC. There is greater difference between the two Cas9/gRNA-associated models, but the stochastic models are very similar to their deterministic counterparts.

(c{M/F} - male/female cleavage, ch{M/F} - male/female HDR, cr{M/F} - male/female in-frame NHEJ, cD - rate of maternal deposition, CRISPR - cost associated with Cas9/gRNA, B - mosaic cost of out-of-frame NHEJ allele, BB - reproductive cost of compound homozygous out-of-frame NHEJ alleles.)

Full plasmid sequences relevant to the article

***spo11* sGD (p1069E):** Hackable sGD located in *D. melanogaster* chromosome II

white - Backbone

Homology Arms

FauxPAM

gRNA_Hack sequence: to be acted on by the gRNA_Hack cassette located in Cas9Hack plasmids

CTTTACTCGACCTAAACTTTAAACACGTCATAGAATCTTCGTTTGACAAAAACCACATTGTGGCCAAGCTGTGTGACGCGACGC
GCGCTAAAGAATGGCAAACCAAGTCGCGCGAGCGTCGACTCTAGAGGATCCCCGGGTACCGAGCTCGAATTCGTAATCATGG
TCATAGCTGTTTCCTGTGTGAAATTGTTATCCGCTCACAATTCCACACAACATACGAGCCGGAAGCATAAAGTGTAAAGCCTGG
GGTGCCTAATGAGTGAGCTAACTCACATTAATTGCGTTGCGCTCACTGCCCGCTTTCCAGTCGGGAAACCTGTCGTGCCAGCTG
CATTAATGAATCGGCCAACGCGCGGGGAGAGGCGGTTTGCGTATTGGGCGCTCTTCCGCTTCCTCGCTCACTGACTCGCTGCG
CTCGGTCGTTCGGCTGCGGCGAGCGGTATCAGCTCACTCAAAGGCGGTAATACGGTTATCCACAGAATCAGGGGATAACGCA
GGAAAGAACATGTGAGCAAAAGGCCAGCAAAAGGCCAGGAACCGTAAAAAGGCCGCGTTGCTGGCGTTTTTCCATAGGCTCC
GCCCCCCTGACGAGCATCACAAAAATCGACGCTCAAGTCAGAGGTGGCGAAACCCGACAGGACTATAAAGATACCAGGCGTT
TCCCCCTGGAAGCTCCCTCGTGCGCTCTCCTGTTCCGACCCTGCCGCTTACCGGATACCTGTCCGCCTTTCTCCCTTCGGGAAGC
GTGGCGCTTTCTCAATGCTCACGCTGTAGGTATCTCAGTTCGGTGTAGGTCGTTCGCTCCAAGCTGGGCTGTGTGCACGAACCC
CCCGTTCAGCCCGACCGCTGCGCCTTATCCGGTAACTATCGTCTTGAGTCCAACCCGGTAAGACACGACTTATCGCCACTGGCA
GCAGCCACTGGTAACAGGATTAGCAGAGCGAGGTATGTAGGCGGTGCTACAGAGTTCTTGAAGTGGTGGCCTAACTACGGCT
ACACTAGAAGGACAGTATTTGGTATCTGCGCTCTGCTGAAGCCAGTTACCTTCGGAAAAAGAGTTGGTAGCTCTTGATCCGGC
AAACAAACCACCGCTGGTAGCGGTGGTTTTTTTGTTTGCAAGCAGCAGATTACGCGCAGAAAAAAAGGATCTCAAGAAGATCC
TTTGATCTTTTCTACGGGGTCTGACGCTCAGTGGAACGAAAACTCACGTTAAGGGATTTTGGTCATGAGATTATCAAAAAGGAT
CTTCACCTAGATCCTTTTAAATTAAAAATGAAGTTTTAAATCAATCTAAAGTATATATGAGTAAACTTGGTCTGACAGTTACCAA
TGCTTAATCAGTGAGGCACCTATCTCAGCGATCTGTCTATTTCGTTCATCCATAGTTGCCTGACTCCCCGTCGTGTAGATAACTA
CGATACGGGAGGGCTTACCATCTGGCCCCAGTGCTGCAATGATACCGCGAGACCCACGCTCACCGGCTCCAGATTTATCAGCA
ATAAACCAGCCAGCCGGAAGGGCCGAGCGCAGAAGTGGTCCTGCAACTTTATCCGCCTCCATCCAGTCTATTAATTGTTGCCG
GGAAGCTAGAGTAAGTAGTTCGCCAGTTAATAGTTTGCGCAACGTTGTTGCCATTGCTACAGGCATCGTGGTGTCACGCTCGT
CGTTTGGTATGGCTTCATTCAGCTCCGGTTCCCAACGATCAAGGCGAGTTACATGATCCCCCATGTTGTGCAAAAAAGCGGTTA
GCTCCTTCGGTCCTCCGATCGTTGTCAGAAGTAAGTTGGCCGCAGTGTTATCACTCATGGTTATGGCAGCACTGCATAATTCTCT
TACTGTCATGCCATCCGTAAGATGCTTTTCTGTGACTGGTGAGTACTCAACCAAGTCATTCTGAGAATAGTGTATGCGGCGACC
GAGTTGCTCTTGCCCGGCGTCAATACGGGATAATACCGCGCCACATAGCAGAACTTTAAAAGTGCTCATCATTGGAAAACGTT
CTTCGGGGCGAAAACTCTCAAGGATCTTACCGCTGTTGAGATCCAGTTCGATGTAACCCACTCGTGCACCCAACTGATCTTCAG
CATCTTTTACTTTCACCAGCGTTTCTGGGTGAGCAAAAACAGGAAGGCAAAATGCCGCAAAAAAGGGAATAAGGGCGACACG
GAAATGTTGAATACTCATACTCTTCCTTTTTCAATATTATTGAAGCATTTATCAGGGTTATTGTCTCATGAGCGGATACATATTTG
AATGTATTTAGAAAAATAAACAAATAGGGGTTCCGCGCACATTTCCCCGAAAAGTGCCACCTGACGTCTAAGAAACCATTATTA
TCATGACATTAACCTATAAAAATAGGCGTATCACGAGGCCCTTTCGTCTCGCGCGTTTCGGTGATGACGGTGAAAACCTCTGAC
ACATGCAGCTCCCGGAGACGGTCACAGCTTGTCTGTAAGCGGATGCCGGGAGCAGACAAGCCCGTCAGGGCGCGTCAGCGG
GTGTTGGCGGGTGTCGGGGCTGGCTTAACTATGCGGCATCAGAGCAGATTGTACTGAGAGTGCACCATATGCGGTGTGAAAT
ACCGCACAGATGCGTAAGGAGAAAATACCGCATCAGGCGCCATTCGCCATTCAGGCTGCGCAACTGTTGGGAAGGGCGATCG
GTGCGGGCCTCTTCGCTATTACGCCAGCTGGCGAAAGGGGGATGTGCTGCAAGGCGATTAAGTTGGGTAACGCCAGGGTTTT
CCCAGTCACGACGTTGTAAAACGACGGCCAGTGCCAAGCTTTGTTTAAAATATAACAAAATTGTGATCCCACAAAATGAAGTG
GGGCAAAATCAAATAATTAACTAGTGTCCGTAAACTTGTTGGTCTTCAACTTTTTGAGGAACACGTTGGACGGCAAATCGTGAC
TATAACACAAGTTGATTTAATAATTTTAGCCAACACGTCGGGCTGCGTGTTTTTTGCGCTCTGTGTACACGTTGATTAACTGGTC
GATTAAATAATTTAATTTTTGGTTCTTCTTTAAATCTGTGATGAAATTTTTTAAAATAACTTTAAATTCTTCATTGGTAAAAAATG
CCACGTTTTGCAACTTGTGAGGGTCTAATATGAGGTCAAACTCAGTAGGAGTTTTATCCAAAAAAGAAAACATGATTACGTCTG
TACACGAACGCGTATTAACGCAGAGTGCAAAGTATAAGAGGGTTAAAAAATATATTTTACGCACCATATACGCATCGGGTTGA
TATCGTTAATATGGATCAATTTGAACAGTTGATTAACGTGTCTCTGCTCAAGTCTTTGATCAAAACGCAAATCGACGAAAATGT
GTCGGACAATATCAAGTCGATGAGCGAAAAACTAAAAAGGCTAGAATACGACAATCTCACAGACAGCGTTGAGATATACGGT
ATTCACGACAGCAGGCTGAATAATAAAAAAATTAGAAACTATTATTTAACCCTAGAAAGATAATCATATTGTGACGTACGTTAA

AGATAATCATGCGTAAAATTGACGCATGTGTTTTATCGGTCTGTATATCGAGGTTTATTTATTAATTTGAATAGATATTAAGTTT
TATTATATTTACACTTACATACTAATAATAAATTCAACAAACAATTTATTTATGTTTATTTATTTATTAAAAAAAAACAAAAACTC
AAAATTTCTTCTATAAAGTAACAAAACTTTTAAACATTCTCTCTTTTACAAAAATAAACTTATTTTGTACTTTAAAAACAGTCATG
TTGTATTATAAAATAAGTAATTAGCTTAACTTATACATAATAGAAACAAATTATACTTATTAGTCAGTCAGAAACAACTTTGGCA
CATATCAATATTATGCTCTCGACAAATAACTTTTTTGCATTTTTTGCACGATGCATTTGCCTTTCGCCTTATTTTAGAGGGGCAGT
AAGTACAGTAAGTACGTTTTTTCATTACTGGCTCTTCAGTACTGTCATCTGATGTACCAGGCACTTCATTTGGCAAAATATTAGA
GATATTATCGCGCAAATATCTCTTCAAAGTAGGAGCTTCTAAACGCTTACGCATAAACGATGACGTCAGGCTCATGTAAAGGTT
TCTCATAAATTTTTTGCGACTTTGAACCTTTTCTCCCTTGCTACTGACATTATGGCTGTATATAATAAAAGAATTTATGCAGGCAA
TGTTTATCATTCCGTACAATAATGCCATAGGCCACCTATTCGTCTTCCTACTGCAGGTCATCACAGAACACATTTGGTCTAGCGT
GTCCACTCCGCCTTTAGTTTGATTATAATACATAACCATTTGCGGTTTACCGGTACTTTCGTTGATAGAAGCATCCTCATCACAA
GATGATAATAAGTATACCATCTTAGCTGGCTTCGGTTTATATGAGACGAGAGTAAGGGGTCCGTCAAAACAAAACATCGATGT
TCCCACTGGCCTGGAGCGACTGTTTTTCAGTACTTCCGGTATCTCGCGTTTGTTTGATCGCACGGTTCCCACAATGGTTAATTCG
AGCTCGCCCGGGGTCCTAGGGCGCTCTCTTCATTTGCAACTAGTGTGCGTGCTTAAATTTTTAAAAAACAGTTGTTTTCACCGA
AAGGGAATTTTCATTTCGATGGTATATTTTTAGCCAGCAAGCTCCTTTCGGAAATTATTTATAACCCCCCTCGAAATGTTTCCAC
GCAACGATTTACGCGATAAACTTTTACAAAATCAGATATAAGTAAGTGCAAAGACCACTTGATTTTCGTATAGTATGTAAGTCC
ACGAATTGCTTACGTATTTATGAAATATTACTGTGCGTAGGTCGGTGGGCGTGTTTCAACGTTTTAGCCCACAGTTTTCACTCAT
TTTCCTGCGGGGTTTCCGCTTGTGACAAAGGCATTCAAAGAACCAACAAGAAAATCCCGGGGGCGTGGCACCACCCATGCAAT
TAAATTAGACCAAAAAAGTGCTTACACTTTGTATTCTAGAGAATGTTTCAAATCATTGAACATAAACAGAAGAATTCATGCGAA
TTTAGAACTAATGGAAATTGGGAGCAAGGATACATAAAGTTGGAGGAATTCAAAAATGTAATTGTTTAAAATTTAAGCGTTAG
TAACCAAGTTCTTGTTAAAATATATGCCGCTTAATTAAGTTTAAATCTGTTATTCACAAGTAAAATTATATTTATTATTAATTTCTC
TTAATTTGGATATTAAAAAACCTCATTTTGTAGATTGCAAGAGTACGAAATCAAGAAGACGTACATATTTGTTGTTAAGAATCC
GTTCCTAATATAAGTAAATTGCTAGAACAATGGATGAATTTTCGGAGAATATAGAAAGAATTGCCCTCGAGTTGCTGAGCAAC
TTGGTTCATGGCAATGCCACTCTTAGTGTTCCCCGAAATTCATCCGGAAACGTGATCTCGAAATATCGACGAGTCAGCTATAAT
AATCGCGGAAGTCGCCATAGCTTTTGCGTGCTGATTTACATGCTCTCCCGGGTGCACCGATTGCAAGTCCGCGGAGGAAGTTT
CACCGTCCGTGGACTGTATTACGATAACCCATTGCTCGTGCGCAGCCAAAGCCGCATCGCGGAGGCTCGCCTGGACGTGTGCC
GCATGTTGCGCACCAGCCCGCTGTCCCTGGGAATCCTCGCGGCTAGCAAAGGATTGGTCGCCGGAGATCTGCGCTTGCTCATG
ACGAATGGCGATGTGTTGGATTCCTCCCTGTACGGCGGCCCCTTGACCCTGCCGACCGACCCGGAAAAAAATTGACCGTATTGA
GACCTTGGCCGAGTTCGTCCTGATTGTGGAGAAAGAAAGCGTGTTCGAAAGCCTGCTGAGCCGCAACGTGTTCGGCACCTTCG
AGCGCCGTTTTATTCTGATCACCGGCAAGGGCTATCCGGACTGTTGCACGCGCCGCATCGTGCACCGCCTGACGGAAGAAAAT
CAGTTGGCCGCGTATATCCTGGTGGATGCGGACCCCTTCGGAGTGGAAATCATGTTGGTGTACCGTCACGGCAGCAAGAGTAT
GTCCTTCAGCTCCCAGGGCCTGACGACCCCAGCCCTCCGCTGGATCGGCTTGCATCCAAGCGAAATCCCGGCCCTGGGAACCG
GCGCCGTGGCTTTGGTGGCTGGAGATAATAAAAAGATTAACGATCTGCTGGCGCGTCATGACCTGGAACCCGGCGTCCGCCA
AGAGTTGAGGATGTTGCAAGATGTGCAACTCAAAGCTGAGATTGAAAGCGTGATTGATTTTCTCACGGATGATTACATCCCCA
ACAAGATTAACCGCAATCTGTTCCTGTAAAATGAATCGTTTTTAAAATAACAAATCAATTGTTTTATAATATTCGTACGATTCTTT
GATTATGTAATAAAATGTGATCATTAGGAAGATTACGAAAAATATAAAAAATATGAGTTCTGTGTGTATAACAAATGCTGTAAA
CGCCACAATTGTGTTTGTTGCAAATAAACCCATGATTATTTGATTAAAATTGTTGTTTTCTTTGTTCATAGACAATAGTGTGTTTT
GCCTAAACGTGTACTGCATAAACTCCATGCGAGTGTATAGCGAGCTAGTGGCTAACGCTTGCCCCACCAAAGTAGATTCGTCA
AAATCCTCAATTTCATCACCCTCCTCCAAGTTTAACATTTGGCCGTCGGAATTAACTTCTAAAGATGCCACATAATCTAATAAAT
GAAATAGAGATTCAAACGTGGCGTCATCGTCCGTTTCGACCATTTCCGAAAAGAACTCGGGCATAAACTCTATGATTTCTCTGG
ACGTGGTGTTGTCGAAACTCTCAAAGTACGCAGTCAGGAACGTGCGCGACATGTCGTCGGGAAACTCGCGCGGAAACATGTT
GTTGTAACCGAACGGGTCCCATAGCGCCAAAACCAAATCTGCCAGCGTCAATAGAATGAGCACGATGCCGACAATGGAGCTG
GCTTGGATAGCGATTCGAGTTAACGGTTTTTTGCTCACCTGTGATTGCTCCTACTCAAATACAAAAACATCAAATTTTCTGTCAA
TAAAGCATATTTATTTATATTTATTTTACAGGAAAGAATTCCTTTTAAAGTGTATTTTAACCTATAATGAAAAACGATTAAAAAA
AATACATAAAATAATTCGAAAATTTTTGAATAGCCCAGGTTGATAAAAATTCATTTCATACGTTTTATAACTTATGCCCCTAAGT
ATTTTTTGACCATAGTGTTTCAATTCTACATTAATTTTACAGAGTAGAATGAAACGCCACCTACTCAGCCAAGAGGCGAAAAGG
TTAGCTCGCCAAGCAGAGAGGGCGCCAGTGCTCACTACTTTTTATAATTCTCAACTTCTTTTTCCAGACTCAGTTCGTATATATA
GACCTATTTTCAATTTAACGTCGATTGTCATAGTATAGTCCAGTTTTAGAGCTAGAAATAGCAAGTTAAAATAAGGCTAGTCCG
TTATCAACTTGAAAAAGTGGCACCGAGTCGGTGCTTTTTTGCCTACCTGGAGCCTGAGAGTTGTTCAATAAAATAAAAATGTTT
CGTTTTTTTGCTTTCGCCAGTATTTATTATTTTTCATCAATATGTATTCAATTTGGTATGTATTTAGTAATTGTAATATATAGACAA
TGGTTTTCCGTTGACGTACATACATCTGACGTGTGTTTATTTAGACATAATAGTTATGTTTTCACATCTTTTTAATGTTCGCTTAA
TGCGTATGCATTCTAGACAATTGTGCTCGGCAACAGTATATTTGTGGTGTGCCAACCAACAACCTGCAGGAGCTCCAGCTTTTG
TAATTCGAGCTCGCCCGGGGATCTAATTCAATTAGAGACTAATTCAATTAGAGCTAATTCAATTAGGATCCAAGCTTATCGATTT
CGAACCCTCGACCGCCGGAGTATAAATAGAGGCGCTTCGTCTACGGAGCGACAATTCAATTCAAACAAGCAAAGTGAACACGT

CGCTAAGCGAAAGCTAAGCAAATAAACAAGCGCAGCTGAACAAGCTAAACAATCGGGGTACCGCTAGAGTCGACGGTACGAT
CCACCGGTCGCCACCATGGTGAGCAAGGGCGAGGAGGTCATCAAAGAGTTCATGCGCTTCAAGGTGCGCATGGAGGGCTCCA
TGAACGGCCACGAGTTCGAGATCGAGGGCGAGGGCGAGGGCCGCCCCTACGAGGGCACCCAGACCGCCAAGCTGAAGGTGA
CCAAGGGCGGCCCCCTGCCCTTCGCCTGGGACATCCTGTCCCCCCAGTTCATGTACGGCTCCAAGGCGTACGTGAAGCACCCC
GCCGACATCCCCGATTACAAGAAGCTGTCCTTCCCCGAGGGCTTCAAGTGGGAGCGCGTGATGAACTTCGAGGACGGCGGTC
TGGTGACCGTGACCCAGGACTCCTCCCTGCAGGACGGCACGCTGATCTACAAGGTGAAGATGCGCGGCACCAACTTCCCCCCC
GACGGCCCCGTAATGCAGAAGAAGACCATGGGCTGGGAGGCCTCCACCGAGCGCCTGTACCCCCGCGACGGCGTGCTGAAG
GGCGAGATCCACCAGGCCCTGAAGCTGAAGGACGGCGGCCACTACCTGGTGGAGTTCAAGACCATCTACATGGCCAAGAAGC
CCGTGCAACTGCCCGGCTACTACTACGTGGACACCAAGCTGGACATCACCTCCCACAACGAGGACTACACCATCGTGGAACAG
TACGAGCGCTCCGAGGGCCGCCACCACCTGTTCCTGGGGCATGGCACCGGCAGCACCGGCAGCGGCAGCTCCGGCACCGCCT
CCTCCGAGGACAACAACATGGCCGTTATCAAGGAATTTATGCGCTTCAAAGTTAGGATGGAGGGATCCATGAACGGACATGA
GTTCGAGATCGAGGGAGAGGGCGAGGGACGCCCGTATGAAGGCACACAAACAGCCAAACTCAAGGTCACCAAGGGCGGACC
ACTGCCCTTCGCCTGGGATATCCTGAGTCCCCAGTTTATGTACGGCAGCAAGGCCTACGTTAAGCACCCCGCTGACATACCGGA
CTACAAAAAGCTGTCCTTTCCGGAAGGCTTCAAGTGGGAGCGCGTGATGAATTTCGAAGACGGAGGACTGGTCACTGTGACC
CAAGATAGCAGTTTGCAGGACGGTACACTGATCTATAAGGTTAAAATGCGCGGCACTAACTTTCCGCCAGATGGCCCAGTGAT
GCAGAAGAAGACCATGGGTTGGGAGGCATCCACCGAACGTCTGTACCCTCGAGACGGAGTGCTCAAGGGCGAGATCCATCA
GGCCCTCAAACTGAAAGATGGTGGTCACTACCTGGTCGAATTTAAGACCATTTACATGGCCAAGAAGCCGGTTCAGCTGCCCG
GATATTATTATGTGGATACGAAACTGGATATAACTTCGCATAACGAAGACTACACCATTGTCGAGCAGTATGAGCGCAGCGAA
GGCCGACATCACCTGTTCCTCTACGGCATGGACGAGCTGTACAAGTAGGGTCTTTCGCCCGCCGCGAAAGCTCTTCAAAGGCA
GCAACCAGCAGCGACCAACAAGCATCCATCGAGCTACCCAACAACCTCGGCTCGGACAGTGATAGACAAAAGCAGCGAACCC
ATCGCACAACAATTATCATCCAACTCAGATTCACAGCAGATAATCAAAGGCAACCTCCGGTTGTCGGTGCTCATCCTTCATGGC
CATTTCATCGGCAGCGGTATAGCGGATTTTTACTTTGAAGAACTAATCGTAAGAGTCGTGGCTGTGCTCCATGTCGAGTAGCAA
TCAAATGTATATGAGGAGCTTTTAACCCTAGTCAGTGAATTGAAAGCCAAATATATCTTCCATTAAAACTATTAAATATTTTAAA
TAAATACATTTTTCTATTACCCGCCCCCCCGTGTACTGAAAAGCCCTGGAGGTGCGAACCACGTTTGGTTGGTAACAGCTTTAA
ATTCCTTTTAATTGACATTAAATTAATTGTTAACATTGGTTTTGCATTAGCCTAGCTAGTTAAATGAAAATACAGCGAGGATACT
TGGTACCGGGACTTAGGCGATTTGGGATACAATCGATTGAACGTTGAAAATCTGGCAGCCGGTCACTGCACGCCGTAGGTCA
GGGTGGTCACGAGGGTGGGCCAGGGCACGGGCAGCTTGCCGGTGGTGCAGATGAACTTCAGGGTCAGCTTGCCGTAGGTGG
CATCGCCCTCGCCCTCGCCGGACACGCTGAACTTGTGGCCGTTTACGTCGCCGTCCAGCTCGACCAGGATGGGCACCACCCCG
GTGAACAGCTCCTCGCCCTTGCTCACCATCACCAGAGACAGGTTGCGGCGGCGGTTGGATGGCGTGGGCGCGTTGGCGTTGT
TGGACCGGCTCATGTTGTGTCGCTGTAACAGATGCTGTTCAACTGTGTTTACCAGATCGTTGCGGGCTGTATTTATAGGCGCGA
TAAGCGGGACGGGCGCCTCGTGTCCGGTCACGCGCATGAGATAACGCGCGGCTGATATGGAGGCGCGTCCTGTTCCGATAAG
GAGTTGCGTCCGGCTGCGGTTAGCAACACAGGAAGCTGGCGTCCTGTCACGATAAGACAACACTCGTCCGGTCCGATAATGTG
ATTCGTACGTGACAGGACGCGACCCGATAAGGCCGGCCTACGTGACTGCCGACACGTACTTTTTTGCACTGCAAAAAGGTTCA
ATGTGTGGTAGTGTATTTGGAGCGTATACAACGGTGTAGACTATTTATGTAAAATAGTCTACGAAACGTAGAGTTTGTACTATG
TATGGGCCCGCGTGCAAAAGCGTGTTTTTTTGCAGTGCAAAAAAGTTGGTGGTGGGGAGGCCACCGAGTATGACTATACTATG
ACAATCCTCTGCTAGTCCGGTCGCAGTCCAGGATTGCCGAAGCCAGGCTAGATGTCTGTCGTATGCTGAGGACATCCCCCCTAA
GCTTGGGCATACTGGCCGCCTCCAAGGGCCTGGTGGCAGGTGAGTTTATCTCGAAATCCTGTTCAAAGGCTCCCTGCTCATCC
GGTTACTCTTGCAGGCGACTTAAGGCTGCTGATGACCAACGGAGACGTTCTGGACAGCAGCTTGTATGGTGGACCTCTGACAT
TGCCCACGGATCCCGAGAAGATAGATCGAATCGAAACGCTGGCGGAATTTGTGCTGATCGTTGAAAAGGAGTCGGTGTTTGA
GAGTCTCTTATCCAGAAATGTATTTGGTACTTTTGAACGACGCTTCATCCTTATAACTGGAAAAGGATACCCCGATTGCTGTACC
CGGAGGATTGTCCATCGGCTCACCGAGGAGAACCAACTGGCGGCCTACATTCTCGTGGACGCCGATCCATTTGGCGTCGAGAT
AATGCTAGTCTATCGCCATGGCTCCAAGTCCATGAGTTTTTCCAGCCAAGGACTAACCACACCTGCGCTGCGTTGGATTGGTCT
ACACCCCTCGGAGATTCCCGCACTCGGCACTGGAGCGGTTGCCCTGGTTGCCGGCGACAACAAGAAAATCAATGACCTCCTCG
CCCGCCACGATTTGGAGCCGGGAGTGCGGCAGGAACTGCGCATGCTGCAGGACGTTCAGCTGAAGGCCGAAATCGAGAGTG
TCATCGACTTCCTGACCGACGACTATATACCAAATAAAATCAATCGGAACTTGTTTTTGTAGAGTTCAGTGGTAAACCCCATAA
GCCAAATATTATTACTCTTGAAATATTATTAGTTAGTTTAAAATATGTTGTAGGTATAGTAATATAGTAATTAGTTTGTAAAAAA
CGTATCTCTATAAAGATACCATAAGACTCCTAAAAATAGTACTGTAAAAGTTAGTTCGCGTAATCAGCTTTCCGGCGCGCCCAT
ACTCGGTGGCCTCCCCACCACCAACTTTTTTGCACTGCAAAAAAACACGCTTTTGCACGCGGGCCCATACATAGTACAAACTCT
ACGTTTCGTAGACTATTTTACATAAATAGTCTACACCGTTGTATACGCTCCAAATACACTACCACACATTGAACCTTTTTGCAGT
GCAAAAAAGTACGTGTCGGCAGTCACGTAGGCCGGCCTTATCGGGTCGCGTCCTGTCACGTACGAATCACATTATCGGACCGG
ACGAGTGTTGTCTTATCGTGACAGGACGCCAGCTTCCTGTGTTGCTAACCGCAGCCGGACGCAACTCCTTATCGGAACAGGAC
GCGCCTCCATATCAGCCGCGCGTTATCTCATGCGCGTGACCGGACACGAGGCGCCCGTCCCGCTTATCGCGCCTATAAATACA
GCCCGCAACGATCTGGTAAACACAGTTGAACAGCATCTGTTACAGCGACACAACATGAGCCGGTCCAACAACGCCAACGCGCC

CACGCCATCCAACCGCCGCCGCAACCTGTCTCTGGTGATGGTGCGCTCCTCCAAGAACGTCATCAAGGAGTTCATGCGCTTCAA
GGTGCGCATGGAGGGCACCGTGAACGGCCACGAGTTCGAGATCGAGGGCGAGGGCGAGGGCCGCCCCTACGAGGGCCACA
ACACCGTGAAGCTGAAGGTGACCAAGGGCGGCCCCCTGCCCTTCGCCTGGGACATCCTGTCCCCCCAGTTCCAGTACGGCTCC
AAGGTGTACGTGAAGCACCCCGCCGACATCCCCGACTACAAGAAGCTGTCCTTCCCCGAGGGCTTCAAGTGGGAGCGCGTGA
TGAACTTCGAGGACGGCGGCGTGGTGACCGTGACCCAGGACTCCTCCCTGCAGGACGGCTGCTTCATCTACAAGGTGAAGTTC
ATCGGCGTGAACTTCCCCTCCGACGGCCCCGTAATGCAGAAGAAGACCATGGGCTGGGAGGCCTCCACCGAGCGCCTGTACC
CCCGCGACGGCGTGCTGAAGGGCGAGATCCACAAGGCCCTGAAGCTGAAGGACGGCGGCCACTACCTGGTGGAGTTCAAGT
CCATCTACATGGCCAAGAAGCCCGTGCAGCTGCCCGGCTACTACTACGTGGACTCCAAGCTGGACATCACCTCCCACAACGAG
GACTACACCATCGTGGAGCAGTACGAGCGCACCGAGGGCCGCCACCACCTGTTCCTGTAGGACTCTAGATCATAATCAGCCAT
ACCACATTTGTAGAGGTTTTACTTGCTTTAAAAAACCTCCCACACCTCCCCCTGAACCTGAAACATAAAATGAATGCAATTGTTG
TTGTTAACTTGTTTATTGCAGCTTATAATGGTTACAAATAAAGCAATAGCATCACAAATTTCACAAATAAAGCATTTTTTTCACTG
CATTCTAGTTGTGGTTTGTCCAAACTCATCAATGTATCTTAAAGCTTCTAGGCCGGCCGATCTCGGATCTGACAATGTTCAGTGC
AGAGACTCGGCTACGCCTCGTGGACTTTGAAGTTGACCAACAATGTTTATTCTTACCTCTAATAGTCCTCTGTGGCAAGGTCAA
GATTCTGTTAGAAGCCAATGAAGAACCTGGTTGTTCAATAACATTTTGTTCGTCTAATATTTCACTACCGCTTGACGTTGGCTGC
ACTTCATGTACCTCATCTATAAACGCTTCTTCTGTATCGCTCTGGACGTCATCTTCACTTACGTGATCTGATATTTCACTGTCAGA
ATCCTCACCAACAAGCTCGTCATCGCTTTGCAGAAGAGCAGAGAGGATATGCTCATCGTCTAAAGAACTACCCATTTTATTATA
TATTAGTCACGATATCTATAACAAGAAAATATATATATAATAAGTTATCACGTAAGTAGAACATGAAATAACAATATAATTATC
GTATGAGTTAAATCTTAAAAGTCACGTAAAAGATAATCATGCGTCATTTTGACTCACGCGGTCGTTATAGTTCAAAATCAGTGA
CACTTACCGCATTGACAAGCACGCCTCACGGGAGCTCCAAGCGGCGACTGAGATGTCCTAAATGCACAGCGACGGATTCGCGC
TATTTAGAAAGAGAGAGCAATATTTCAAGAATGCATGCGTCAATTTTACGCAGACTATCTTTCTAGGGTTAAAAAAGATTTGCG

pGT12_HACK: *vasa*-Hack that inserts into AttP sites

white - Backbone
AttB site
Homology Arms
vasa-Cas9 cassette
gRNA_HACK expression cassette: **gRNA_HACK (Present only in sGD transgene)**

GATCTCGGATCTGACAATGTTCAGTGCAGAGACTCGGCTACGCCTCGTGGACTTTGAAGTTGACCAACAATGTTTATTCTTACC
TCTAATAGTCCTCTGTGGCAAGGTCAAGATTCTGTTAGAAGCCAATGAAGAACCTGGTTGTTCAATAACATTTTGTTCGTCTAAT
ATTTCACTACCGCTTGACGTTGGCTGCACTTCATGTACCTCATCTATAAACGCTTCTTCTGTATCGCTCTGGACGTCATCTTCACT
TACGTGATCTGATATTTCACTGTCAGAATCCTCACCAACAAGCTCGTCATCGCTTTGCAGAAGAGCAGAGAGGATATGCTCATC
GTCTAAAGAACTACCCATTTTATTATATATTAGTCACGATATCTATAACAAGAAAATATATATATAATAAGTTATCACGTAAGTA
GAACATGAAATAACAATATAATTATCGTATGAGTTAAATCTTAAAAGTCACGTAAAAGATAATCATGCGTCATTTTGACTCACG
CGGTCGTTATAGTTCAAAATCAGTGACACTTACCGCATTGACAAGCACGCCTCACGGGAGCTCCAAGCGGCGACTGAGATGTC
CTAAATGCACAGCGACGGATTCGCGCTATTTAGAAAGAGAGAGCAATATTTCAAGAATGCATGCGTCAATTTTACGCAGACTA
TCTTTCTAGGGTTAAAAAAGATTTGCGCTTTACTCGACCTAAACTTTAAACACGTCATAGAATCTTCGTTTGACAAAAACCACAT
TGTGGCCAAGCTGTGTGACGCGACGCGCGCTAAAGAATGGCAAACCAAGTCGCGCGAGCGTCGACTCTAGAGGATCCCCGGG
TACCGAGCTCGAATTCGTAATCATGGTCATAGCTGTTTCCTGTGTGAAATTGTTATCCGCTCACAATTCCACACAACATACGAGC
CGGAAGCATAAAGTGTAAAGCCTGGGGTGCCTAATGAGTGAGCTAACTCACATTAATTGCGTTGCGCTCACTGCCCGCTTTCC
AGTCGGGAAACCTGTCGTGCCAGCTGCATTAATGAATCGGCCAACGCGCGGGGAGAGGCGGTTTGCGTATTGGGCGCTCTTC
CGCTTCCTCGCTCACTGACTCGCTGCGCTCGGTCGTTCGGCTGCGGCGAGCGGTATCAGCTCACTCAAAGGCGGTAATACGGT
TATCCACAGAATCAGGGGATAACGCAGGAAAGAACATGTGAGCAAAAGGCCAGCAAAAGGCCAGGAACCGTAAAAAGGCCG
CGTTGCTGGCGTTTTTCCATAGGCTCCGCCCCCCTGACGAGCATCACAAAAATCGACGCTCAAGTCAGAGGTGGCGAAACCCG
ACAGGACTATAAAGATACCAGGCGTTTCCCCCTGGAAGCTCCCTCGTGCGCTCTCCTGTTCCGACCCTGCCGCTTACCGGATAC
CTGTCCGCCTTTCTCCCTTCGGGAAGCGTGGCGCTTTCTCAATGCTCACGCTGTAGGTATCTCAGTTCGGTGTAGGTCGTTCGCT
CCAAGCTGGGCTGTGTGCACGAACCCCCCGTTCAGCCCGACCGCTGCGCCTTATCCGGTAACTATCGTCTTGAGTCCAACCCGG
TAAGACACGACTTATCGCCACTGGCAGCAGCCACTGGTAACAGGATTAGCAGAGCGAGGTATGTAGGCGGTGCTACAGAGTT
CTTGAAGTGGTGGCCTAACTACGGCTACACTAGAAGGACAGTATTTGGTATCTGCGCTCTGCTGAAGCCAGTTACCTTCGGAA

AAAGAGTTGGTAGCTCTTGATCCGGCAAACAAACCACCGCTGGTAGCGGTGGTTTTTTTGTTTGCAAGCAGCAGATTACGCGC
AGAAAAAAAGGATCTCAAGAAGATCCTTTGATCTTTTCTACGGGGTCTGACGCTCAGTGGAACGAAAACTCACGTTAAGGGAT
TTTGGTCATGAGATTATCAAAAAGGATCTTCACCTAGATCCTTTTAAATTAAAAATGAAGTTTTAAATCAATCTAAAGTATATAT
GAGTAAACTTGGTCTGACAGTTACCAATGCTTAATCAGTGAGGCACCTATCTCAGCGATCTGTCTATTTCGTTCATCCATAGTTG
CCTGACTCCCCGTCGTGTAGATAACTACGATACGGGAGGGCTTACCATCTGGCCCCAGTGCTGCAATGATACCGCGAGACCCA
CGCTCACCGGCTCCAGATTTATCAGCAATAAACCAGCCAGCCGGAAGGGCCGAGCGCAGAAGTGGTCCTGCAACTTTATCCGC
CTCCATCCAGTCTATTAATTGTTGCCGGGAAGCTAGAGTAAGTAGTTCGCCAGTTAATAGTTTGCGCAACGTTGTTGCCATTGC
TACAGGCATCGTGGTGTCACGCTCGTCGTTTGGTATGGCTTCATTCAGCTCCGGTTCCCAACGATCAAGGCGAGTTACATGATC
CCCCATGTTGTGCAAAAAAGCGGTTAGCTCCTTCGGTCCTCCGATCGTTGTCAGAAGTAAGTTGGCCGCAGTGTTATCACTCAT
GGTTATGGCAGCACTGCATAATTCTCTTACTGTCATGCCATCCGTAAGATGCTTTTCTGTGACTGGTGAGTACTCAACCAAGTCA
TTCTGAGAATAGTGTATGCGGCGACCGAGTTGCTCTTGCCCGGCGTCAATACGGGATAATACCGCGCCACATAGCAGAACTTT
AAAAGTGCTCATCATTGGAAAACGTTCTTCGGGGCGAAAACTCTCAAGGATCTTACCGCTGTTGAGATCCAGTTCGATGTAACC
CACTCGTGCACCCAACTGATCTTCAGCATCTTTTACTTTCACCAGCGTTTCTGGGTGAGCAAAAACAGGAAGGCAAAATGCCGC
AAAAAAGGGAATAAGGGCGACACGGAAATGTTGAATACTCATACTCTTCCTTTTTCAATATTATTGAAGCATTTATCAGGGTTA
TTGTCTCATGAGCGGATACATATTTGAATGTATTTAGAAAAATAAACAAATAGGGGTTCCGCGCACATTTCCCCGAAAAGTGCC
ACCTGACGTCTAAGAAACCATTATTATCATGACATTAACCTATAAAAATAGGCGTATCACGAGGCCCTTTCGTCTCGCGCGTTTC
GGTGATGACGGTGAAAACCTCTGACACATGCAGCTCCCGGAGACGGTCACAGCTTGTCTGTAAGCGGATGCCGGGAGCAGAC
AAGCCCGTCAGGGCGCGTCAGCGGGTGTTGGCGGGTGTCGGGGCTGGCTTAACTATGCGGCATCAGAGCAGATTGTACTGA
GAGTGCACCATATGCGGTGTGAAATACCGCACAGATGCGTAAGGAGAAAATACCGCATCAGGCGCCATTCGCCATTCAGGCT
GCGCAACTGTTGGGAAGGGCGATCGGTGCGGGCCTCTTCGCTATTACGCCAGCTGGCGAAAGGGGGATGTGCTGCAAGGCG
ATTAAGTTGGGTAACGCCAGGGTTTTCCCAGTCACGACGTTGTAAAACGACGGCCAGTGCCAAGCTTTGTTTAAAATATAACA
AAATTGTGATCCCACAAAATGAAGTGGGGCAAAATCAAATAATTAACTAGTGTCCGTAAACTTGTTGGTCTTCAACTTTTTGAG
GAACACGTTGGACGGCAAATCGTGACTATAACACAAGTTGATTTAATAATTTTAGCCAACACGTCGGGCTGCGTGTTTTTTGCG
CTCTGTGTACACGTTGATTAACTGGTCGATTAAATAATTTAATTTTTGGTTCTTCTTTAAATCTGTGATGAAATTTTTTAAAATAA
CTTTAAATTCTTCATTGGTAAAAAATGCCACGTTTTGCAACTTGTGAGGGTCTAATATGAGGTCAAACTCAGTAGGAGTTTTATC
CAAAAAAGAAAACATGATTACGTCTGTACACGAACGCGTATTAACGCAGAGTGCAAAGTATAAGAGGGTTAAAAAATATATTT
TACGCACCATATACGCATCGGGTTGATATCGTTAATATGGATCAATTTGAACAGTTGATTAACGTGTCTCTGCTCAAGTCTTTGA
TCAAAACGCAAATCGACGAAAATGTGTCGGACAATATCAAGTCGATGAGCGAAAAACTAAAAAGGCTAGAATACGACAATCT
CACAGACAGCGTTGAGATATACGGTATTCACGACAGCAGGCTGAATAATAAAAAAATTAGAAACTATTATTTAACCCTAGAAA
GATAATCATATTGTGACGTACGTTAAAGATAATCATGCGTAAAATTGACGCATGTGTTTTATCGGTCTGTATATCGAGGTTTATT
TATTAATTTGAATAGATATTAAGTTTTATTATATTTACACTTACATACTAATAATAAATTCAACAAACAATTTATTTATGTTTATTT
ATTTATTAAAAAAAAACAAAAACTCAAAATTTCTTCTATAAAGTAACAAAACTTTTAAACATTCTCTCTTTTACAAAAATAAACTT
ATTTTGTACTTTAAAAACAGTCATGTTGTATTATAAAATAAGTAATTAGCTTAACTTATACATAATAGAAACAAATTATACTTATT
AGTCAGTCAGAAACAACTTTGGCACATATCAATATTATGCTCTCGACAAATAACTTTTTTGCATTTTTTGCACGATGCATTTGCCT
TTCGCCTTATTTTAGAGGGGCAGTAAGTACAGTAAGTACGTTTTTTCATTACTGGCTCTTCAGTACTGTCATCTGATGTACCAGG
CACTTCATTTGGCAAAATATTAGAGATATTATCGCGCAAATATCTCTTCAAAGTAGGAGCTTCTAAACGCTTACGCATAAACGA
TGACGTCAGGCTCATGTAAAGGTTTCTCATAAATTTTTTGCGACTTTGAACCTTTTCTCCCTTGCTACTGACATTATGGCTGTATA
TAATAAAAGAATTTATGCAGGCAATGTTTATCATTCCGTACAATAATGCCATAGGCCACCTATTCGTCTTCCTACTGCAGGTCAT
CACAGAACACATTTGGTCTAGCGTGTCCACTCCGCCTTTAGTTTGATTATAATACATAACCATTTGCGGTTTACCGGTACTTTCG
TTGATAGAAGCATCCTCATCACAAGATGATAATAAGTATACCATCTTAGCTGGCTTCGGTTTATATGAGACGAGAGTAAGGGG
TCCGTCAAAACAAAACATCGATGTTCCCACTGGCCTGGAGCGACTGTTTTTCAGTACTTCCGGTATCTCGCGTTTGTTTGATCGC
ACGGTTCCCACAATGGTTAATTCGAGCTCGCCCGGGGTCCTAGGTCGACGATGTAGGTCACGGTCTCGAAGCCGCGGTGCGG
GTGCCAGGGCGTGCCCTTGGGCTCCCCGGGCGCGTACTCCACCTCACCCATCTGGTCCATCATGATGAACGGGTCGAGGTGGC
GGTAGTTGATCCCGGCGAACGCGCGGCGCACCGGGAAGCCCTCGCCCTCGAAACCGCTGGGCGCGGTGGTCACGGTGAGCA
CGGGACGTGCGACGGCGTCGGCGGGTGCGGATACGCGGGGCAGCGTCAGCGGGTTCTCGACGGTCACGGCGGGCATGTCG
ACCAAGAAGCCGGTTCAGCTGCCCGGATATTATTATGTGGATACGAAACTGGATATAACTTCGCATAACGAAGACTACACCAT
TGTCGAGCAGTATGAGCGCAGCGAAGGCCGACATCACCTGTTCCTCTACGGCATGGACGAGCTGTACAAGTAGGGTCTTTCGC
CCGCCGCGAAAGCTCTTCAAAGGCAGCAACCAGCAGCGACCAACAAGCATCCATCGAGCTACCCAACAACCTCGGCTCGGACA
GTGATAGACAAAAGCAGCGAACCCATCGCACAACAATTATCATCCAACTCAGATTCACAGCAGATAATCAAAGGCAACCTCCG
GTTGTCGGTGCTCATCCTTCATGGCCATTTCATCGGCAGCGGTATAGCGGATTTTTACTTTGAAGAACTAATCGTAAGAGTCGT
GGCTGTGCTCCATGTCGAGTAGCAATCAAATGTATATGAGGAGCTTTTAACCCTAGTCAGTGAATTGAAAGCCAAATATATCTT
CCATTAAAACTATTAAATATTTTAAATAAATACATTTTTCTATTACCCGCCCCCCCGTGTACTGAAAAGCCCTGGAGGTGCGAAC
CACGTTTGGTTGGTAACAGCTTTAAATTCCTTTTAATTGACATTAAATTAATTGTTAACATTGGTTTTGCATTAGCCTAGCTAGTT

AAATGAAAATACAGCGAGGATACTTGGTACCGGGACTTAGGCGATTTGGGATACAATCGATTGAACGTTGAAAATCTGGCAG
CCGGTCCTGCAGCTGGTTGTAGGTGCAGTTGCGCTTCTTGCACTTGGCGCACTTGAGGAGATCGGTCTTGGTGCCCTGCACGG
TGGCCAGCTGGGCATCGTTGATGGCCTCCTTGACGAACTTCTCGCGCAGCTTCTTCATCTCGTCGCTGGCCATCTCCTCCGGCGT
CATTTTGGCCAGCTGCTTGGCAGTGACGGCGCCGCACATAAAGTTGCCGCGCAATCCAGGATTCTTGGGGTCCTTCAGATTGG
CCACGCGTGACCTAATGCGATTCTTGTACTTCATATCCGTGTTATTGAACTCGGAGTAAATGGCATCCTCCAGTTCGGCGGCCA
TTTCCTCCGGCTCACCGCATCCCTCGGGCACTTCGCCAATCTTCAGTGCGGTGGCCAGCATTTCGCGGCATTTGATGCGGACCG
CATCTGTCATGCCACCGGAGGGAAACGAGGTCTGTGACGAGGATGTCGAGCCTTTCTTCTCTTTATCCTTGGATGAGCTGGAT
GAACTGGACTTATCCTTGCCGGAGATTGACGACGACGACTTGGCGGCACTGGTGGACTTCGAGGCACTGCTGTTGTTGGAGG
AGCCCTCCTTAGCGGAGCTGTTGTTCGGTGTGGTTGGCGCCGGGCTGGCCAGGAAGCGTTTCCAGTTCTTGATCAGCGTCTTG
GCCAGAGCGATCACCTCGTCGTCCTTGCTACTCTTGCGCAGCTCGTTTACGGTCATGCCGATGCGCGTTTTGGTCAGAATGTCG
AGATTGATGTTAAGCGTTTGCAGGGCCTTCAGCAGGTCCAGAGCCTGATCCTGTCCCTGTACGGTGGATATATGTATATTTGTG
ACATGATGCGCGGCACGTTAGCTAATCCCGATACTTACTGTGCCGTCGCTGGCCATCTTGCTCATCTTCTTTTGGATTCGAAACA
CTTCCTCCTCCACGCTCATTTTGGCCCACTTAATTTGATCAGAGGTTAGGTTAACTGGCGTCGGCTGTCGGCTTTAATCGGTGCT
GCGGGTCTGCGACAGTTGTTTATGCGGAAGCCGGGCTATTTCTTTTTTTCCCTCCAATAAATGTTGTTTACTATTTTTTTCGTTTC
TGTATTCGCAGAACAAGTTGACAGAACACCCTAACGCGTTTTAGTGTTACCGGGCGGTGGCTTGTCAAGAAATACCTTCCCATG
GCTAATACAATTCCCAACTGGAAGTAGTTTGGGTTTCAGAAGTCAACAGTGTTACCGTATTTTAATGTACATCTGGCTTTTAAGC
GAGTACACACACATAGCATTGCGATGTTTTTTAAACTTGCACTGCTTGCAAGCAGGGGTCCACTGTATTTCAATGAAAGAACAG
CGTGTTGGGCGTTTTTGAATTATACTAATTACCTTCAATAACAATTCCCTATATATCACTTAGTTTTAATAAATAAATCGTTTGAG
TGTGGTCTAGAGATGATTTATTATGATGAAGTGCGGTTGTTGGGGACAAGCGGGATACGTCTGCGGGTCAAATCGGCGGTGG
GACATAGTGAGAGGGCAACGGCGAACAATCACCAACGCGCCCTGGTACGCGAGAAATGTTGATATCGCACATGACCTCAGCA
TCCACCTTCAGTTTGAGAACAAATTAAATCAGGCACATACCAGACAACCAGGAGAACACTGAGGAGGCGTCATCAAAGATATC
TTTGGACACGTGGCATAAACAAGCCAAACTATTATCTAATCAATATTTTTATACAAATTTTGTATGTTTCCATGTTATCTTCGTTG
CTCCTGTTAGTTACTACGTTTCATAGCCCTTAAATTGGTTGCTTAACGTAAAATAAAATTATATTGTATAAAAATAAAGAGGAAT
TCACCTATAACAAACGAAAAAGTAAATAAATAAGAACTTCCCCATGTTAAAAATGCCACCACCATCTTTACATATAAGTATGTAC
ATATGAATGAATCACTTAGGTTGCTTGAATATTACAATTTCATTAATAGCTAAATCACATTTGATGTGTTAGTGGAAAACGGCTA
TATATATAAATTATCGAAATTGTGAATATCGAATTGCGATAGCACAATGGGAAATTCCACCACTAGATTTTTGGTACTTTTAACA
GATCCTTTTCGGTTTTGCGTTGCGCGAAGTGATCTGAACTTATCAAAGTTTGTAAGGTAATACATAAAGTGAAAAAGAATTAAT
TTGCTCTTGAAAGGCAGGCCAAATTAAAAAAAAAATATCAATCGGTACCAGATCTGGCGCGCCTAGAATGGACTATAAGGACC
ACGACGGAGACTACAAGGATCATGATATTGATTACAAAGACGATGACGATAAGATGGCCCCAAAGAAGAAGCGGAAGGTCG
GTATCCACGGAGTCCCAGCAGCCGACAAGAAGTACAGCATCGGCCTGGACATCGGCACCAACTCTGTGGGCTGGGCCGTGAT
CACCGACGAGTACAAGGTGCCCAGCAAGAAATTCAAGGTGCTGGGCAACACCGACCGGCACAGCATCAAGAAGAACCTGATC
GGAGCCCTGCTGTTCGACAGCGGCGAAACAGCCGAGGCCACCCGGCTGAAGAGAACCGCCAGAAGAAGATACACCAGACGG
AAGAACCGGATCTGCTATCTGCAAGAGATCTTCAGCAACGAGATGGCCAAGGTGGACGACAGCTTCTTCCACAGACTGGAAG
AGTCCTTCCTGGTGGAAGAGGATAAGAAGCACGAGCGGCACCCCATCTTCGGCAACATCGTGGACGAGGTGGCCTACCACGA
GAAGTACCCCACCATCTACCACCTGAGAAAGAAACTGGTGGACAGCACCGACAAGGCCGACCTGCGGCTGATCTATCTGGCCC
TGGCCCACATGATCAAGTTCCGGGGCCACTTCCTGATCGAGGGCGACCTGAACCCCGACAACAGCGACGTGGACAAGCTGTTC
ATCCAGCTGGTGCAGACCTACAACCAGCTGTTCGAGGAAAACCCCATCAACGCCAGCGGCGTGGACGCCAAGGCCATCCTGTC
TGCCAGACTGAGCAAGAGCAGACGGCTGGAAAATCTGATCGCCCAGCTGCCCGGCGAGAAGAAGAATGGCCTGTTCGGAAA
CCTGATTGCCCTGAGCCTGGGCCTGACCCCCAACTTCAAGAGCAACTTCGACCTGGCCGAGGATGCCAAACTGCAGCTGAGCA
AGGACACCTACGACGACGACCTGGACAACCTGCTGGCCCAGATCGGCGACCAGTACGCCGACCTGTTTCTGGCCGCCAAGAA
CCTGTCCGACGCCATCCTGCTGAGCGACATCCTGAGAGTGAACACCGAGATCACCAAGGCCCCCCTGAGCGCCTCTATGATCA
AGAGATACGACGAGCACCACCAGGACCTGACCCTGCTGAAAGCTCTCGTGCGGCAGCAGCTGCCTGAGAAGTACAAAGAGAT
TTTCTTCGACCAGAGCAAGAACGGCTACGCCGGCTACATTGACGGCGGAGCCAGCCAGGAAGAGTTCTACAAGTTCATCAAGC
CCATCCTGGAAAAGATGGACGGCACCGAGGAACTGCTCGTGAAGCTGAACAGAGAGGACCTGCTGCGGAAGCAGCGGACCT
TCGACAACGGCAGCATCCCCCACCAGATCCACCTGGGAGAGCTGCACGCCATTCTGCGGCGGCAGGAAGATTTTTACCCATTC
CTGAAGGACAACCGGGAAAAGATCGAGAAGATCCTGACCTTCCGCATCCCCTACTACGTGGGCCCTCTGGCCAGGGGAAACA
GCAGATTCGCCTGGATGACCAGAAAGAGCGAGGAAACCATCACCCCCTGGAACTTCGAGGAAGTGGTGGACAAGGGCGCTTC
CGCCCAGAGCTTCATCGAGCGGATGACCAACTTCGATAAGAACCTGCCCAACGAGAAGGTGCTGCCCAAGCACAGCCTGCTGT
ACGAGTACTTCACCGTGTATAACGAGCTGACCAAAGTGAAATACGTGACCGAGGGAATGAGAAAGCCCGCCTTCCTGAGCGG
CGAGCAGAAAAAGGCCATCGTGGACCTGCTGTTCAAGACCAACCGGAAAGTGACCGTGAAGCAGCTGAAAGAGGACTACTTC
AAGAAAATCGAGTGCTTCGACTCCGTGGAAATCTCCGGCGTGGAAGATCGGTTCAACGCCTCCCTGGGCACATACCACGATCT
GCTGAAAATTATCAAGGACAAGGACTTCCTGGACAATGAGGAAAACGAGGACATTCTGGAAGATATCGTGCTGACCCTGACA
CTGTTTGAGGACAGAGAGATGATCGAGGAACGGCTGAAAACCTATGCCCACCTGTTCGACGACAAAGTGATGAAGCAGCTGA

AGCGGCGGAGATACACCGGCTGGGGCAGGCTGAGCCGGAAGCTGATCAACGGCATCCGGGACAAGCAGTCCGGCAAGACAA
TCCTGGATTTCCTGAAGTCCGACGGCTTCGCCAACAGAAACTTCATGCAGCTGATCCACGACGATAGCCTGACCTTTAAAGAGG
ACATCCAGAAAGCCCAGGTGTCCGGCCAGGGCGATAGCCTGCACGAGCACATTGCCAATCTGGCCGGCAGCCCCGCCATTAA
GAAGGGCATCCTGCAGACAGTGAAGGTGGTGGACGAGCTCGTGAAAGTGATGGGCCGGCACAAGCCCGAGAACATCGTGAT
CGAAATGGCCAGAGAGAACCAGACCACCCAGAAGGGACAGAAGAACAGCCGCGAGAGAATGAAGCGGATCGAAGAGGGCA
TCAAAGAGCTGGGCAGCCAGATCCTGAAAGAACACCCCGTGGAAAACACCCAGCTGCAGAACGAGAAGCTGTACCTGTACTA
CCTGCAGAATGGGCGGGATATGTACGTGGACCAGGAACTGGACATCAACCGGCTGTCCGACTACGATGTGGACCATATCGTG
CCTCAGAGCTTTCTGAAGGACGACTCCATCGACAACAAGGTGCTGACCAGAAGCGACAAGAACCGGGGCAAGAGCGACAACG
TGCCCTCCGAAGAGGTCGTGAAGAAGATGAAGAACTACTGGCGGCAGCTGCTGAACGCCAAGCTGATTACCCAGAGAAAGTT
CGACAATCTGACCAAGGCCGAGAGAGGCGGCCTGAGCGAACTGGATAAGGCCGGCTTCATCAAGAGACAGCTGGTGGAAAC
CCGGCAGATCACAAAGCACGTGGCACAGATCCTGGACTCCCGGATGAACACTAAGTACGACGAGAATGACAAGCTGATCCGG
GAAGTGAAAGTGATCACCCTGAAGTCCAAGCTGGTGTCCGATTTCCGGAAGGATTTCCAGTTTTACAAAGTGCGCGAGATCAA
CAACTACCACCACGCCCACGACGCCTACCTGAACGCCGTCGTGGGAACCGCCCTGATCAAAAAGTACCCTAAGCTGGAAAGCG
AGTTCGTGTACGGCGACTACAAGGTGTACGACGTGCGGAAGATGATCGCCAAGAGCGAGCAGGAAATCGGCAAGGCTACCG
CCAAGTACTTCTTCTACAGCAACATCATGAACTTTTTCAAGACCGAGATTACCCTGGCCAACGGCGAGATCCGGAAGCGGCCTC
TGATCGAGACAAACGGCGAAACCGGGGAGATCGTGTGGGATAAGGGCCGGGATTTTGCCACCGTGCGGAAAGTGCTGAGCA
TGCCCCAAGTGAATATCGTGAAAAAGACCGAGGTGCAGACAGGCGGCTTCAGCAAAGAGTCTATCCTGCCCAAGAGGAACAG
CGATAAGCTGATCGCCAGAAAGAAGGACTGGGACCCTAAGAAGTACGGCGGCTTCGACAGCCCCACCGTGGCCTATTCTGTG
CTGGTGGTGGCCAAAGTGGAAAAGGGCAAGTCCAAGAAACTGAAGAGTGTGAAAGAGCTGCTGGGGATCACCATCATGGAA
AGAAGCAGCTTCGAGAAGAATCCCATCGACTTTCTGGAAGCCAAGGGCTACAAAGAAGTGAAAAAGGACCTGATCATCAAGC
TGCCTAAGTACTCCCTGTTCGAGCTGGAAAACGGCCGGAAGAGAATGCTGGCCTCTGCCGGCGAACTGCAGAAGGGAAACGA
ACTGGCCCTGCCCTCCAAATATGTGAACTTCCTGTACCTGGCCAGCCACTATGAGAAGCTGAAGGGCTCCCCCGAGGATAATG
AGCAGAAACAGCTGTTTGTGGAACAGCACAAGCACTACCTGGACGAGATCATCGAGCAGATCAGCGAGTTCTCCAAGAGAGT
GATCCTGGCCGACGCTAATCTGGACAAAGTGCTGTCCGCCTACAACAAGCACCGGGATAAGCCCATCAGAGAGCAGGCCGAG
AATATCATCCACCTGTTTACCCTGACCAATCTGGGAGCCCCTGCCGCCTTCAAGTACTTTGACACCACCATCGACCGGAAGAGG
TACACCAGCACCAAAGAGGTGCTGGACGCCACCCTGATCCACCAGAGCATCACCGGCCTGTACGAGACACGGATCGACCTGTC
TCAGCTGGGAGGCGACAAAAGGCCGGCGGCCACGAAAAAGGCCGGCCAGGCAAAAAAGAAAAAGTAATCTAGGCCTGAGCT
CAATGTATGGACATAGATTTCAAATAATTAAATGTAATGCAGTAATTGATGTAATTAGTTAAATAAGTTAGATATTAATAACAT
ATTAATTATATGTATTATAACGCATATAATAATAAAATGCATATTTAGGATATGCAAGCCATTCGAATTTTCTATTTTAATTTCCT
TTTACAAAGAAATGTATAACAAAATATAATTTGAAAAAATGTTCTGGCTCTAATTCGATTTCTTTTAAGTATTTTGTGAACTGCTT
TTAATAACGAGCGGTTGCAAAACTTAACAGAAACGTGCACTTTGATCCCAACTAATACCGTGTACTATCACGTGTTTGGTTTTA
AGTACTCATCCTTCGTGTTTCGTTGTTTGTTGTTTGCCTTGCTGTACACTTGGCTCTTGCGCTCTCTCGCTCTCCGTTGGAGCCGG
CTTTTTGAATGCATGCCTCGCCCTGCTCGCGCCCAGTCCTCCTCCCCCGAAAAGCATGCCGACCAGCAAATGTTGCCCTTTTGCG
TTTGCTGTCTTTGCAGAAGCAAAATCAATAACTGAGAAATCCACCACACTGCTGCTCTTCGTGTTAAGCTTTAAGATACATTGAT
GAGTTTGGACAAACCACAACTAGAATGCAGTGAAAAAAATGCTTTATTTGTGAAATTTGTGATGCTATTGCTTTATTTGTAACC
ATTATAAGCTGCAATAAACAAGTTAACAACAACAATTGCATTCATTTTATGTTTCAGGTTCAGGGGGAGGTGTGGGAGGTTTTT
TAAAGCAAGTAAAACCTCTACAAATGTGGTATGGCTGATTATGATCTAGAGTCGCGGTTACTTGTACAGCTCGTCCATGCCGAG
AGTGATCCCGGCGGCGGTCACGAACTCCAGCAGGACCATGTGATCGCGCTTCTCGTTGGGGTCTTTGCTCAGGGCGGACTGG
GTGCTCAGGTAGTGGTTGTCGGGCAGCAGCACGGGGCCGTCGCCGATGGGGGTGTTCTGCTGGTAGTGGTCGGCGAGCTGC
ACGCTGCCGTCCTCGATGTTGTGGCGGATCTTGAAGTTCACCTTGATGCCGTTCTTCTGCTTGTCGGCCATGATATAGACGTTGT
GGCTGTTGTAGTTGTACTCCAGCTTGTGCCCCAGGATGTTGCCGTCCTCCTTGAAGTCGATGCCCTTCAGCTCGATGCGGTTCA
CCAGGGTGTCGCCCTCGAACTTCACCTCGGCGCGGGTCTTGTAGTTGCCGTCGTCCTTGAAGAAGATGGTGCGCTCCTGGACG
TAGCCTTCGGGCATGGCGGACTTGAAGAAGTCGTGCTGCTTCATGTGGTCGGGGTAGCGGCTGAAGCACTGCACGCCGTAGG
TCAGGGTGGTCACGAGGGTGGGCCAGGGCACGGGCAGCTTGCCGGTGGTGCAGATGAACTTCAGGGTCAGCTTGCCGTAGG
TGGCATCGCCCTCGCCCTCGCCGGACACGCTGAACTTGTGGCCGTTTACGTCGCCGTCCAGCTCGACCAGGATGGGCACCACC
CCGGTGAACAGCTCCTCGCCCTTGCTCACCATCTCGAGCACCAGAGACAGGTTGCGGCGGCGGTTGGATGGCGTGGGCGCGT
TGGCGTTGTTGGACCGGCTCATGTTGTGTCGCTGTAACAGATGCTGTTCAACTGTGTTTACCAGATCGTTGCGGGCTGTATTTA
TAGGCGCGATAAGCGGGACGGGCGCCTCGTGTCCGGTCACGCGCATGAGATAACGCGCGGCTGATATGGAGGCGCGTCCTG
TTCCGATAAGGAGTTGCGTCCGGCTGCGGTTAGCAACACAGGAAGCTGGCGTCCTGTCACGATAAGACAACACTCGTCCGGTC
CGATAATGTGATTCGTACGTGACAGGACGCGACCCGATAAGGCCGGCCTACGTGACTGCCGACACGTACTTTTTTGCACTGCA
AAAAGGTTCAATGTGTGGTAGTGTATTTGGAGCGTATACAACGGTGTAGACTATTTATGTAAAATAGTCTACGAAACGTAGAG
TTTGTACTATGTATGGGCCCGCGTGCAAAAGCGTGTTTTTTTGCAGTGCAAAAAAGTTGGTGGTGGGGAGGCCACCGAGTATG
TTTTTTGCTCACCTGTGATTGCTCCTACTCAAATACAAAAACATCAAATTTTCTGTCAATAAAGCATATTTATTTATATTTATTTA

CAGGAAAGAATTCCTTTTAAAGTGTATTTTAACCTATAATGAAAAACGATTAAAAAAAATACATAAAATAATTCGAAAATTTTT
GAATAGCCCAGGTTGATAAAAATTCATTTCATACGTTTTATAACTTATGCCCCTAAGTATTTTTTGACCATAGTGTTTCAATTCTA
CATTAATTTTACAGAGTAGAATGAAACGCCACCTACTCAGCCAAGAGGCGAAAAGGTTAGCTCGCCAAGCAGAGAGGGCGCC
AGTGCTCACTACTTTTTATAATTCTCAACTTCTTTTTCCAGACTCAGTTCGTATATATAGACCTATTTTCAATTTAACGTC**GACCTA**
CGGCGTGCAGTGACGTTTTAGAGCTAGAAATAGCAAGTTAAAATAAGGCTAGTCCGTTATCAACTTGAAAAAGTGGCACCGA
GTCGGTGCTTTTTTGCCTACCTGGAGCCTGAGAGTTGTTCAATAAAATAAAAATGTTTCGTTTTTTTGCTTTCGCCAGTATTTATT
ATTTTTCATCAATATGTATTCAATTTGGTATGTATTTAGTAATTGTAATATATAGACAATGGTTTTCCGTTGACGTACATACATCT
GACGTGTGTTTATTTAGACATAATAGTTATGTTTTCACATCTTTTTAATGTTCGCTTAATGCGTATGCATTCTAGACAATTGTGCT
CGGCAACAGTATATTTGTGGTGTGCCAACCAACAACCTGCAGGAGCTCCAGCTTTTGT

pGT13_HACK: *nos*-Hack that inserts into AttP sites

white - Backbone

AttB site

Homology Arms

nos-Cas9 cassette

gRNA_HACK expression cassette: **gRNA_HACK (Present only in sGD transgene)**

GATCTCGGATCTGACAATGTTCAGTGCAGAGACTCGGCTACGCCTCGTGGACTTTGAAGTTGACCAACAATGTTTATTCTTACC
TCTAATAGTCCTCTGTGGCAAGGTCAAGATTCTGTTAGAAGCCAATGAAGAACCTGGTTGTTCAATAACATTTTGTTCGTCTAAT
ATTTCACTACCGCTTGACGTTGGCTGCACTTCATGTACCTCATCTATAAACGCTTCTTCTGTATCGCTCTGGACGTCATCTTCACT
TACGTGATCTGATATTTCACTGTCAGAATCCTCACCAACAAGCTCGTCATCGCTTTGCAGAAGAGCAGAGAGGATATGCTCATC
GTCTAAAGAACTACCCATTTTATTATATATTAGTCACGATATCTATAACAAGAAAATATATATATAATAAGTTATCACGTAAGTA
GAACATGAAATAACAATATAATTATCGTATGAGTTAAATCTTAAAAGTCACGTAAAAGATAATCATGCGTCATTTTGACTCACG
CGGTCGTTATAGTTCAAAATCAGTGACACTTACCGCATTGACAAGCACGCCTCACGGGAGCTCCAAGCGGCGACTGAGATGTC
CTAAATGCACAGCGACGGATTCGCGCTATTTAGAAAGAGAGAGCAATATTTCAAGAATGCATGCGTCAATTTTACGCAGACTA
TCTTTCTAGGGTTAAAAAAGATTTGCGCTTTACTCGACCTAAACTTTAAACACGTCATAGAATCTTCGTTTGACAAAAACCACAT
TGTGGCCAAGCTGTGTGACGCGACGCGCGCTAAAGAATGGCAAACCAAGTCGCGCGAGCGTCGACTCTAGAGGATCCCCGGG
TACCGAGCTCGAATTCGTAATCATGGTCATAGCTGTTTCCTGTGTGAAATTGTTATCCGCTCACAATTCCACACAACATACGAGC
CGGAAGCATAAAGTGTAAAGCCTGGGGTGCCTAATGAGTGAGCTAACTCACATTAATTGCGTTGCGCTCACTGCCCGCTTTCC
AGTCGGGAAACCTGTCGTGCCAGCTGCATTAATGAATCGGCCAACGCGCGGGGAGAGGCGGTTTGCGTATTGGGCGCTCTTC
CGCTTCCTCGCTCACTGACTCGCTGCGCTCGGTCGTTCGGCTGCGGCGAGCGGTATCAGCTCACTCAAAGGCGGTAATACGGT
TATCCACAGAATCAGGGGATAACGCAGGAAAGAACATGTGAGCAAAAGGCCAGCAAAAGGCCAGGAACCGTAAAAAGGCCG
CGTTGCTGGCGTTTTTCCATAGGCTCCGCCCCCCTGACGAGCATCACAAAAATCGACGCTCAAGTCAGAGGTGGCGAAACCCG
ACAGGACTATAAAGATACCAGGCGTTTCCCCCTGGAAGCTCCCTCGTGCGCTCTCCTGTTCCGACCCTGCCGCTTACCGGATAC
CTGTCCGCCTTTCTCCCTTCGGGAAGCGTGGCGCTTTCTCAATGCTCACGCTGTAGGTATCTCAGTTCGGTGTAGGTCGTTCGCT
CCAAGCTGGGCTGTGTGCACGAACCCCCCGTTCAGCCCGACCGCTGCGCCTTATCCGGTAACTATCGTCTTGAGTCCAACCCGG
TAAGACACGACTTATCGCCACTGGCAGCAGCCACTGGTAACAGGATTAGCAGAGCGAGGTATGTAGGCGGTGCTACAGAGTT
CTTGAAGTGGTGGCCTAACTACGGCTACACTAGAAGGACAGTATTTGGTATCTGCGCTCTGCTGAAGCCAGTTACCTTCGGAA
AAAGAGTTGGTAGCTCTTGATCCGGCAAACAAACCACCGCTGGTAGCGGTGGTTTTTTTGTTTGCAAGCAGCAGATTACGCGC
AGAAAAAAAGGATCTCAAGAAGATCCTTTGATCTTTTCTACGGGGTCTGACGCTCAGTGGAACGAAAACTCACGTTAAGGGAT
TTTGGTCATGAGATTATCAAAAAGGATCTTCACCTAGATCCTTTTAAATTAAAAATGAAGTTTTAAATCAATCTAAAGTATATAT
GAGTAAACTTGGTCTGACAGTTACCAATGCTTAATCAGTGAGGCACCTATCTCAGCGATCTGTCTATTTCGTTCATCCATAGTTG
CCTGACTCCCCGTCGTGTAGATAACTACGATACGGGAGGGCTTACCATCTGGCCCCAGTGCTGCAATGATACCGCGAGACCCA
CGCTCACCGGCTCCAGATTTATCAGCAATAAACCAGCCAGCCGGAAGGGCCGAGCGCAGAAGTGGTCCTGCAACTTTATCCGC
CTCCATCCAGTCTATTAATTGTTGCCGGGAAGCTAGAGTAAGTAGTTCGCCAGTTAATAGTTTGCGCAACGTTGTTGCCATTGC
TACAGGCATCGTGGTGTCACGCTCGTCGTTTGGTATGGCTTCATTCAGCTCCGGTTCCCAACGATCAAGGCGAGTTACATGATC
CCCCATGTTGTGCAAAAAAGCGGTTAGCTCCTTCGGTCCTCCGATCGTTGTCAGAAGTAAGTTGGCCGCAGTGTTATCACTCAT
GGTTATGGCAGCACTGCATAATTCTCTTACTGTCATGCCATCCGTAAGATGCTTTTCTGTGACTGGTGAGTACTCAACCAAGTCA
TTCTGAGAATAGTGTATGCGGCGACCGAGTTGCTCTTGCCCGGCGTCAATACGGGATAATACCGCGCCACATAGCAGAACTTT

AAAAGTGCTCATCATTGGAAAACGTTCTTCGGGGCGAAAACTCTCAAGGATCTTACCGCTGTTGAGATCCAGTTCGATGTAACC
CACTCGTGCACCCAACTGATCTTCAGCATCTTTTACTTTCACCAGCGTTTCTGGGTGAGCAAAAACAGGAAGGCAAAATGCCGC
AAAAAAGGGAATAAGGGCGACACGGAAATGTTGAATACTCATACTCTTCCTTTTTCAATATTATTGAAGCATTTATCAGGGTTA
TTGTCTCATGAGCGGATACATATTTGAATGTATTTAGAAAAATAAACAAATAGGGGTTCCGCGCACATTTCCCCGAAAAGTGCC
ACCTGACGTCTAAGAAACCATTATTATCATGACATTAACCTATAAAAATAGGCGTATCACGAGGCCCTTTCGTCTCGCGCGTTTC
GGTGATGACGGTGAAAACCTCTGACACATGCAGCTCCCGGAGACGGTCACAGCTTGTCTGTAAGCGGATGCCGGGAGCAGAC
AAGCCCGTCAGGGCGCGTCAGCGGGTGTTGGCGGGTGTCGGGGCTGGCTTAACTATGCGGCATCAGAGCAGATTGTACTGA
GAGTGCACCATATGCGGTGTGAAATACCGCACAGATGCGTAAGGAGAAAATACCGCATCAGGCGCCATTCGCCATTCAGGCT
GCGCAACTGTTGGGAAGGGCGATCGGTGCGGGCCTCTTCGCTATTACGCCAGCTGGCGAAAGGGGGATGTGCTGCAAGGCG
ATTAAGTTGGGTAACGCCAGGGTTTTCCCAGTCACGACGTTGTAAAACGACGGCCAGTGCCAAGCTTTGTTTAAAATATAACA
AAATTGTGATCCCACAAAATGAAGTGGGGCAAAATCAAATAATTAACTAGTGTCCGTAAACTTGTTGGTCTTCAACTTTTTGAG
GAACACGTTGGACGGCAAATCGTGACTATAACACAAGTTGATTTAATAATTTTAGCCAACACGTCGGGCTGCGTGTTTTTTGCG
CTCTGTGTACACGTTGATTAACTGGTCGATTAAATAATTTAATTTTTGGTTCTTCTTTAAATCTGTGATGAAATTTTTTAAAATAA
CTTTAAATTCTTCATTGGTAAAAAATGCCACGTTTTGCAACTTGTGAGGGTCTAATATGAGGTCAAACTCAGTAGGAGTTTTATC
CAAAAAAGAAAACATGATTACGTCTGTACACGAACGCGTATTAACGCAGAGTGCAAAGTATAAGAGGGTTAAAAAATATATTT
TACGCACCATATACGCATCGGGTTGATATCGTTAATATGGATCAATTTGAACAGTTGATTAACGTGTCTCTGCTCAAGTCTTTGA
TCAAAACGCAAATCGACGAAAATGTGTCGGACAATATCAAGTCGATGAGCGAAAAACTAAAAAGGCTAGAATACGACAATCT
CACAGACAGCGTTGAGATATACGGTATTCACGACAGCAGGCTGAATAATAAAAAAATTAGAAACTATTATTTAACCCTAGAAA
GATAATCATATTGTGACGTACGTTAAAGATAATCATGCGTAAAATTGACGCATGTGTTTTATCGGTCTGTATATCGAGGTTTATT
TATTAATTTGAATAGATATTAAGTTTTATTATATTTACACTTACATACTAATAATAAATTCAACAAACAATTTATTTATGTTTATTT
ATTTATTAAAAAAAAACAAAAACTCAAAATTTCTTCTATAAAGTAACAAAACTTTTAAACATTCTCTCTTTTACAAAAATAAACTT
ATTTTGTACTTTAAAAACAGTCATGTTGTATTATAAAATAAGTAATTAGCTTAACTTATACATAATAGAAACAAATTATACTTATT
AGTCAGTCAGAAACAACTTTGGCACATATCAATATTATGCTCTCGACAAATAACTTTTTTGCATTTTTTGCACGATGCATTTGCCT
TTCGCCTTATTTTAGAGGGGCAGTAAGTACAGTAAGTACGTTTTTTCATTACTGGCTCTTCAGTACTGTCATCTGATGTACCAGG
CACTTCATTTGGCAAAATATTAGAGATATTATCGCGCAAATATCTCTTCAAAGTAGGAGCTTCTAAACGCTTACGCATAAACGA
TGACGTCAGGCTCATGTAAAGGTTTCTCATAAATTTTTTGCGACTTTGAACCTTTTCTCCCTTGCTACTGACATTATGGCTGTATA
TAATAAAAGAATTTATGCAGGCAATGTTTATCATTCCGTACAATAATGCCATAGGCCACCTATTCGTCTTCCTACTGCAGGTCAT
CACAGAACACATTTGGTCTAGCGTGTCCACTCCGCCTTTAGTTTGATTATAATACATAACCATTTGCGGTTTACCGGTACTTTCG
TTGATAGAAGCATCCTCATCACAAGATGATAATAAGTATACCATCTTAGCTGGCTTCGGTTTATATGAGACGAGAGTAAGGGG
TCCGTCAAAACAAAACATCGATGTTCCCACTGGCCTGGAGCGACTGTTTTTCAGTACTTCCGGTATCTCGCGTTTGTTTGATCGC
ACGGTTCCCACAATGGTTAATTCGAGCTCGCCCGGGGTCCTAGGTCGACGATGTAGGTCACGGTCTCGAAGCCGCGGTGCGG
GTGCCAGGGCGTGCCCTTGGGCTCCCCGGGCGCGTACTCCACCTCACCCATCTGGTCCATCATGATGAACGGGTCGAGGTGGC
GGTAGTTGATCCCGGCGAACGCGCGGCGCACCGGGAAGCCCTCGCCCTCGAAACCGCTGGGCGCGGTGGTCACGGTGAGCA
CGGGACGTGCGACGGCGTCGGCGGGTGCGGATACGCGGGGCAGCGTCAGCGGGTTCTCGACGGTCACGGCGGGCATGTCG
ACCAAGAAGCCGGTTCAGCTGCCCGGATATTATTATGTGGATACGAAACTGGATATAACTTCGCATAACGAAGACTACACCAT
TGTCGAGCAGTATGAGCGCAGCGAAGGCCGACATCACCTGTTCCTCTACGGCATGGACGAGCTGTACAAGTAGGGTCTTTCGC
CCGCCGCGAAAGCTCTTCAAAGGCAGCAACCAGCAGCGACCAACAAGCATCCATCGAGCTACCCAACAACCTCGGCTCGGACA
GTGATAGACAAAAGCAGCGAACCCATCGCACAACAATTATCATCCAACTCAGATTCACAGCAGATAATCAAAGGCAACCTCCG
GTTGTCGGTGCTCATCCTTCATGGCCATTTCATCGGCAGCGGTATAGCGGATTTTTACTTTGAAGAACTAATCGTAAGAGTCGT
GGCTGTGCTCCATGTCGAGTAGCAATCAAATGTATATGAGGAGCTTTTAACCCTAGTCAGTGAATTGAAAGCCAAATATATCTT
CCATTAAAACTATTAAATATTTTAAATAAATACATTTTTCTATTACCCGCCCCCCCGTGTACTGAAAAGCCCTGGAGGTGCGAAC
CACGTTTGGTTGGTAACAGCTTTAAATTCCTTTTAATTGACATTAAATTAATTGTTAACATTGGTTTTGCATTAGCCTAGCTAGTT
AAATGAAAATACAGCGAGGATACTTGGTACCGGGACTTAGGCGATTTGGGATACAATCGATTGAACGTTGAAAATCTGGCAG
CCGGTCGCCGCGCCGATTTCAGGGCATCCGATTCTTCCAGCTTCTGGGCCTCTTCACGGAACTTTCGGATATTGTCCTTGATCTC
CTTGTTCTTATCCATTTCCGCCTTCATGTTGTCGAAGAACTGCGAGAAGAAGCCGGCCCTGCGACCTGGCGCACTATAAAAGCG
GGGCTGCGGAGTAAAGTCATGACTTAGGCCGGTTGCGCCATGGGGTTTTATGAGCCAAACTCTAACCTGCTGCTGCTGCAGGT
TCTGCGAGGCCTGCTGGCACGTGAAGAGACAGGCGCGATCCCGCACAAGGGCAGCTATTCTGTACTGCAAGGAGAAAAAATC
ATTGAAAGCTTCGACCGTTTTAACCTCGAAATATGCACATGTAAGGACGGATGTGAGCGAACGCCAGTGATGACCGGGATCA
GAGGTAACCTACCATGGTGGGGATTAGGTGACCGTTCGCAGGTAGTTTTATCGGAGCGAATGTTCGGGGGGTCTGGCGTCAG
AGGCTCTAAACTTTATGTAATTCCTGCCGCGAAACACGCACGTATCAAGCAGTCAGCTGTTCTCTTCGTTCAGCGCGCGCCGGT
GTTGCAAAACGAGCGCTCTTCGCCGGCGGTGGCTCGTGCGATAGTTCGTTTTGTCGGTAATCCGATGTTCCCGCGCCGATATCA
TGTGATGTTGTCACAGTGCGCGAAATTCGAATGGTGGTGTGCAGTGATTGTGTTGTGACGGCGAGTGGCGCGTGTGGGTGCT
TAGTTTTGGGAGATGTTTTCGTATTTTTTTGTTGATAACTCAGGCTTTGTTGCTGTGTTGTAATACTATTTTCCATTGCGCGGTGT

CCAGCTTTTAAGTAGTGGCACATATTCTTAGCAAGTAAAAATTATTTTGCATACTATTAAATTTCTTATAAATTATTTTCTAAAAT
TAAGTTTACCTTTTCAATTTTACTAAAAATATCGATATATTTATTATCGCTGGAAAACTACATTATTCCACCTCTAAGCAAGAACC
GTTAGTTGGCGCGTAGCTTTACCACAAAATTCCTGGAATTGCCGTACGCTTCGCAGTTGTTTCAAGTTGTCTAAGGGACATACG
ATTTTTTTTGCCTCTGCGTCACGATTTTAACCCAAAAGCGAGTTTAGTTACATGTACATTATTATTAGATAAAGAAGTATCGCGA
ATACTTCAGTTGAATAAACTGTGCTTGGTTTTTGGGTAAGGATTTGTGGAAAGTAGAGTGCGCGATAACCGTAACTTTCGACCC
GGATTTTCGCCATGGACTATAAGGACCACGACGGAGACTACAAGGATCATGATATTGATTACAAAGACGATGACGATAAGAT
GGCCCCAAAGAAGAAGCGGAAGGTCGGTATCCACGGAGTCCCAGCAGCCGACAAGAAGTACAGCATCGGCCTGGACATCGG
CACCAACTCTGTGGGCTGGGCCGTGATCACCGACGAGTACAAGGTGCCCAGCAAGAAATTCAAGGTGCTGGGCAACACCGAC
CGGCACAGCATCAAGAAGAACCTGATCGGAGCCCTGCTGTTCGACAGCGGCGAAACAGCCGAGGCCACCCGGCTGAAGAGA
ACCGCCAGAAGAAGATACACCAGACGGAAGAACCGGATCTGCTATCTGCAAGAGATCTTCAGCAACGAGATGGCCAAGGTGG
ACGACAGCTTCTTCCACAGACTGGAAGAGTCCTTCCTGGTGGAAGAGGATAAGAAGCACGAGCGGCACCCCATCTTCGGCAA
CATCGTGGACGAGGTGGCCTACCACGAGAAGTACCCCACCATCTACCACCTGAGAAAGAAACTGGTGGACAGCACCGACAAG
GCCGACCTGCGGCTGATCTATCTGGCCCTGGCCCACATGATCAAGTTCCGGGGCCACTTCCTGATCGAGGGCGACCTGAACCC
CGACAACAGCGACGTGGACAAGCTGTTCATCCAGCTGGTGCAGACCTACAACCAGCTGTTCGAGGAAAACCCCATCAACGCCA
GCGGCGTGGACGCCAAGGCCATCCTGTCTGCCAGACTGAGCAAGAGCAGACGGCTGGAAAATCTGATCGCCCAGCTGCCCGG
CGAGAAGAAGAATGGCCTGTTCGGAAACCTGATTGCCCTGAGCCTGGGCCTGACCCCCAACTTCAAGAGCAACTTCGACCTGG
CCGAGGATGCCAAACTGCAGCTGAGCAAGGACACCTACGACGACGACCTGGACAACCTGCTGGCCCAGATCGGCGACCAGTA
CGCCGACCTGTTTCTGGCCGCCAAGAACCTGTCCGACGCCATCCTGCTGAGCGACATCCTGAGAGTGAACACCGAGATCACCA
AGGCCCCCCTGAGCGCCTCTATGATCAAGAGATACGACGAGCACCACCAGGACCTGACCCTGCTGAAAGCTCTCGTGCGGCAG
CAGCTGCCTGAGAAGTACAAAGAGATTTTCTTCGACCAGAGCAAGAACGGCTACGCCGGCTACATTGACGGCGGAGCCAGCC
AGGAAGAGTTCTACAAGTTCATCAAGCCCATCCTGGAAAAGATGGACGGCACCGAGGAACTGCTCGTGAAGCTGAACAGAGA
GGACCTGCTGCGGAAGCAGCGGACCTTCGACAACGGCAGCATCCCCCACCAGATCCACCTGGGAGAGCTGCACGCCATTCTG
CGGCGGCAGGAAGATTTTTACCCATTCCTGAAGGACAACCGGGAAAAGATCGAGAAGATCCTGACCTTCCGCATCCCCTACTA
CGTGGGCCCTCTGGCCAGGGGAAACAGCAGATTCGCCTGGATGACCAGAAAGAGCGAGGAAACCATCACCCCCTGGAACTTC
GAGGAAGTGGTGGACAAGGGCGCTTCCGCCCAGAGCTTCATCGAGCGGATGACCAACTTCGATAAGAACCTGCCCAACGAGA
AGGTGCTGCCCAAGCACAGCCTGCTGTACGAGTACTTCACCGTGTATAACGAGCTGACCAAAGTGAAATACGTGACCGAGGG
AATGAGAAAGCCCGCCTTCCTGAGCGGCGAGCAGAAAAAGGCCATCGTGGACCTGCTGTTCAAGACCAACCGGAAAGTGACC
GTGAAGCAGCTGAAAGAGGACTACTTCAAGAAAATCGAGTGCTTCGACTCCGTGGAAATCTCCGGCGTGGAAGATCGGTTCA
ACGCCTCCCTGGGCACATACCACGATCTGCTGAAAATTATCAAGGACAAGGACTTCCTGGACAATGAGGAAAACGAGGACATT
CTGGAAGATATCGTGCTGACCCTGACACTGTTTGAGGACAGAGAGATGATCGAGGAACGGCTGAAAACCTATGCCCACCTGTT
CGACGACAAAGTGATGAAGCAGCTGAAGCGGCGGAGATACACCGGCTGGGGCAGGCTGAGCCGGAAGCTGATCAACGGCAT
CCGGGACAAGCAGTCCGGCAAGACAATCCTGGATTTCCTGAAGTCCGACGGCTTCGCCAACAGAAACTTCATGCAGCTGATCC
ACGACGACAGCCTGACCTTTAAAGAGGACATCCAGAAAGCCCAGGTGTCCGGCCAGGGCGATAGCCTGCACGAGCACATTGC
CAATCTGGCCGGCAGCCCCGCCATTAAGAAGGGCATCCTGCAGACAGTGAAGGTGGTGGACGAGCTCGTGAAAGTGATGGG
CCGGCACAAGCCCGAGAACATCGTGATCGAAATGGCCAGAGAGAACCAGACCACCCAGAAGGGACAGAAGAACAGCCGCGA
GAGAATGAAGCGGATCGAAGAGGGCATCAAAGAGCTGGGCAGCCAGATCCTGAAAGAACACCCCGTGGAAAACACCCAGCT
GCAGAACGAGAAGCTGTACCTGTACTACCTGCAGAATGGGCGGGATATGTACGTGGACCAGGAACTGGACATCAACCGGCTG
TCCGACTACGATGTGGACCATATCGTGCCTCAGAGCTTTCTGAAGGACGACTCCATCGACAACAAGGTGCTGACCAGAAGCGA
CAAGAACCGGGGCAAGAGCGACAACGTGCCCTCCGAAGAGGTCGTGAAGAAGATGAAGAACTACTGGCGGCAGCTGCTGAA
CGCCAAGCTGATTACCCAGAGAAAGTTCGACAATCTGACCAAGGCCGAGAGAGGCGGCCTGAGCGAACTGGATAAGGCCGG
CTTCATCAAGAGACAGCTGGTGGAAACCCGGCAGATCACAAAGCACGTGGCACAGATCCTGGACTCCCGGATGAACACTAAG
TACGACGAGAATGACAAGCTGATCCGGGAAGTGAAAGTGATCACCCTGAAGTCCAAGCTGGTGTCCGATTTCCGGAAGGATT
TCCAGTTTTACAAAGTGCGCGAGATCAACAACTACCACCACGCCCACGACGCCTACCTGAACGCCGTCGTGGGAACCGCCCTG
ATCAAAAAGTACCCTAAGCTGGAAAGCGAGTTCGTGTACGGCGACTACAAGGTGTACGACGTGCGGAAGATGATCGCCAAGA
GCGAGCAGGAAATCGGCAAGGCTACCGCCAAGTACTTCTTCTACAGCAACATCATGAACTTTTTCAAGACCGAGATTACCCTG
GCCAACGGCGAGATCCGGAAGCGGCCTCTGATCGAGACAAACGGCGAAACCGGGGAGATCGTGTGGGATAAGGGCCGGGA
TTTTGCCACCGTGCGGAAAGTGCTGAGCATGCCCCAAGTGAATATCGTGAAAAAGACCGAGGTGCAGACAGGCGGCTTCAGC
AAAGAGTCTATCCTGCCCAAGAGGAACAGCGATAAGCTGATCGCCAGAAAGAAGGACTGGGACCCTAAGAAGTACGGCGGC
TTCGACAGCCCCACCGTGGCCTATTCTGTGCTGGTGGTGGCCAAAGTGGAAAAGGGCAAGTCCAAGAAACTGAAGAGTGTGA
AAGAGCTGCTGGGGATCACCATCATGGAAAGAAGCAGCTTCGAGAAGAATCCCATCGACTTTCTGGAAGCCAAGGGCTACAA
AGAAGTGAAAAAGGACCTGATCATCAAGCTGCCTAAGTACTCCCTGTTCGAGCTGGAAAACGGCCGGAAGAGAATGCTGGCC
TCTGCCGGCGAACTGCAGAAGGGAAACGAACTGGCCCTGCCCTCCAAATATGTGAACTTCCTGTACCTGGCCAGCCACTATGA
GAAGCTGAAGGGCTCCCCCGAGGATAATGAGCAGAAACAGCTGTTTGTGGAACAGCACAAGCACTACCTGGACGAGATCATC

GAGCAGATCAGCGAGTTCTCCAAGAGAGTGATCCTGGCCGACGCTAATCTGGACAAAGTGCTGTCCGCCTACAACAAGCACC
GGGATAAGCCCATCAGAGAGCAGGCCGAGAATATCATCCACCTGTTTACCCTGACCAATCTGGGAGCCCCTGCCGCCTTCAAG
TACTTTGACACCACCATCGACCGGAAGAGGTACACCAGCACCAAAGAGGTGCTGGACGCCACCCTGATCCACCAGAGCATCAC
CGGCCTGTACGAGACACGGATCGACCTGTCTCAGCTGGGAGGCGACAAAAGGCCGGCGGCCACGAAAAAGGCCGGCCAGGC
AAAAAAGAAAAAGTAAAGAGGGCGAATCCAGCTCTGGAGCAGAGGCTCTGGCAGCTTTTGCAGCGTTTATATAACATGAAAT
ATATATACGCATTCCGATCAAAGCTGGGTTAACCAGATAGATAGTAACGTTTAACTAGCGCCTGGCGCGTTCGATTTTAAAGAG
ATTTAGAGCGTTATCCCGTGCCTATAGATCTTATAGTATAGACAACGAACGATCACTCAAATCCAAGTCAATAATTCAAGAATTT
ATGTCTGTTTCTGTGAAAGGGAAACTAATTTTGTTAAAGAAGACTTACAATATCGTAATACTTGTTCAATCGTCGTGGCCGATA
GAAATATCTTACAATCCGAAAGTTGATGAATGGAATTGGTCTGCAACTGGTCGCCTTCATTTTGTAAAATGTTCGCTTGCGGCC
GAAAAATTTCGATATATCTACAATTGATATACAATCTTTACTAAATTTTGAAAAAGGAACACTTTGAATTTCGAACTGTCAATCG
TATCATTAGAATTTAATCTAAATTTAAATCTTGCTAAAGGAAATAGCAAGGAACACTTTCGTCGTCGGCTACGCATTCATTGTAA
AATTTTAAATTTTGACATTCCGCACTTTTTGATAGATAAGCGAAGAGTATTTTTATTACATGTATCGCAAGTATTCATTTCAACAC
ACATATCTATATATATATATATATATATATATATATATATATATATATGTTATATATTTATTCAATTTTGTTTACCATTGATCAAT
TTTTCACACATGAAACAACCGCCAGCATTATATAATTTTTTTATTTTTTTAAAAAATGTGTACACATATTCTGAAAATGAAAAATT
CAATGGCTCGAGTGCCAAATAAAGAAATGGTTACAATTTAAGGAAACAAATGTCCTTCTTGCGTTTGAAACAACTAATCCTTTT
CGCCCTCGCGGCGTTTCTCGAAAAGGGCCAGGAAGATGCCATCGAAGCTTTAAGATACATTGATGAGTTTGGACAAACCACAA
CTAGAATGCAGTGAAAAAAATGCTTTATTTGTGAAATTTGTGATGCTATTGCTTTATTTGTAACCATTATAAGCTGCAATAAACA
AGTTAACAACAACAATTGCATTCATTTTATGTTTCAGGTTCAGGGGGAGGTGTGGGAGGTTTTTTAAAGCAAGTAAAACCTCTA
CAAATGTGGTATGGCTGATTATGATCTAGAGTCGCGGTTACTTGTACAGCTCGTCCATGCCGAGAGTGATCCCGGCGGCGGTC
ACGAACTCCAGCAGGACCATGTGATCGCGCTTCTCGTTGGGGTCTTTGCTCAGGGCGGACTGGGTGCTCAGGTAGTGGTTGTC
GGGCAGCAGCACGGGGCCGTCGCCGATGGGGGTGTTCTGCTGGTAGTGGTCGGCGAGCTGCACGCTGCCGTCCTCGATGTTG
TGGCGGATCTTGAAGTTCACCTTGATGCCGTTCTTCTGCTTGTCGGCCATGATATAGACGTTGTGGCTGTTGTAGTTGTACTCCA
GCTTGTGCCCCAGGATGTTGCCGTCCTCCTTGAAGTCGATGCCCTTCAGCTCGATGCGGTTCACCAGGGTGTCGCCCTCGAACT
TCACCTCGGCGCGGGTCTTGTAGTTGCCGTCGTCCTTGAAGAAGATGGTGCGCTCCTGGACGTAGCCTTCGGGCATGGCGGAC
TTGAAGAAGTCGTGCTGCTTCATGTGGTCGGGGTAGCGGCTGAAGCACTGCACGCCGTAGGTCAGGGTGGTCACGAGGGTG
GGCCAGGGCACGGGCAGCTTGCCGGTGGTGCAGATGAACTTCAGGGTCAGCTTGCCGTAGGTGGCATCGCCCTCGCCCTCGC
CGGACACGCTGAACTTGTGGCCGTTTACGTCGCCGTCCAGCTCGACCAGGATGGGCACCACCCCGGTGAACAGCTCCTCGCCC
TTGCTCACCATCTCGAGCACCAGAGACAGGTTGCGGCGGCGGTTGGATGGCGTGGGCGCGTTGGCGTTGTTGGACCGGCTCA
TGTTGTGTCGCTGTAACAGATGCTGTTCAACTGTGTTTACCAGATCGTTGCGGGCTGTATTTATAGGCGCGATAAGCGGGACG
GGCGCCTCGTGTCCGGTCACGCGCATGAGATAACGCGCGGCTGATATGGAGGCGCGTCCTGTTCCGATAAGGAGTTGCGTCC
GGCTGCGGTTAGCAACACAGGAAGCTGGCGTCCTGTCACGATAAGACAACACTCGTCCGGTCCGATAATGTGATTCGTACGTG
ACAGGACGCGACCCGATAAGGCCGGCCTACGTGACTGCCGACACGTACTTTTTTGCACTGCAAAAAGGTTCAATGTGTGGTAG
TGTATTTGGAGCGTATACAACGGTGTAGACTATTTATGTAAAATAGTCTACGAAACGTAGAGTTTGTACTATGTATGGGCCCGC
GTGCAAAAGCGTGTTTTTTTGCAGTGCAAAAAAGTTGGTGGTGGGGAGGCCACCGAGTATGTTTTTTGCTCACCTGTGATTGC
TCCTACTCAAATACAAAAACATCAAATTTTCTGTCAATAAAGCATATTTATTTATATTTATTTTACAGGAAAGAATTCCTTTTAAA
GTGTATTTTAACCTATAATGAAAAACGATTAAAAAAAATACATAAAATAATTCGAAAATTTTTGAATAGCCCAGGTTGATAAAA
ATTCATTTCATACGTTTTATAACTTATGCCCCTAAGTATTTTTTGACCATAGTGTTTCAATTCTACATTAATTTTACAGAGTAGAAT
GAAACGCCACCTACTCAGCCAAGAGGCGAAAAGGTTAGCTCGCCAAGCAGAGAGGGCGCCAGTGCTCACTACTTTTTATAATT
CTCAACTTCTTTTTCCAGACTCAGTTCGTATATATAGACCTATTTTCAATTTAACGTC**GACCTACGGCGTGCAGTGAC**GTTTTAG
AGCTAGAAATAGCAAGTTAAAATAAGGCTAGTCCGTTATCAACTTGAAAAAGTGGCACCGAGTCGGTGCTTTTTTGCCTACCTG
GAGCCTGAGAGTTGTTCAATAAAATAAAAATGTTTCGTTTTTTTGCTTTCGCCAGTATTTATTATTTTTCATCAATATGTATTCAA
TTTGGTATGTATTTAGTAATTGTAATATATAGACAATGGTTTTCCGTTGACGTACATACATCTGACGTGTGTTTATTTAGACATA
ATAGTTATGTTTTCACATCTTTTTAATGTTCGCTTAATGCGTATGCATTCTAGACAATTGTGCTCGGCAACAGTATATTTGTGGTG
TGCCAACCAACAACCTGCAGGAGCTCCAGCTTTTGT

CC*vasa*: CopyCat (split) element inserted in the *vasa* gene and used for the assessment of "static vs Hack" Cas9 lines.

Backbone Homology arms Recoded vasa T2A or P2A dsRed (first is recoded, second is generic) SV40 U6 cassette mCerulean cassette

GGTATCAGCTCACTCAAAGGCGGTAATACGGTTATCCACAGAATCAGGGGATAACGCAGGAAAGAACATGTGAGCAAAAGGC
CAGCAAAAGGCCAGGAACCGTAAAAAGGCCGCGTTGCTGGCGTTTTTCCATAGGCTCCGCCCCCCTGACGAGCATCACAAAAA
TCGACGCTCAAGTCAGAGGTGGCGAAACCCGACAGGACTATAAAGATACCAGGCGTTTCCCCCTGGAAGCTCCCTCGTGCGCT
CTCCTGTTCCGACCCTGCCGCTTACCGGATACCTGTCCGCCTTTCTCCCTTCGGGAAGCGTGGCGCTTTCTCATAGCTCACGCTG
TAGGTATCTCAGTTCGGTGTAGGTCGTTCGCTCCAAGCTGGGCTGTGTGCACGAACCCCCCGTTCAGCCCGACCGCTGCGCCTT
ATCCGGTAACTATCGTCTTGAGTCCAACCCGGTAAGACACGACTTATCGCCACTGGCAGCAGCCACTGGTAACAGGATTAGCA
GAGCGAGGTATGTAGGCGGTGCTACAGAGTTCTTGAAGTGGTGGCCTAACTACGGCTACACTAGAAGAACAGTATTTGGTAT
CTGCGCTCTGCTGAAGCCAGTTACCTTCGGAAAAAGAGTTGGTAGCTCTTGATCCGGCAAACAAACCACCGCTGGTAGCGGTG
GTTTTTTTGTTTGCAAGCAGCAGATTACGCGCAGAAAAAAAGGATCTCAAGAAGATCCTTTGATCTTTTCTACGGGGTCTGACG
CTCAGTGGAACGAAAACTCACGTTAAGGGATTTTGGTCATGAGATTATCAAAAAGGATCTTCACCTAGATCCTTTTAAATTAAA
AATGAAGTTTTAAATCAATCTAAAGTATATATGAGTAAACTTGGTCTGACAGTTACCAATGCTTAATCAGTGAGGCACCTATCT
CAGCGATCTGTCTATTTCGTTCATCCATAGTTGCCTGACTCCCCGTCGTGTAGATAACTACGATACGGGAGGGCTTACCATCTG
GCCCCAGTGCTGCAATGATACCGCGAGACCCACGCTCACCGGCTCCAGATTTATCAGCAATAAACCAGCCAGCCGGAAGGGCC
GAGCGCAGAAGTGGTCCTGCAACTTTATCCGCCTCCATCCAGTCTATTAATTGTTGCCGGGAAGCTAGAGTAAGTAGTTCGCCA
GTTAATAGTTTGCGCAACGTTGTTGCCATTGCTACAGGCATCGTGGTGTCACGCTCGTCGTTTGGTATGGCTTCATTCAGCTCC
GGTTCCCAACGATCAAGGCGAGTTACATGATCCCCCATGTTGTGCAAAAAAGCGGTTAGCTCCTTCGGTCCTCCGATCGTTGTC
AGAAGTAAGTTGGCCGCAGTGTTATCACTCATGGTTATGGCAGCACTGCATAATTCTCTTACTGTCATGCCATCCGTAAGATGC
TTTTCTGTGACTGGTGAGTACTCAACCAAGTCATTCTGAGAATAGTGTATGCGGCGACCGAGTTGCTCTTGCCCGGCGTCAATA
CGGGATAATACCGCGCCACATAGCAGAACTTTAAAAGTGCTCATCATTGGAAAACGTTCTTCGGGGCGAAAACTCTCAAGGAT
CTTACCGCTGTTGAGATCCAGTTCGATGTAACCCACTCGTGCACCCAACTGATCTTCAGCATCTTTTACTTTCACCAGCGTTTCTG
GGTGAGCAAAAACAGGAAGGCAAAATGCCGCAAAAAAGGGAATAAGGGCGACACGGAAATGTTGAATACTCATACTCTTCCT
TTTTCAATATTATTGAAGCATTTATCAGGGTTATTGTCTCATGAGCGGATACATATTTGAATGTATTTAGAAAAATAAACAAATA
GGGGTTCCGCGCACATTTCCCCGAAAAGTGCCACCTGACGTCTAAGAAACCATTATTATCATGACATTAACCTATAAAAATAGG
CGTATCACGAGGCCCTTTCCGAGGCGAGGAAGTTTGCTTTCGAGTCATATCTAAAGATCGGTATTGTTTACGGAGGCACCTCGT
TCAGACATCAAAACGAGTGCATTACCAGAGGCTGCCATGTAGTGATCGCCACTCCGGGACGACTTCTGGATTTCGTGGATCGG
ACCTTTATCACGTTTGAAGACACTCGATTCGTTGTGCTGGATGAGGCCGATCGCATGCTAGATATGGGTTTCTCAGAAGACATG
CGAAGAATCATGACGCATGTAACTATGCGTCCAGAACATCAGACATTGATGTTTTCCGCCACGTTTCCAGAAGAAATCCAAAGA
ATGGCCGGCGAATTCCTTAAAAATTACGTTTTCGTCGCCATTGGCATTGTAGGCGGAGCTTGCTCTGATGTGAAGCAGACCATA
TACGAAGTTAATAAGTACGCCAAGCGATCCAAGCTAATAGTAAGTGGTCCTACAAATTAAAGCCCAACACCAATTTGTTATATT
CTATTCTCTTTAGGAAATCCTTTCCGAGCAAGCAGATGGCACCATTGTGTTTGTGGAGACAAAGCGTGGCGCCGACTTTTTAGC
TTCCTTCCTGTCAGAAAAGGAGTTTCCGACGACCTCCATTCATGGCGATCGTCTCCAGAGTCAACGCGAGCAGGCCTTGCGTGA
TTTCAAGAACGGCTCTATGAAGGTTCTCATAGCCACTTCAGTGGCTTCTCGTGGATTAGGTAAGTTAGGACGCTCACACTTTCC
TGAATCAAATTTCATTAAGTTCTTTTTTCATCTTGCTAGACATTAAAAACATCAAGCATGTGATCAACTATGACATGCCCAGCAA
GATCGATGACTACGTACATCGCATTGGACGTACAGGTCGTGTAGGCAATAATGGACGAGCCACAAGCTTCTTTGACCCTGAAA
AGGATCGAGCTATTGCTGCGGACTTGGTAAAAATCTTGGAGGGATCTGGCCAGACTGTTCCGGACTTTCTACGCACCTGTGGT
GCCGGCGGTGATGGGGGTTACTCCAATCAAAATTTTGGCGGCGTTGACGTTCGCGGTCGCGGCAACTATGTGGGTGACGCGA
CGAACGTGGAAGAGGAGGAACAGTGGGACGAGGGCCGCGGCAGCCTGCTGACCTGCGGCGATGTGGAGGAGAACCCCGGG
CCCATGGCGAGCAGCGAAAATGTGATTACGGAATTTATGCGATTTAAAGTTCGTATGGAAGGAACGGTCAATGGACACGAAT
TTGAAATTGAAGGAGAAGGAGAAGGACGGCCATATGAAGGACATAATACGGTCAAATTGAAAGTCACGAAAGGAGGACCGT
TGCCGTTTGCGTGGGATATTTTGTCGCCGCAATTTCAATATGGAAGCAAAGTCTATGTCAAACATCCGGCGGATATTCCGGATT
ATAAAAAATTGAGCTTTCCGGAAGGATTTAAATGGGAACGTGTCATGAATTTTGAAGATGGAGGAGTCGCCACGGTCACGCA
AGATAGCAGCTTGCAAGATGGATGTTTTATTTATAAAGTCAAATTTATTGGAGTCAATTTTCCGAGCGATGGACCGGTCATGCA
AAAAAAAACGATGGGATGGGAAGCGAGCACGGAACGTTTGTATCCGCGTGATGGAGTCTTGAAAGGAGAAACACATAAAGC
TTTGAAATTGAAAGATGGAGGACATTATTTGGTCGAATTTAAAAGCATTTATATGGCGAAAAAACCGGTCCAATTGCCGGGAT
ATTATTATGTCGATGCGAAATTGGATATTACGAGCCATAATGAAGATTATACGATTGTCGAACAATATGAACGTACGGAAGGT
CGGCATCATTTGTTTTTGCGCAGCCGCCCGAAAAAGAAACGCAAAGTCGAAGATCCCGAGGGCAGAGGAAGTCTTCTAACAT
GCGGCGATGTGGAGGAGAATCCCGGCCCTATGGCCTCCTCCGAGAACGTCATCACCGAGTTCATGCGCTTCAAGGTGCGCATG

GAGGGCACCGTGAACGGCCACGAGTTCGAGATCGAGGGCGAGGGCGAGGGCCGCCCCTACGAGGGCCACAACACCGTGAA
GCTGAAGGTGACCAAGGGCGGCCCCCTGCCCTTCGCCTGGGACATCCTGTCCCCCCAGTTCCAGTACGGCTCCAAGGTGTACG
TGAAGCACCCCGCCGACATCCCCGACTACAAGAAGCTGTCCTTCCCCGAGGGCTTCAAGTGGGAGCGCGTGATGAACTTCGAG
GACGGCGGCGTGGCGACCGTGACCCAGGACTCCTCCCTGCAGGACGGCTGCTTCATCTACAAGGTGAAGTTCATCGGCGTGA
ACTTCCCCTCCGACGGCCCCGTGATGCAGAAGAAGACCATGGGCTGGGAGGCCTCCACCGAGCGCCTGTACCCCCGCGACGG
CGTGCTGAAGGGCGAGACCCACAAGGCCCTGAAGCTGAAGGACGGCGGCCACTACCTGGTGGAGTTCAAGTCCATCTACATG
GCCAAGAAGCCCGTGCAGCTGCCCGGCTACTACTACGTGGACGCCAAGCTGGACATCACCTCCCACAACGAGGACTACACCAT
CGTGGAGCAGTACGAGCGCACCGAGGGCCGCCACCACCTGTTCCTGAGATCTCGACCCAAGAAAAAGCGGAAGGTGGAGGA
CCCGTAAGATCCACCGGATCTAGATAACTGATCATAATCAGCCATACCACATTTGTAGAGGTTTTACTTGCTTTAAAAAACCTCC
CACACCTCCCCCTGAACCTGAAACATAAAATGAATGCAATTGTTGTTGTTAACTTGTTTATTGCAGCTTATAATGGTTACAAATA
AAGCAATAGCATCACAAATTTCACAAATAAAGCATTTTTTTCACTGCATTCTAGTTGTGGTTTGTCCAAACTCATCAATGTATCTT
ACCTTTTTTGCTCACCTGTGATTGCTCCTACTCAAATACAAAAACATCAAATTTTCTGTCAATAAAGCATATTTATTTATATTTATT
TTACAGGAAAGAATTCCTTTTAAAGTGTATTTTAACCTATAATGAAAAACGATTAAAAAAAATACATAAAATAATTCGAAAATTT
TTGAATAGCCCAGGTTGATAAAAATTCATTTCATACGTTTTATAACTTATGCCCCTAAGTATTTTTTGACCATAGTGTTTCAATTC
TACATTAATTTTACAGAGTAGAATGAAACGCCACCTACTCAGCCAAGAGGCGAAAAGGTTAGCTCGCCAAGCAGAGAGGGCG
CCAGTGCTCACTACTTTTTATAATTCTCAACTTCTTTTTCCAGACTCAGTTCGTATATATAGACCTATTTTCAATTTAACGTCGCTC
CAATCAAAATTTCGGTGGTTTTAGAGCTAGAAATAGCAAGTTAAAATAAGGCTAGTCCGTTATCAACTTGAAAAAGTGGCACC
GAGTCGGTGCTTTTTTGCCTACCTGGAGCCTGAGAGTTGTTCAATAAAATAAAAATGTTTCGTTTTTTTGCTTTCGCCAGTATTT
ATTATTTTTCATCAATATGTATTCAATTTGGTATGTATTTAGTAATTGTAATATATAGACAATGGTTTTCCGTTGACGTACATACA
TCTGACGTGTGTTTATTTAGACATAATAGTTATGTTTTCACATCTTTTTAATGTTCGCTTAATGCGTATGCATTTAGACATAACTT
CGTATAGCATACATTATACGAAGTTATGATCGATCCCACGCGCTTGAAAGGAGTGTGTAAATGGACAATGTTGTTACAATAAA
AGTTGAAATTATTTTATAAAATTTATTAAAAACATATTTAAACATAACATATTGTATTAAAACATGACAATACAAACTAAGATTT
AGTCAGATAAACCTATTTGTAGAGTTCGTCCATACCCAAAGTAATTCCTGCCGCGGTCACGAACTCCAGCAACACCATATGATC
CCTCTTTTCGTTGGGATCCTTACTGAGTTTACTCTGCGTGCTCAGGTAGTGGTTGTCAGGCAAGAGCACGGGACCGTCGCCGAT
AGGTGTATTTTGCTGATAATGATCGGCCAATTGGACCGATCCATCTTCTATGTTACAATTCAGACCGAAATTCGCTTTGATACCG
TTTTTCTGTTTATCGGCTGTTATATAAACATTTCCATGGATAGCGTTATATTCCAACTTATGGCCCAGAATATTACCATCTTCCTT
AAAGTCTATACCCTTCAACTCGATACGATTCACCAGTGTATCACCTTCAAACTTGACCTCTGCCCGTGTTTTGTAGTTGCCATCGT
CTTTGAAAAATATTGTCCGTTCCTGCACATAACCTTCTGGCATTGCACTTTTAAAGAAGTCGTGCTGCTTCATGTGGTCCGGGTA
ACGAGCGAAACACTGAACTCCCCACGAGAGTGTAGTCACGAGTGTCGGCCAGGGTACAGGCAGTTTGCCCGTGGTGCAAATG
AATTTCAGGGTCAGTTTACCGTAAGTAGCATCTCCTTCACCTTCTCCACTGACACTGAATTTGTGACCGTTCACGTCACCATCCA
ATTCGACGAGTATCGGGACCACGCCAGTGAAGAGTTCTTCACCTTTACTCACCATGGTGGCGACCGGCTTCGAGCCGATTGTTT
AGCTTGTTCAGCTGCGCTTGTTTATTTGCTTAGCTTTCGCTTAGCGACGTGTTCACTTTGCTTGTTTGAATTGAATTGTCGCTCCG
TAGACGAAGCGCCTCTATTTATACTCCGGCGGTCGAGGGTTCGAAATCGATAAGCTTGGATCCTAATTGAATTAGCTCTAATTG
AATTAGTCTCTAATTGAATTAGATCCCCGGGCGAGCTCGCCTAGGATAACTTCGTATAGCATACATTATACGAAGTTATCTACG
CCCCCAACTGAGAGAACTCAAAGGTTACCCCAGTTGGGGCACTACGTCAAGTGGGGTGGATGTGCGTGGCAGGGTGAGTAAA
CTGGAAAAACTTCTTTTGTTTTGCACTTCCAGCTTACTGAATTTTTCATTTTTCTTATACAGGGAAATTACGTCGGCGATGCCACC
AATGTCGAGGAAGAAGAGCAATGGGATTGAAATGTATGGACATAGATTTCAAATAATTAAATGTAATGCAGTAATTGATGTAA
TTAGTTAAATAAGTTAAATATTAATAACATATTAATTATATGTATTATAACGCATATAATAATAAAATGCATATTTAGGATATGC
AAGCCATTCGAATTTTCTATTTTAATTTCCTTTTACAAAGAAATGTATAACAAAATATAATTTGAAAAAATGTTCTGGCTCTAATT
CGATTTCTTTTAAGTATTTTGTGAACTGCTTTTAATAACGAGCGGTTGCAAAACTTAACAGAAACGTGCACTTTGATCCCAACTA
ATACCGTGTACTATCATGTGTTTGGTTTTAAGTACTCATCCTTCGTGTTTCGTTGTTTGTTGTTTGCCTTGCTGTACACTTGGCTCT
TGCGCTCTCTCGCTCTCCGTTGGAGCCGGCTTTTTGAATGCATGCCTCGCCCTGCTCGCGCCCAGTCCTCCTCCCCCGAAAAGCA
TGCCGACCAGCAAATGTTGCCCTTTTGCGTTTGCTGTCTTTGCAGAAGCAAAATCAATAACTGAGAAATCCACCACACTGCTGC
TCTTCGTGTTGAATTCTCAACTTCCGCAGCAAAGTGAAAGACATTTCAGTCTGACGGCATTTCGTAGCGCGCAAACACTTTCTTC
GCGTCGCGCTCCCCATGACATATTTCCAAATAAAAGGAAAAAGCTGTGAAAGTAATAGTGGTTAATTGTTTTTAAGTAAAACG
AATTTCTATTGTGATTAAAGCGCTTGGTTCCGTTGCGTACGATAGTGTTTTTCGTTAAGTGACAAATAAAATCGTGCTCTTGTTA
ATCTAAAAATTGTTTAACCTAAATGCGCAAAGCGATTATGAAATAAGAAAGACATTTGAATTTTGTCTTTGTTTTCACAAGGGTT
TTTCGATTCACTCCTTTTCACGC

References

1 Terradas, G. *et al.* Inherently confinable split-drive systems in *Drosophila*. *Nat Commun* **12**, 1480 (2021). https://doi.org:10.1038/s41467-021-21771-7

2 Sánchez C., H. M., Wu, S. L., Bennett, J. B. & Marshall, J. M. MGDrivE: A modular simulation framework for the spread of gene drives through spatially explicit mosquito populations. *Methods in Ecol Evol* **11**, 229-239 (2020). https://doi.org:10.1111/2041-210x.13318

3 Gantz, V. M. & Bier, E. The dawn of active genetics. *Bioessays* **38**, 50-63 (2016). https://doi.org:10.1002/bies.201500102
